# Titration of 124 antibodies using CITE-Seq on human PBMCs

**DOI:** 10.1038/s41598-022-24371-7

**Published:** 2022-12-02

**Authors:** Felix Sebastian Nettersheim, Sujit Silas Armstrong, Christopher Durant, Rafael Blanco-Dominguez, Payel Roy, Marco Orecchioni, Vasantika Suryawanshi, Klaus Ley

**Affiliations:** 1 La Jolla Institute for Immunology, La Jolla, CA 92037 USA; 2 Department of Cardiology, Faculty of Medicine and University Hospital Cologne, University of Cologne, 50937 Cologne, Germany; 3 Centro Nacional de Investigaciones Cardiovasculares, 28029 Madrid, Spain; 4 Department of Bioengineering, University of California, San Diego, San Diego, CA 92093 USA; 5 Immunology Center of Georgia (IMMCG), Augusta University, Augusta, GA 30912 USA

**Keywords:** Immunology, RNA sequencing

## Abstract

Single-cell RNA-sequencing (scRNA-Seq) is widely used to characterize immune cell populations. However, mRNA levels correlate poorly with expression of surface proteins, which are well established to define immune cell types. CITE-Seq (cellular indexing of transcriptomes and epitopes by sequencing) utilizes oligonucleotide-tagged antibodies to simultaneously analyze surface phenotypes and transcriptomes. Considering the high costs of adding surface phenotyping to scRNA-Seq, we aimed to determine which of 188 tested CITE-Seq antibodies can detect their antigens on human peripheral blood mononuclear cells (PBMCs), a commonly interrogated cell population in immunology, and find the optimal concentration for staining. The recommended concentration was optimal for 76 antibodies, whereas staining quality of 7 antibodies improved when the concentration was doubled. 33 and 8 antibodies still worked well when the concentration was reduced to 1/5 or 1/25, respectively. 64 antigens were not detected at any antibody concentration. Optimizing the antibody panel by removing antibodies not able to detect their target antigens and adjusting concentrations of the remaining antibodies will improve the analysis and may reduce costs. In conclusion, our data are a resource for building an informative and cost-effective panel of CITE-Seq antibodies and use them at their optimal concentrations in future CITE-seq experiments on human PBMCs.

## Introduction

Single cell RNA sequencing (scRNA-Seq) has revolutionized the analysis of immune cells in humans and animal models^1–5^. Traditionally, immune cells are characterized by their expression of surface proteins, transcription factors and intracellular cytokines^6^. Spectral flow cytometry (FACS) and mass cytometry (CyTOF) can detect 40–50 markers^7,8^ and thus enable a thorough phenotyping of immune cell populations. However, scRNA-Seq provides a (partial) transcriptome, and thus facilitates detection of rare and hitherto unknown cell types as well as an in-depth characterization of population heterogeneity^9^. In recent years, scRNA-Seq has successfully been utilized to define gene signatures of many cell types^10–17^. However, the correlation between protein and mRNA expression is overall quite weak^17–19^. Fewer than 50% of surface proteins on human peripheral mononuclear cells (PBMCs) correlate with the mRNA of their encoding genes^17^. Therefore, it is beneficial to analyze cell surface phenotype along with transcriptomes.

To address this problem, two approaches were introduced in 2017: CITE-Seq (cellular indexing of transcriptomes and epitopes by sequencing)^20^ and REAP-Seq (RNA expression and protein sequencing assay)^21^, which were developed by Stoeckius and colleagues at the New York Genome Center and Peterson et al. at the Merck Department for Translational Medicine, respectively. Both methods detect surface proteins through utilization of oligonucleotide-tagged antibodies. Such antibodies have become commercially available for the 10 × Genomics Chromium™ (BioLegend® TotalSeq™) and BD® Rhapsody™ scRNA-Seq systems (BD® AbSeq). Adding cell surface phenotype assessment to scRNA-Seq is informative, but at least doubles the cost per sample. The main cost drivers are the antibody pools, the extra PCR steps required for library preparation, the additional sequencing costs, and the labor cost for cell washing and counting. At current market prices, a CITE-Seq experiment using the 10 × Genomics system and their 137plex TotalSeq™ human universal antibody cocktail costs around $3000 per sample. This amount does not include labor costs and consists of around $1000 each for reagents, sequencing, and antibodies. Addition of more antibodies increases the costs even further.

Although antibodies are titrated by the manufacturer using flow cytometry, almost no data are available on titration using actual oligonucleotide-tagged antibodies. There should be a relationship between the antibody signal detected by flow cytometry and by sequencing, but this relationship is not necessarily one of identity. To maximally benefit from surface phenotype assessment, it is necessary to optimize the antibody panels for the intended purpose to include only target antigens expressed on the interrogated cells, to ensure that the antibodies used actually work, and to find their optimal concentration in actual CITE-Seq experiments.

Here, we tested a pool of 188 Biolegend® TotalSeq™ C antibodies (Table S1) that were oligonucleotide-tagged to be compatible with 10 × Genomics 5′ sequencing. For titration, we used the antibodies at the recommended concentration (1 ×) and generated higher (2 ×) and lower (1/5 ×, 1/25 ×) concentrations. We used human PBMCs, one of the most commonly interrogated cell types in human immune cell studies^17,22–28^. We clustered the five major cell types in PBMCs [CD4 T cells, CD8 T cells, B cells, classical monocytes (CM), and natural killer (NK cells)]. Then, we used their transcriptomes, which are independent of antibody concentration, to map all cells at all concentrations in the same UMAP. After deconvolution, we interrogated and analyzed the signal and background for each antibody at each concentration. We demonstrate which (of the 188 tested) TotalSeq™ antibodies are capable of staining human PBMCs and which concentrations enable sufficient staining quality. Together, these data can be a valuable resource for designing future CITE-Seq experiments on human PBMCs.

## Results

### Identification of major cell types was optimal at the recommended antibody concentration

We first called the five major cell types using the following gating scheme (Fig. S1):

- B cells: CD3^−^CD19^+^
- CD4 T cells: CD3^+^CD4^+^CD8^-^
- CD8 T cells: CD3^+^CD4^−^CD8^+^
- Classical monocytes (CM): CD3^−^CD19^−^CD14^+^CD16^−^
- Natural killer (NK) cells: CD3^−^CD19^−^CD14^−^CD56^+^

Biaxial plots were generated to set the gates. Thresholds to call the major cell types were almost equal for the recommended and double concentrations, but slightly higher for the lower concentrations (0.2 × and 0.04 ×; Fig. S2). At the recommended concentration, 73 B cells (4.9%), 781 CD4 T cells (52.1%), 181 CD8 T cells (12.1%), 39 CM (2.6%), 212 NK cells (14.1%), and 213 other cells (14.2%) were detected. We validated this data by flow cytometry using PBMCs of the same donor and the same gating strategy (Fig. S3A). Frequencies of major cell types detected by flow cytometry and CITE-Seq were similar (Fig. S3B), suggesting high consistency between the two methods. The frequency of remaining cells was similar at 2 × (13.9%). More cells remained unclassified at the lower concentrations (32.7% and 61.7% at 0.2 × and 0.04 ×, respectively; Fig. S4), suggesting that these concentrations were insufficient to saturate many antibodies.

**Figure 1:**
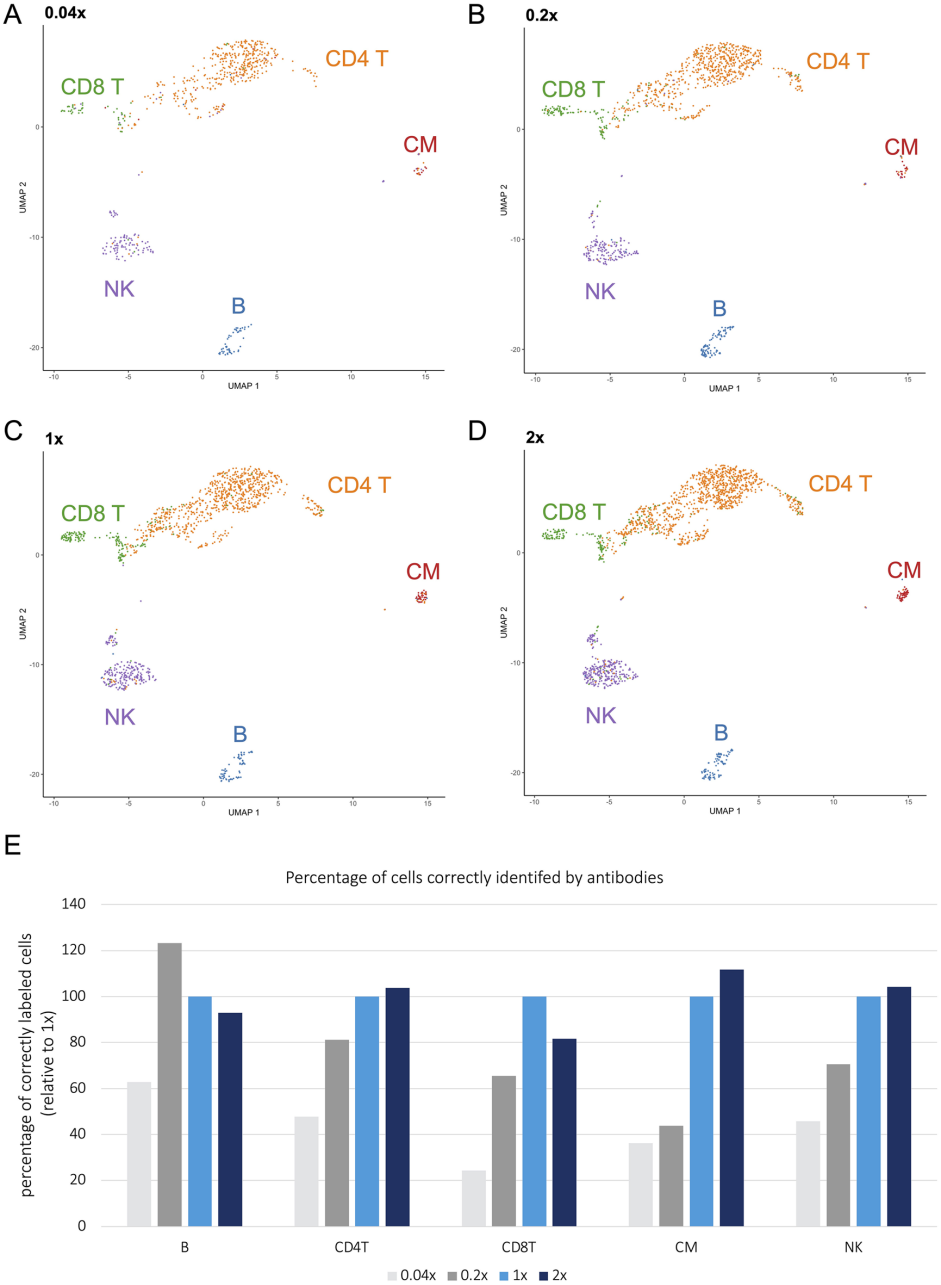
Major cell type identification at different antibody concentrations. (**A**–**D**) Major cell types detected by antibodies at the indicated concentrations were projected on a unified UMAP, in which all cells are clustered by transcriptomes. Major cell type clusters were clearly separated at all concentrations, although antibody dilution reduced the number of identified cells. (**E**) Percentage of correctly identified major cells at different antibody concentrations in relation to 1 ×. Proportions of all major cell types in relation to the total number of cells detected were calculated for each concentration and normalized to the proportions of detected cells at the recommended concentration (1 ×).

We next generated a unified UMAP, in which all cells (stained with any of the four antibody concentrations) were clustered by normalized RNA expression values. This approach was chosen because the RNA signal and pattern is expected to be independent of the antibody concentration used. Clustering by RNA also circumvented poor clustering at low antibody concentrations. The five major cell types detected by antibodies at one of the four concentrations each were then projected on this UMAP (Fig. 1A–D). This approach enabled clear separation of the major cell type clusters at all concentrations, although dilution of the antibody cocktail was associated with a reduction of detectable cell numbers in some clusters. To quantify whether and how antibody concentration affected the accuracy of cell type identification, we calculated the percentage of correctly identified major cell types in relation to 1x, which was set to 100% (Fig. 1E). Doubling the antibody concentration (2 ×) had no relevant impact, and almost all major cell types (on average 99 ± 12% in the five clusters) were called correctly. Lowering the concentration fivefold (0.2 ×) only identified 44% of CM, 65% of CD8 T cells, and 71% of NK cells, whereas detection of CD4 T cells was still acceptable (81%). Reducing the concentration to 0.04 × largely failed to detect the major cell types (24–63%).

### 124 target antigens of 188 tested antibodies were detectable on human PBMCs

**Figure 2:**
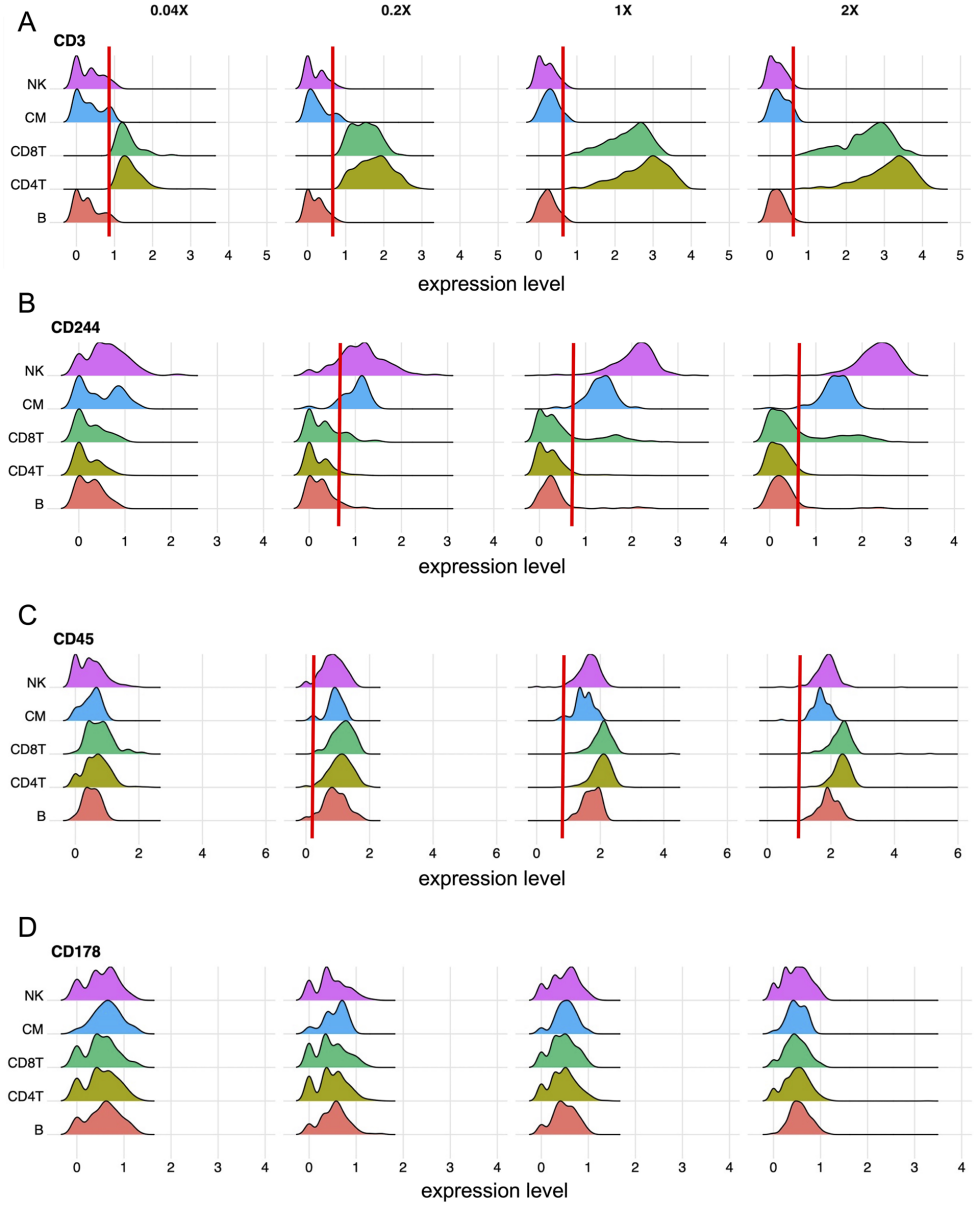
Exemplary ridge plots used to threshold antibodies and to evaluate antigen expression. (**A**) shows an exemplary antigen (CD3) which was detectable at all dilutions. In contrast, CD244 shown in (**B**) was not detected at the lowest concentration (0.04 ×). (**A**, **B**) Antibody thresholds, indicated with red lines, were set to the upper limits of negative populations. (**C**) Some antigens, such as CD45, were detectable in all cells, as expected. Thus, negative populations did not exist. Here, antibody thresholds (red lines) were set to the lower limits of positive populations. If a considerable amount of expression values and the lower limit of the populations were negative, thresholds were not called (as for the 0.04 × concentration in the CD45 example). The exemplary antigen (CD178) shown in (**D**) was not detectable at any concentration. (**A**–**D**) Expression values were centered log-ratio (CLR) normalized.

**Figure 3:**
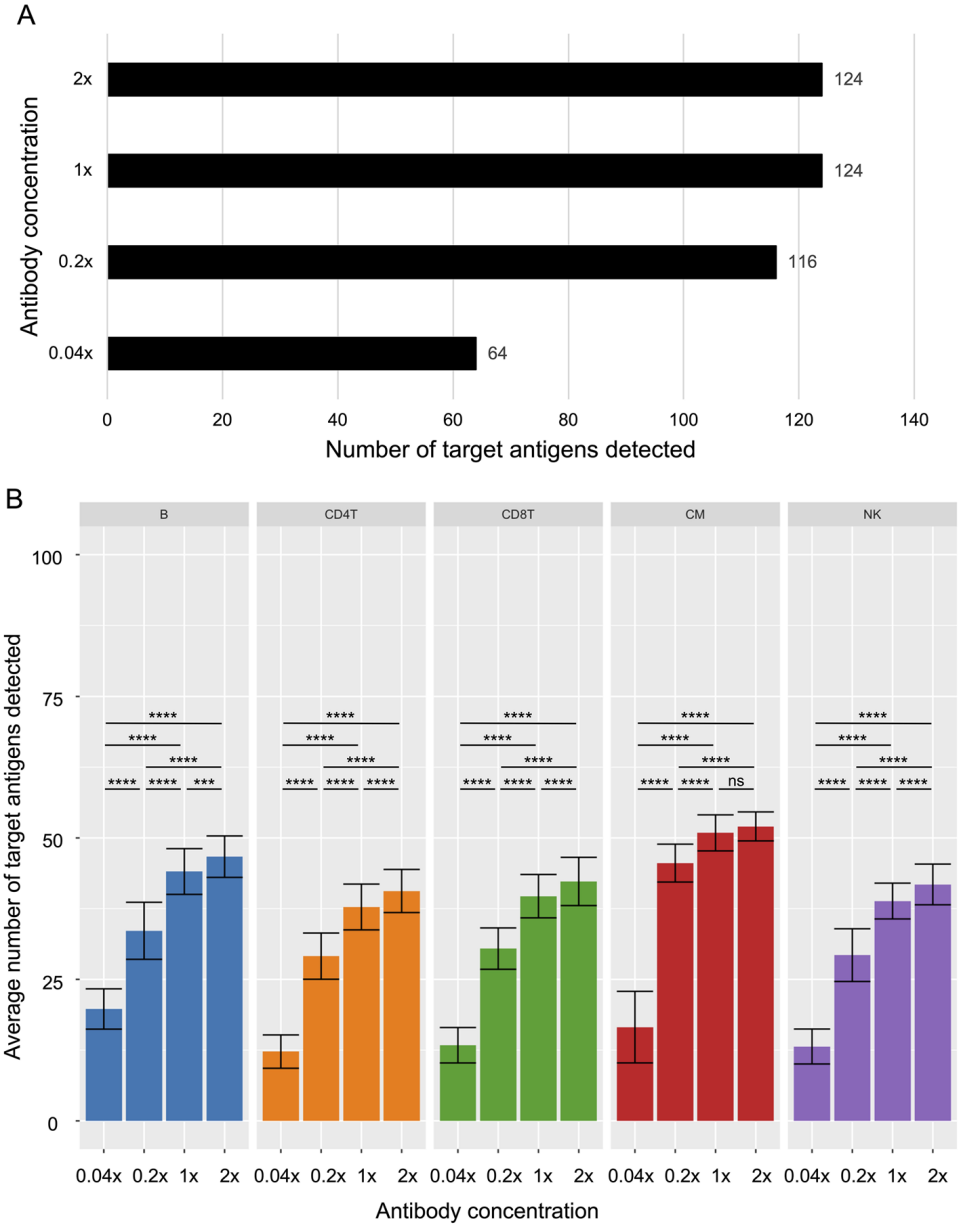
Target antigen expression in major cell types. (**A**) Total number of detectable target antigens at different antibody concentrations. (**B**) Average number of antigens detected at different concentrations in each cell type. N = 52–95 B cells, 422–1005 CD4 T cells, 50–183 CD8 T cells, 16–54 Classical Monocytes (CM), and 110–274 natural killer (NK) cells. Data are expressed as mean ± SD. Statistical significance was determined by one-way ANOVA with Tukey’s multiple comparisons test. *ns* not significant. ***p < 0.001, ****p < 0.0001.

We next generated ridge plots for each individual antibody, which display expression of the target antigen in all the five major cell types at all the four tested antibody concentrations. Ridge plots were used for antibody thresholding and to determine whether and in which cell types the target antigens were detectable at each concentration. Whenever negative populations could be identified, thresholds were set to the upper limits (95th percentile) of the negative populations (Fig. 2A). If negative and positive populations could not be clearly separated, an antigen was determined “not detectable” at the respective concentration. Figure 2B shows the example of CD244, which was only detectable at 2 ×, 1 ×, and 0.2 ×, but not at 0.04 ×. If an antigen was expressed in all cells and negative populations could not be identified, thresholds were set to the lower limit of positive populations (5th percentile, Fig. 2C). Thresholds were manually adjusted to match the upper limits of negative or lower limits of positive populations if automated thresholding to the 95th or 5th percentiles was not accurate. Figure 2D displays the example of an antigen (CD178) that was not detectable at any concentration. Ridge plots of all antibodies with detectable target antigens are shown in PDF S1. Overall, 124 (66%) of 188 tested antibodies were able to detect their target antigens at the recommended concentration. Doubling the concentration did not increase the number of detectable target antigens, whereas a reduction to 0.2 × and 0.04 × decreased it to 116 (61.7%) and 64 (34%), respectively (Fig. 3A). 64 antigens (34%) were not detectable at any antibody concentration. To determine whether any of these antigens were detectable in cells which were excluded after the initial cell gating step, we generated ridge plots for the respective antibodies including such remaining cells (PDF S2). No additional antigens were detectable in the remaining cell cluster. However, cells expressing such antigens might become detectable in specific disease settings, when cells are activated, or when target cells are enriched (for example by cell sorting) prior to analysis.

We determined the number of antigens detected in each individual cell after thresholding and calculated the average number of antigens detectable in each of the major cell types. On average around 30 to 50 antigens were detectable in each cell type at 1 ×. This number was significantly lower at 0.2 × and 0.04 × in all major cell types and significantly higher at 2 × in all cell types but CM (Fig. 3B). Similarly, the total numbers of antibody-derived tags (ADT), which represent all antibody molecules detected in cell-containing droplets before thresholding, distinctly correlated with antibody concentrations (Fig. S5). Of note, ADT counts at 0.04 × were around 0.17 × of the counts at 1 ×. Thus, the drop in ADT count was not linear with the antibody concentration. Counts at 2 × and 0.2 × corresponded better with concentrations and made up around 1.7 × and 0.35 × of the counts at 1 ×, respectively. Mean ADT counts per cell and the standard deviation of ADT counts per cell were significantly higher for antibodies detecting their target antigens compared to antibodies not detecting their target antigens, as expected (Fig. S6).

Of 124 detectable antigens, 16 (12.9%) were detected in all major cell types, whereas 21 (16.9%), 17 (13.7%), and 5 (4%) antigens were exclusively expressed in B cells, CM, and NK cells, respectively. The remaining 65 antigens (52.4%) were expressed in two, three, or four of the major cell types (Fig. S7 and Table S2).

### Most antibodies exhibited a strong dose-response

**Figure 4:**
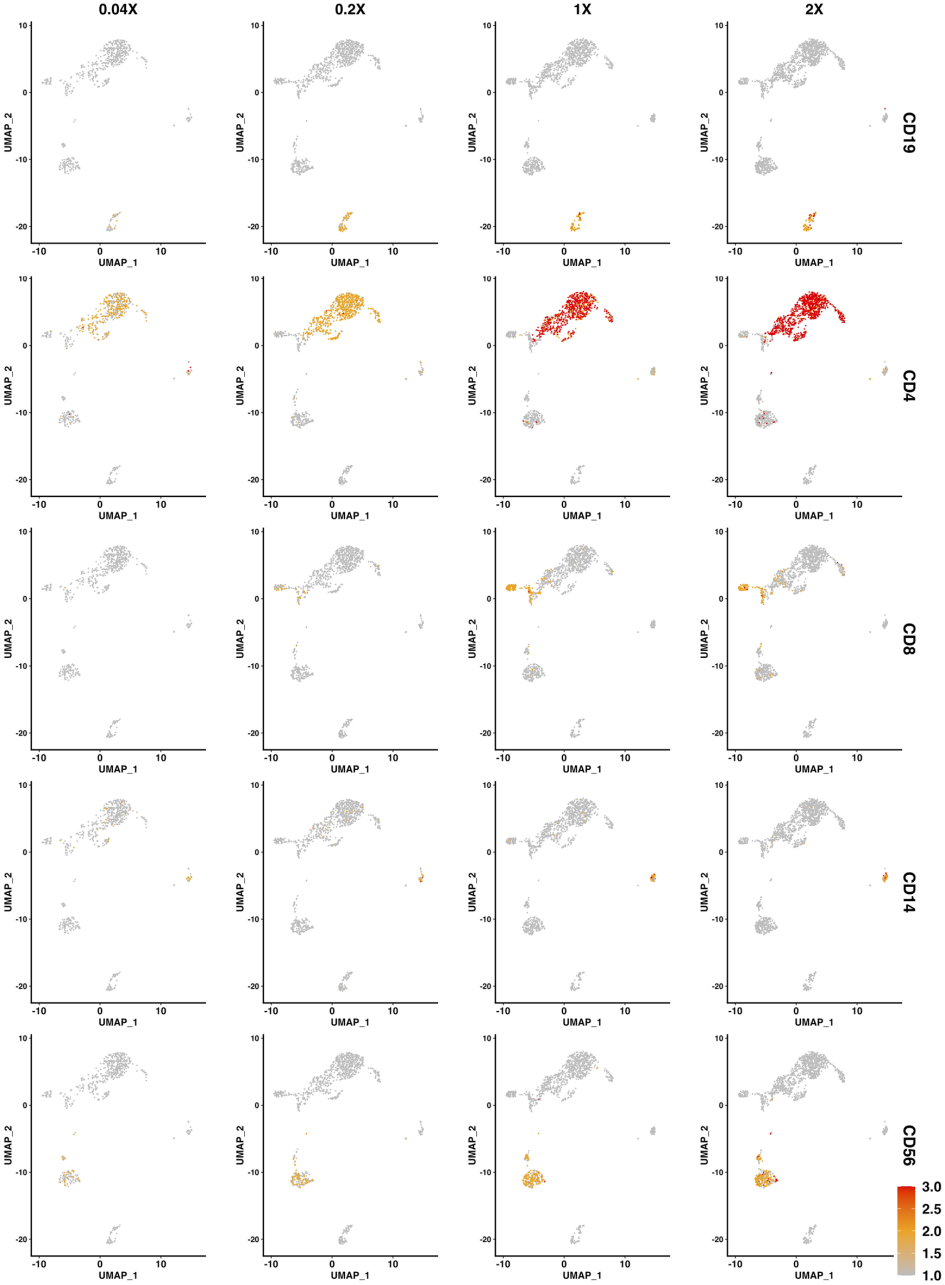
Exemplary feature plots. Expression of exemplary surface antigens (CD19, CD4, CD8, CD14, and CD56) at each of the four concentrations plotted on a unified UMAP, in which all cells, stained with any of the four antibody concentrations, are clustered by transcriptomes. Centered log-ratio (CLR) normalized expression levels in a low dimensional space are indicated by color (scaled between 1 and 3 as indicated by the scale bar; yellow = low to red = high expression).

**Figure 5:**
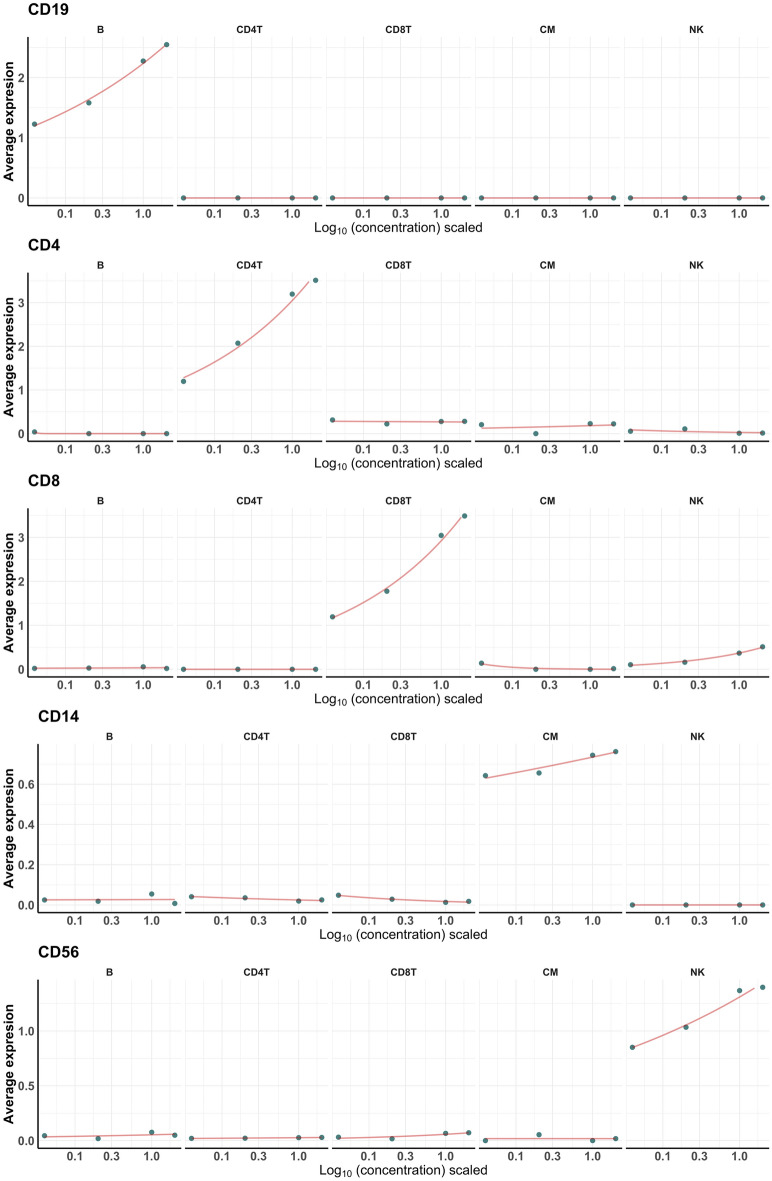
Exemplary dose–response curves. Centered log-ratio (CLR) normalized expression levels of five exemplary surface antigens (CD19, CD4, CD8, CD14, and CD56) at each of the four concentrations in all major cell types. The concentrations (0.04 ×, 0.2 ×, 1 ×, and 2 ×), which were log transformed and scaled by ggplot2’s *scale_x_log10* function (lower to higher concentration), are shown on the x axis. The average expression values for each concentration were fit to a Poisson regression model, a type of a generalized linear model (red lines).

**Figure 6:**
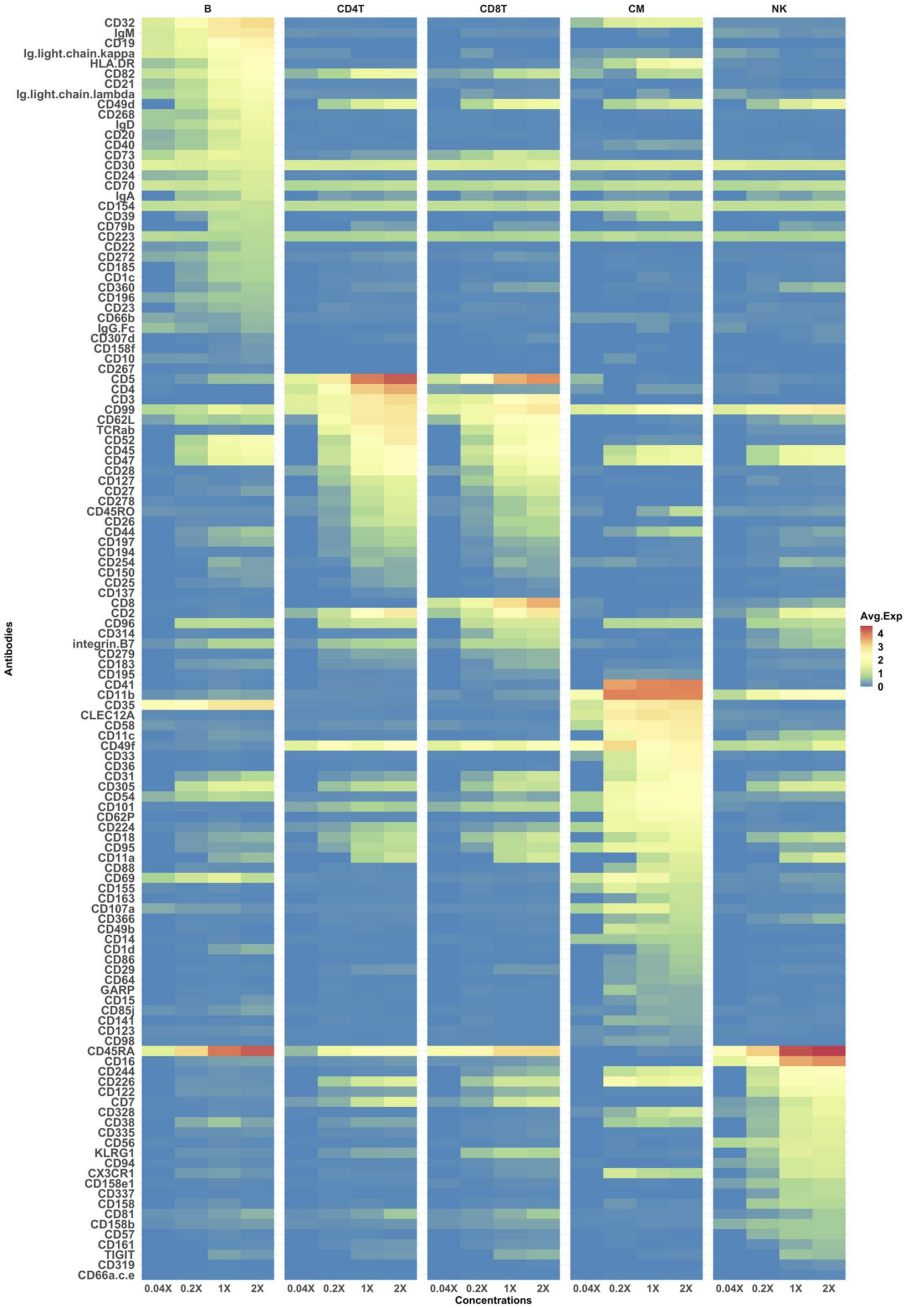
Dose-responses of all thresholded antibodies. Centered log-ratio (CLR) normalized expression levels in all cell types and at all antibody concentrations are displayed as a colored heatmap. Antibodies are clustered depending on the cell type in which the expression was highest (from top to bottom: B cells, CD4 T cells, CD8 T cells, Classical Monocytes (CM), and natural killer (NK) cells).

To visualize dose-responses of all antibodies with detectable antigens, we generated feature plots, in which expression of each individual antigen at different antibody concentrations was projected on the unified UMAP described above (Fig. 4 and PDF S3). Additionally, dose–response curves (Fig. 5 and PDF S4) and a heatmap (Fig. 6) displaying the staining intensity as a function of antibody concentrations in each of the five major cell types were generated. Assessment of these data revealed that the vast majority of antibodies (111 of 124 = 89.5%) showed a considerable dose–response.

**Figure 7:**
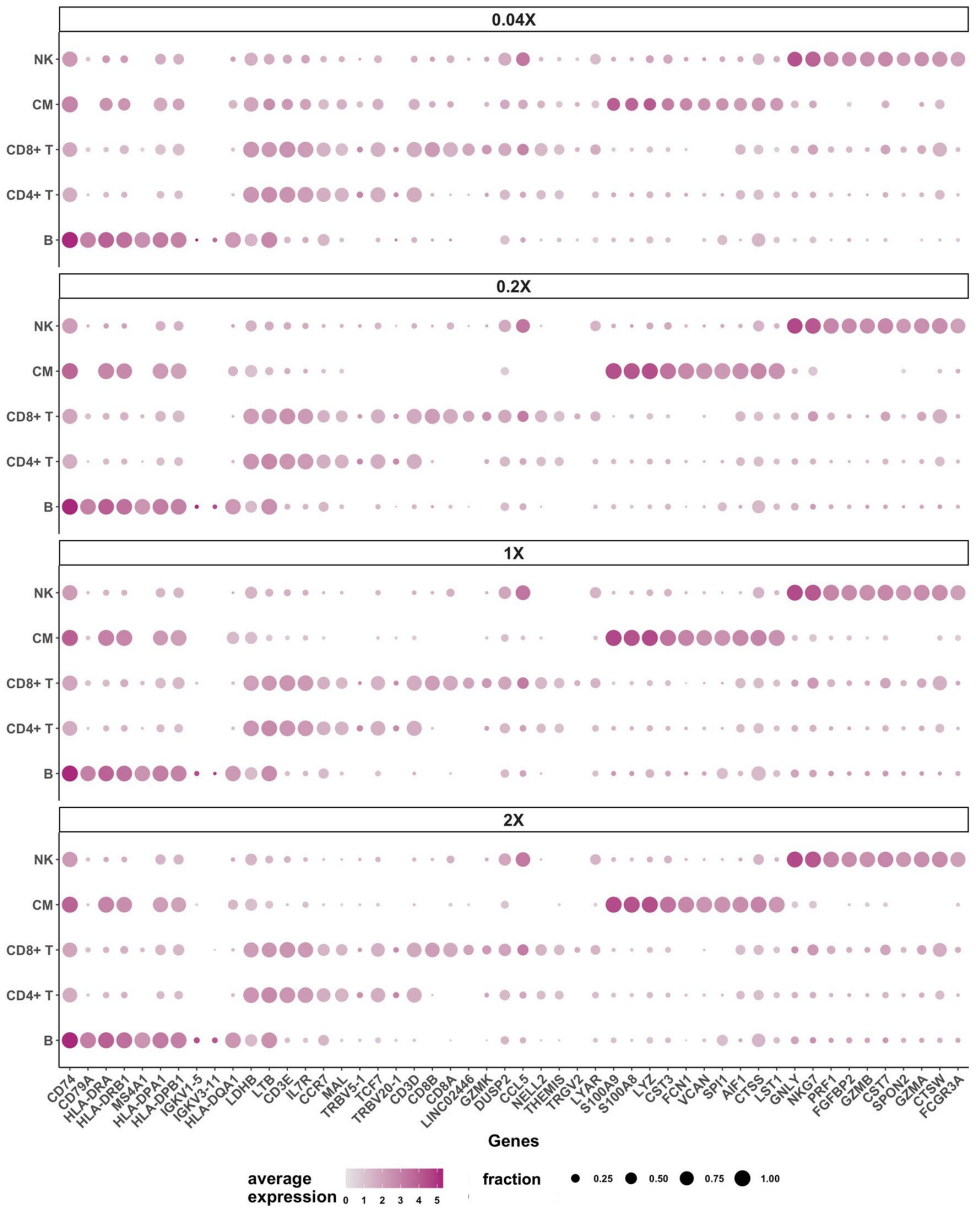
Top 10 genes of major cell types. Average expression values of the 10 most abundant differentially expressed genes (one versus all other comparison) in each of the major cell types at all antibody concentrations are displayed as dot plots. The centered log-ratio (CLR) normalized average gene expression is indicated by color intensity and the fraction of cells expressing an antigen is indicated by the size of the dots. The top 10 B cell antigens are shown on the left, followed by the top 10 CD4 T cell, CD8 T cell, classical monocyte (CM), and natural killer (NK) cell antigens (left to right).

We next generated dot plots displaying expression of the 10 most abundant genes of the five major cell types at all antibody concentrations (Fig. 7). Expression of these genes barely differed between the four concentrations, confirming that clustering by transcriptomes enabled good separation of the major cell types irrespective of antibody dilution. In contrast and in line with the dose–response curves, expression of the 10 most abundant surface antigens detected by antibodies clearly dropped when antibody concentrations were reduced (Fig. S8). A list of all surface antigens and genes with differential expression in the five major cell types is provided in the supplement (Tables S3 and S4).

### Antibody panel optimization could substantially reduce the amount of antibodies needed

**Figure 8:**
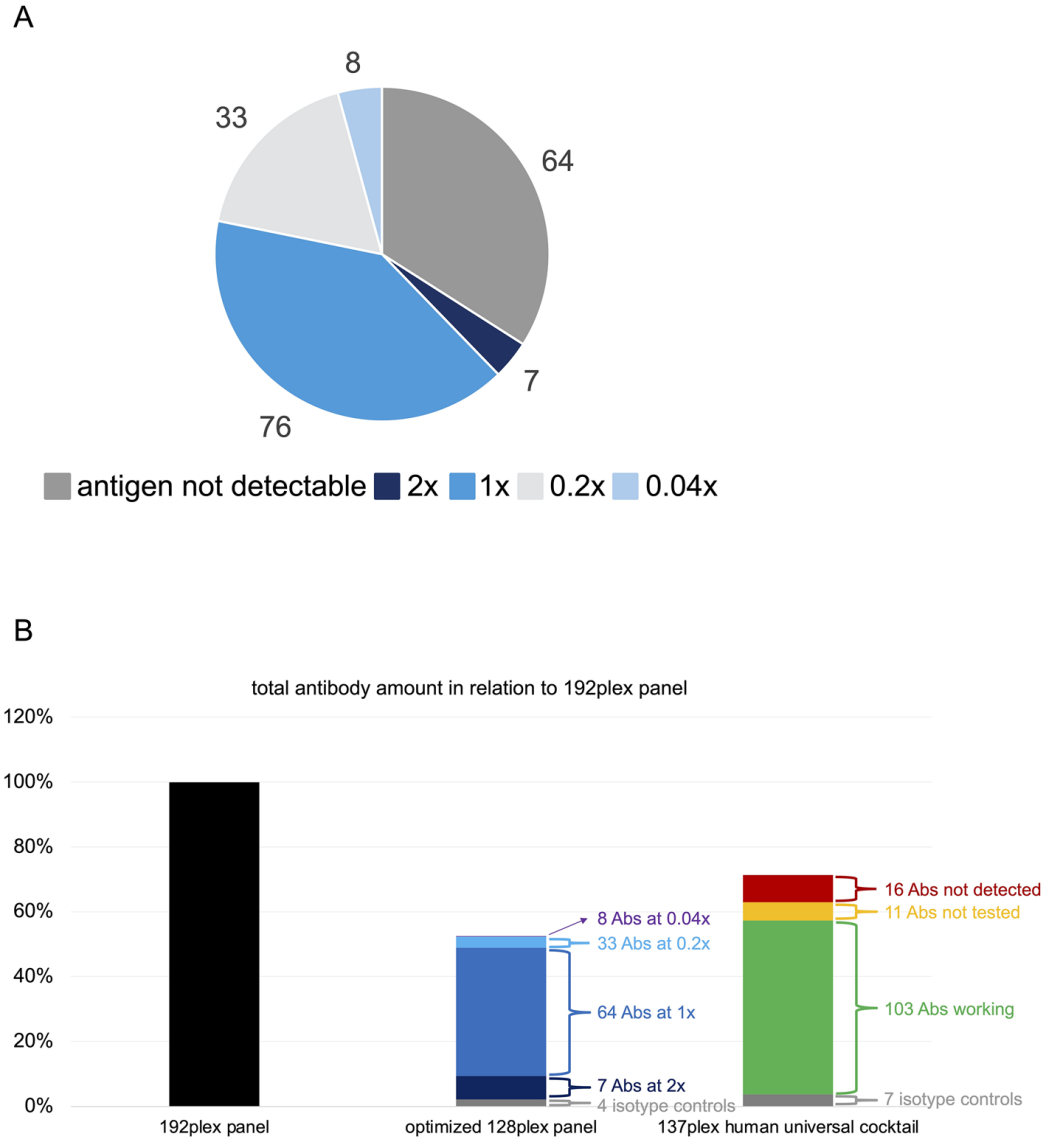
Potential benefits from antibody panel optimization. (**A**) Distribution of optimal antibody concentrations. 64 antibodies showed no signal on PBMCs. The lowest concentration enabling good staining quality was regarded optimal. (**B**) Calculation of the total antibody amount utilized in the optimized 128plex panel (middle) and the commercially available 137plex TotalSeq™ C human universal cocktail (right) in relation to the tested 192plex panel (left). *Abs* antibodies.

We finally aimed to determine the optimal concentration of each individual antibody. Optimal was defined as the lowest concentration enabling sufficient staining quality without loss of information, still achieving complete separation between positive and negative cells. To determine optimal concentrations, ridge plots of all 124 antibodies with detectable antigen expression were carefully reviewed. Overall, 76 antibodies worked best at the recommended concentration. Doubling the concentration was beneficial for 7 antibodies, whereas dilution to 0.2 × and 0.04 × still enabled sufficient staining quality for 33 and 8 antibodies, respectively (Fig. 8A and Table S1). An optimized 128plex panel containing 4 isotype controls and the 124 antibodies able to detect their target antigens on human PBMCs at optimal concentrations, would therefore provide the same information as the tested 192plex panel. We next estimated the total amount of antibodies that could potentially be saved by using such an optimized antibody panel. To this end, we summed up the relative amounts of each antibody used at an optimal concentration in relation to the tested 192-plex panel and divided this by 192: (7 × 2 + 80 × 1 + 33 × 0.2 + 8 × 0.04 + 64 × 0)/192 = 0.53. Panel optimization could thus reduce the total antibody amount needed by 47%. We also compared our optimized panel with the commercially available 137plex TotalSeq™ C panel, which includes 130 antibodies and 7 isotype controls. 103 of the 130 antibodies were able to detect their target antigens in our study, 16 antibodies were not, and 11 antibodies were not included in our 192plex panel (Fig. 8B). Thus, our optimized panel includes at least 10 more antibodies able to detect their target antigens on human PBMCs (assuming that the remaining 11 antibodies included in the 137plex panel but not tested in our study would all work), although the total antibody amount needed is still 26% less.

## Discussion

In this study, we titrated 188 CITE-Seq antibodies on human PBMCs to establish an optimized antibody panel for future utilization in this cell population. By combining cell surface with transcriptome analysis and thus overcoming the issue of poor correlation between mRNA and protein surface expression in immune cells^17–19^, CITE-Seq enables more precise phenotyping of immune cells than scRNA-Seq alone. Consequently, application of this technology in immunology is expected to rapidly expand within the next few years. Given that a high number of antibodies used in cytometry do not work as intended and concentrations recommended by the vendors are not always optimal, antibody validation and titration in the sample of interest is essential^29^. Determination of optimal staining concentrations improves accuracy, reduces unwanted background signals due to non-specific binding, and can save costs. Whereas antibody titration in flow cytometry is straightforward and relatively well established^30,31^, there is sparse data on titration of CITE-Seq antibodies. CITE-Seq facilitates the use of large antibody panels and antibody staining intensity strongly influences sequencing costs. From an economic perspective it is thus highly relevant to determine optimal dilutions, which can be defined as the lowest concentrations enabling sufficient staining quality.

Human PBMCs are particularly suitable for application of CITE-Seq, since they offer several advantages over tissue-derived leukocytes^32^. Such favorable characteristics include (1) easy accessibility without any need for surgical interventions, (2) no need for enzymatic digestion or mechanical separation, (3) availability from patients in many large cohort studies, (4) ready availability from healthy individuals that can be used as controls, (5) long-term stability on liquid nitrogen, and (6) a high potential to be used as biomarkers in clinical practice. PBMCs collected in clinical trials allow correlating experimental findings with clinical outcome data and to thus achieve high translational potential.

Considering that CITE-Seq of human PBMCs represents a powerful technology to study immune responses in health and disease, an antibody panel that enables optimal staining quality at the lowest possible cost can be an important resource for future research. Here, we reveal that about one third of 188 tested CITE-Seq antibodies were not able to detect their target antigens. In these cases, it remains unknown whether the target antigens were not expressed in any PBMCs or whether some of these antibodies were technical failures (antibody not working). About one third of the remaining antibodies could be used at lower than recommended concentrations without loss of staining quality. Performance of only a few antibodies improved after doubling the concentration. Utilization of an optimized antibody panel only including the 124 working antibodies at optimal concentrations and isotype controls would reduce the total antibody amount needed by almost 50%. Notably, this reduction comes without compromising staining quality; our proposed strategy actually improves the performance of the panel. The manufacturer could use this data to optimize their universal antibody cocktail for human PBMCs. Additionally, our findings can help to optimize custom panels designed by customers, although the actual cost savings would depend on the individual composition of the panel.

Titration of a similarly sized CITE-Seq antibody panel has not yet been performed. In a recent study, Buus and colleagues investigated how antibody concentration (1 × vs 0.25 × dilution), staining volume (50 µl vs. 25 µl), cell count (1 × 10^6^ vs. 0.2 × 10^4^), and tissue type (PBMCs vs. immune cells from human lung tumor tissue) affected performance of a 52plex CITE-Seq panel^33^. Consistent with our data, they revealed that dilution of many antibodies did not impair their performance. Reducing staining volume only affected antibodies used at low concentrations and targeting highly expressed antigens, since the number of antigens present in the sample might then exceed the total number of antibody molecules (loss of saturation). Consequently, this effect was counteracted by reducing the cell count. In contrast to our study, they did not use a lyophilized antibody cocktail but titrated each antibody individually using starting concentrations (1 ×) which were based on previous experience, epitope abundance or vendor recommendation and a fourfold dilution (0.25 ×) of these concentrations. They also established and validated an adjusted antibody panel which yielded higher signal-to-noise ratio and was substantially cheaper compared to the 1 × concentration. Titrating antibodies individually based on a-priori information facilitates a more targeted determination of optimal dilutions and offers the opportunity to directly validate the adjusted panel (as was done by Buus and colleagues).

For practical reasons of relevance to daily lab operation, we titrated a lyophilized TotalSeq™ C antibody cocktail rather than individual antibodies. (1) Individual titrations of large antibody panels by future customers are infeasible due to high experimental and labor costs. (2) We assumed that titrating the whole panel based on the recommended concentrations would be most useful for enabling adjustments of concentrations.

We acknowledge several limitations of our study: (1) Our findings only apply to human PBMCs, and (2) our data does not allow to differentiate whether an antibody was not working as intended or whether its target antigen was just not expressed in the major cell types present in PBMCs (NK cells, CM, CD8 T cells, CD4 T cells, and B cells). Regardless of this, antibodies not able to detect their antigens in our study can be discarded from a panel specifically designed for application in PBMCs, unless analysis of very rare cell populations after enrichment by sorting is intended. Utilization of CITE-Seq antibodies in other tissues or such rare cell types would require validation and titration in these particular cell populations. (3) We only used one donor. This is common practice in titration studies, because multiple samples are associated with higher experimental costs. Finally, (4) some antigens that were not detectable in our sample could potentially be expressed by PBMCs in the setting of diseases or after stimulation (e.g. with endotoxins or superantigens such as LPS or SEB).

In conclusion, this resource for building an informative and cost-effective panel of TotalSeq™ C antibodies and use them at their optimal concentrations improves future CITE-seq experiments on human PBMCs. This panel includes 4 isotype controls and 124 of 188 tested antibodies, of which 76 are used at the recommended concentration, 41 are used at reduced concentrations, and 7 are used at the double concentration. This optimized panel utilizes a substantially lower amount of antibodies while increasing overall staining quality compared to currently available antibody panels.

## Methods

### Ethical statement

This study was approved by the Institutional Review Board at the La Jolla Institute for Immunology (LJI) and all experiments were performed in accordance with the guidelines of this committee and the Declaration of Helsinki. Written informed consent was obtained from all participants.

### Human subject

A blood sample from one healthy adult donor was collected through the La Jolla Institute for Immunology’s in-house Normal Blood Donor Program (NBDP). All participants of the NBDP must not have a history of any chronic infectious disease, cardiovascular, kidney or liver disease, cancer, autoimmune disease, chronic anemia, bleeding disorder, recent surgery or organ/tissue transplantation, must not be pregnant/breast-feeding or weigh below 50 kg, and must not take any cardiac or anticoagulant/antiplatelet drugs. Absence of HIV, Hepatitis B, and Hepatitis C was confirmed by diagnostic blood tests.

### Isolation of PBMC samples

Venous blood samples were collected into heparin-coated tubes and centrifuged at 400×*g* for 10 min at RT to remove platelet rich plasma. PBMCs were isolated by Ficoll Paque (Sigma Aldrich) density-gradient centrifugation and resuspended in CryoStor® CS10 (Stemcell), a serum-free and animal component-free cryopreservation medium containing 10% DMSO. PBMC-containing vials were cooled down in Mr. Frosty™ Freezing Containers (ThermoFisher Sientific™) and subsequently cryopreserved on liquid nitrogen until used.

### Antibody-staining of PBMCs and library preparation

Prior to the experiment, PBMC-containing tubes were filled with RPMI-1640 solution supplemented with 10% fetal bovine serum (FBS) and thawed in a 37 °C water bath. Cells were counted and centrifuged at 400 rcf for 5 min at room temperature (RT). Subsequently, the pellets were resuspended in 1 ml phosphate-buffered saline (PBS) with 0.04% w/v bovine serum albumin (BSA) and centrifuged at 400 rcf for 5 min. Pellets were again resuspended in 1 × PBS with 0.04% w/v BSA to achieve a target concentration of 1000 cells/µl. Cells were counted again and volume was adjusted to obtain the target cell concentration. 10^6^ cells (1 ml) each were transferred to four different microcentrifuge tubes, centrifuged at 400 rcf for 5 min at 4 °C, resuspended with 50 µl chilled PBS + 1% w/v BSA, and then incubated with 5 µl Fc receptor blocking solution (BioLegend® Human TruStain FcX™) at 4 °C for 10 min. The 192plex antibody mix (including 188 antibodies and 4 isotype controls) was prepared to achieve the concentration recommended by the manufacturer (1 ×), two times the recommended concentration (2 ×), and one fifth (0.2 ×) as well as one twenty-fifth (0.04x) of the recommended concentration in the same total staining volume (10 µl). Each of the four antibody mixes (2 ×, 1 ×, 0.2 ×, and 0.04 ×) was added to one of the four tubes, which were filled up with PBS + 1% w/v BSA to a total volume of 100 µl, incubated for 30 min at 4 °C and washed in PBS + 0.04% w/v BSA. Cells stained with any of the four antibody concentrations (one million cells each) were then hashtagged by incubation in 100 µl Cell Multiplexing Oligo (10 × Genomics) for 5 min at RT and washed three times with PBS + 1% BSA at 4 °C (total volume 2 ml). Cells were counted again, volume adjusted to obtain the target cell concentration (1000 cells/µl) and pooled in one tube. Finally, 16.5 µl of the cell suspension were incubated with the 10 × Genomics Master Mix for a targeted cell recovery of 10,000 cells, loaded onto a Chromium Next GEM Chip (10 × Genomics), and further steps of library preparation were performed according to the Chromium Next GEM Single Cell 5’ Protocol.

### Post-sequencing quality control and data pre-processing

We used Cell Ranger (www.10xgenomics.com) for demultiplexing, which yielded a median number of 8116 (~ 2000 cells per sample). All cells with transcriptomes were retrieved. We subsequently performed low viability (mitochondrial gene content > 10%), low staining quality (antibodies detected per cell < 200 or > 100,000), doublet (maximum hashtag oligonucleotide (HTO) count/mean HTO count > 3 and DoubletFinder v3^34^), and negative HTO (HTO count ≤ 20) removal, after which 6640 cells remained. A median of 1670 genes were detected per cell based on UMI (unique molecular identifier) counts. The transcriptome data was log normalized and the antibody data was CLR (centered log-ratio) normalized. The margin was set to 2 (column-wise) to normalize for differences in ADT depth across cells. Prior to calling thresholds for each antibody, biaxial plots were used to gate the major cell populations: B cells, CD4 T cells, CD8 T cells, natural killer (NK) cells, and classical monocytes (CM).

### Antibody thresholding

Antibody thresholding is critical to eliminate background noise due to encapsulation of unbound antibodies during droplet generation and unspecific binding^35^. Antibody thresholds for CD4, CD8, CD3, CD19, CD14 and CD56 were obtained by determining the background signal of each antibody in a negative cell population. Considering that the antibody concentration might impact the signal-to-noise ratio, the thresholding was done separately for each concentration (2 ×, 1 ×, 0.2 ×, and 0.04 ×, Fig. S2). Remaining cells (not identified as NK, CM, CD8 T, CD4 T or B cells), were excluded from further analyses. Figure S4 summarizes the removal of cells during quality control and cell type calling. Next, ridge plots were used to set the thresholds for each of the other antibodies at each concentration (2 ×, 1 ×, 0.2 ×, and 0.04 ×).

### scRNA-seq analysis

Seurat v4.0.6^36^ was utilized to identify differentially expressed genes. Differentially expressed genes were identified using Seurat's *FindAllMarkers* function which by default uses the non-parametric Wilcoxon rank-sum test. Significant genes were selected based on an adjusted P value (Bonferroni corrected) threshold of < 0.05 and no avg_log2FC threshold. Differences in the number of antigens detected at the four concentrations (Fig. 3B) were statistically evaluated using one-way analysis of variance (ANOVA) with a post hoc Tukey’s test performed in GraphPad Prism 9.3.1 (GraphPad Software, San Diego, CA, USA). Mann–Whitney *U* test (GraphPad Prism 9.3.1) was used to determine statistical significance of differences in mean ADT counts per cell and standard deviation of ADT counts per cell between antibodies detecting and antibodies not detecting their targent antigens. P values < 0.05 were considered statistically significant. Ggplot2^37^ was used to generate the heatmap, bar plots, ridge plots and dose response curves. Dot plots showing the top 10 differentially expressed genes and antibodies for each cell population were generated using the dittoSeq R package^38^. The Venn diagram was calculated using the Bioinformatics & Evolutionary Genomics online tool (van de Peer lab, VIB-Ugent Center for Plant Systems Biology, Ghent University; http://bioinformatics.psb.ugent.be/webtools/Venn/).

### Clustering

We used the UMAP (Uniform Manifold Approximation and Projection) dimensionality reduction algorithm to project the identified cell populations for the samples in a 2D space. The first 25 principal components from PCA (principal component analysis) were used to run the UMAP algorithm. To cluster the data, we used Seurat’s default Louvain clustering algorithm with the default resolution parameter and random seed set to 42 to ensure reproducibility. Cells were clustered by the log normalized transcriptome data.

### Flow cytometry

PBMCs were thawed in a 37 °C water bath and washed with cold FACS buffer (PBS w/o Ca/Mg, 2% FBS). Viability and cell concentration were determined by trypan blue dye exclusion using a hemocytometer. About 1.5 million cells were resuspended in a staining master mix containing anti-human Fc-Block (Biolegend™, San Diego, CA, USA), fixable viability dye (Tonbo Biosciences, San Diego, CA, USA) and fluorochrome-coupled antibodies (Biolegend™, San Diego, CA, USA) against the indicated antigens. Viability dye was used at 1:1000 dilution, Fc-block at 1:75 and antibodies were used at a final dilution of 1:100. Cells were stained for 30 min on ice. Stained cells were washed with cold FACS buffer and fixed in 100 µl eBioscience™ IC Fixation Buffer (Invitrogen™, ThermoFisher Scientific, Waltham, MA, USA) for 10 min at RT. Single color-stained beads (UltraComp eBeads™, Invitrogen) were used for compensation control. Data was acquired on a BD LSR II flow cytometer (BD® Biosciences, Franklin Lakes, NJ, USA) and analyzed with FlowJo software (FlowJo LLC, Ashland, OR, USA).

## Supplementary Information

Supplementary Information 1.

Supplementary Information 2.

Supplementary Information 3.

Supplementary Information 4.

Supplementary Information 5.

Supplementary Information 6.

Supplementary Information 7.

Supplementary Information 8.

Supplementary Information 9.

## Data Availability

The datasets generated and analyzed during the current study are available in the National Center for Biotechnology Information (NCBI) Gene Expression Omnibus (GEO) repository, [Accession Number: GSE213282].
