# Supplementary material for: Titration of 124 antibodies using CITE-Seq on human PBMCs
Source: Sci Rep. 2022 Dec 2;12:20817. doi: 10.1038/s41598-022-24371-7 (PMC9718773; doi:10.1038/s41598-022-24371-7)
Supplement: Supplementary file 1 — Supplementary Information 1. [file 41598_2022_24371_MOESM1_ESM.docx]

Supplementary Material

Titration of 124 antibodies using CITE-Seq on human PBMCs

Felix Sebastian Nettersheim^1,2^, Sujit Silas Armstrong^1^, Christopher Durant^1^, Rafael Blanco-Dominguez^1,3^, Payel Roy^1^, Marco Orecchioni^1^, Vasantika Suryawanshi^1^, Klaus Ley^1,4,5^*

*^1^La Jolla Institute for Immunology, La Jolla, 92037, CA, USA*

*^2^Department of Cardiology, Faculty of Medicine and University Hospital Cologne, University of Cologne, Cologne, 50937, Germany*

*^3^Centro Nacional de Investigaciones Cardiovasculares, Madrid, 28029, Spain*

*^4^Department of Bioengineering, University of California, San Diego, San Diego, 92093, CA, USA*

*^5^Immunology Center of Georgia (IMMCG), Augusta University, Augusta, 30912, GA, USA*

***Corresponding author:** klaus@lji.org

**Supplementary Figures**

**Figure S1.** Gating scheme for identification of the five major cell types. B cells were identified as CD3^-^CD19^+^. CD4 T cells and CD8 T cells were gated as CD3^+^CD4^+^CD8^-^ and CD3^+^CD4^-^CD8^+^, respectively. Classical monocytes (CM) were identified as CD3^-^CD19^-^CD14^+^CD16^-^ and natural killer (NK) cells were defined as CD3^-^CD19^-^CD14^-^ CD56^+^.

**
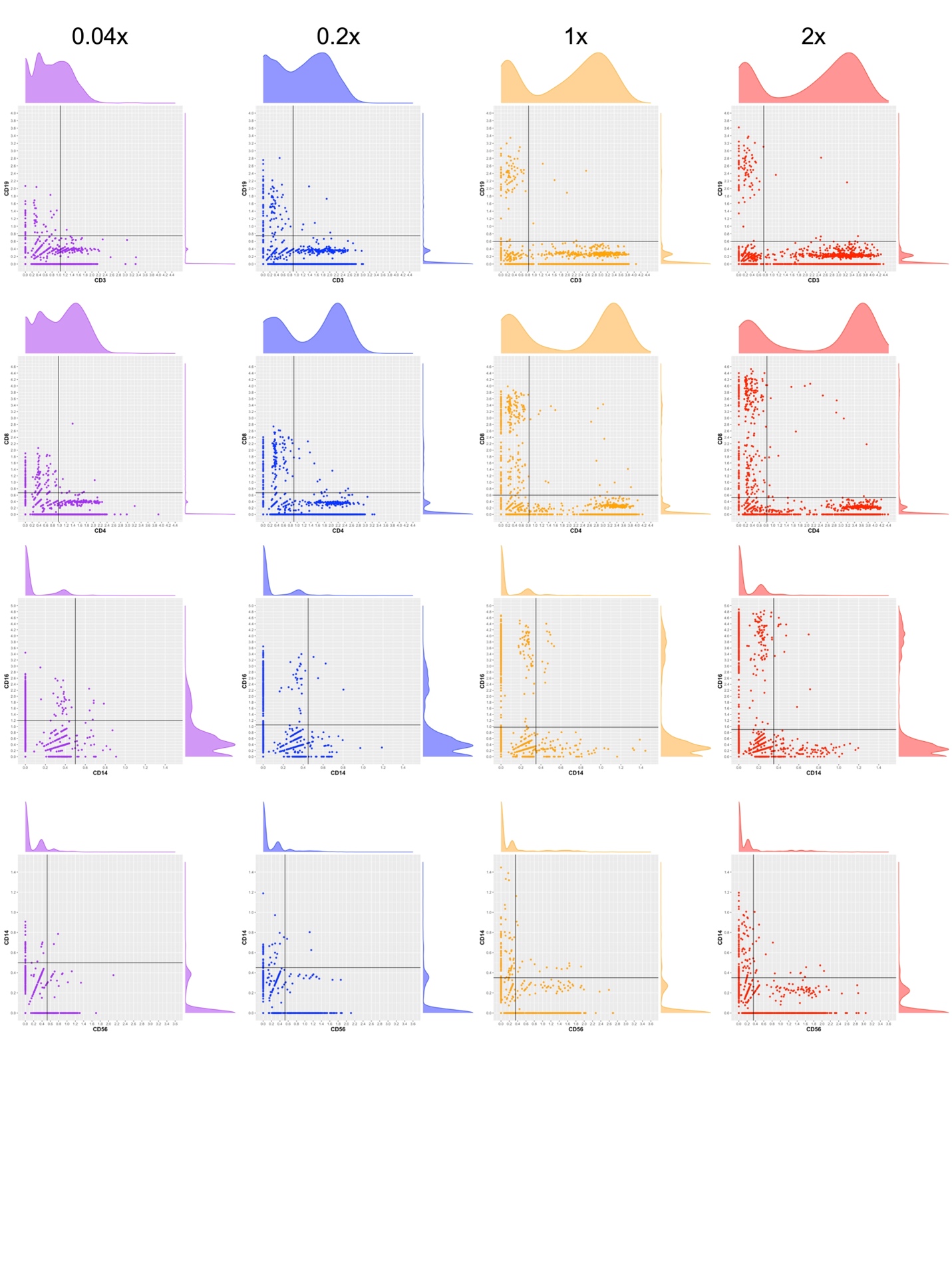
Figure S2.** Biaxial plots to call major cell types at each antibody concentration. Black lines indicate the thresholds which were used for gating. Centered-log ratio (CLR) normalized antibody expression values were used to generate the plots. The distributions of cells are additionally visualized by density plots along the x and y axis.

**Figure S3.** Validation of major cell type calling by flow cytometry. **(A)** PBMCs of the same healthy donor used for the sequencing experiment were analyzed by flow cytometry. Major cell types were called using the same gating scheme as in the CITE-Seq experiment. **(B)** Frequencies of major cell types and remaining cells as detected by flow cytometry (FACS) and CITE-seq.

**Figure S4.** Quality control. Criteria for removal of cells during each quality control (QC) step as well as subsequent calling of major cell types, cells removed, and total cells left after each QC step. HTO = hashtag oligonucleotide.

**Figure S5.** Total counts of antibody-derived tags (ADT) in all cells. ADT counts represent the total number of antibody molecules detected in cell-containing droplets before thresholding.

**Figure S6.** Mean ADT count per cell and standard deviation of ADT counts per cell for antibodies (Abs) detecting and antibodies not detecting their target antigen. ADT counts represent the total number of antibody molecules detected in cell-containing droplets before thresholding. Statistical significance was determined by Mann-Whitney U test. **** p < 0.0001.


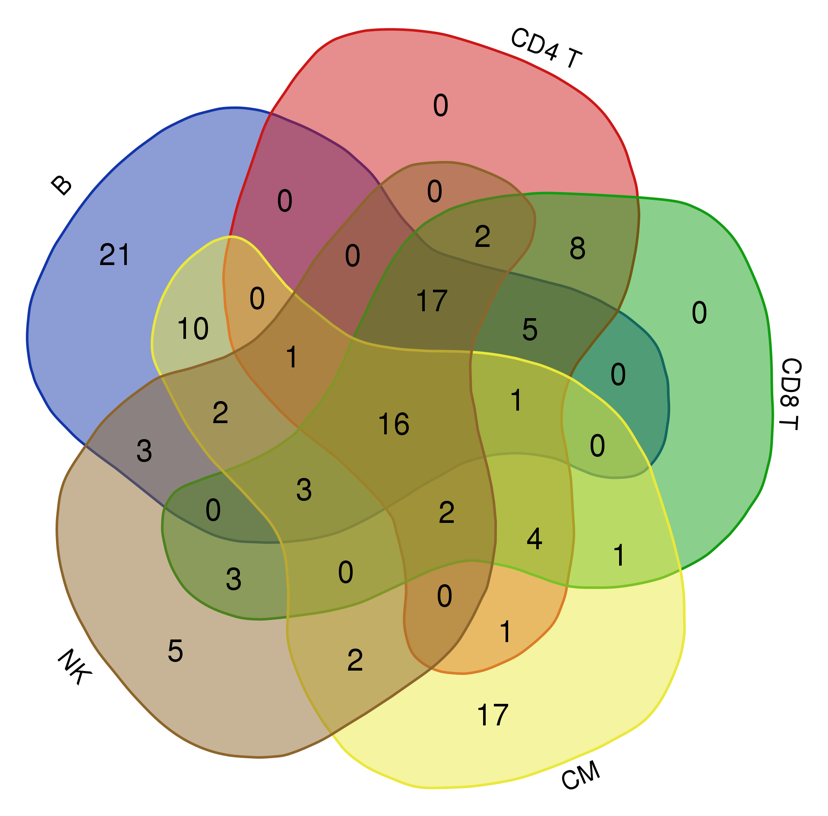


**Figure S7.** Venn-diagram displaying the expression pattern of surface antigens in the five major cell types. 21, 8, 17, and 5 antigens were exclusively expressed in B cells, T cells, classical monocytes (CM), and natural killer (NK) cells, respectively. Overall, 28, 13, 24, and 16 antigens were expressed in two, three, four, and all cell types, respectively.

**Figure S8.** Top 10 surface antigens of all major cell types. Average expression values of the 10 most abundant differentially expressed surface antigens in each of the major cell types at all four antibody concentrations are displayed as dot plots. The centered log-ratio (CLR) normalized average antigen expression is indicated by color intensity and the fraction of cells expressing an antigen is indicated by the size of the dots. The top 10 B cell antigens are shown on the left, followed by the top 10 CD4 T cell, CD8 T cell, classical monocyte (CM), and natural killer (NK) cell antigens (left to right).

**Supplementary PDFs**

**PDF S1.** Ridge plots of all 124 antibodies with detectable target antigen expression. Antibody thresholds, indicated with red lines, were set to the upper limits (95% percentile) of negative populations. If target antigens were detectable in all cells and negative populations did not exist, antibody thresholds (red lines) were set to the lower limits (5% percentile) of positive populations. Thresholds were manually adjusted to match the upper limits of negative or lower limits of positive populations if automated thresholding to the 95^th^ or 5^th^ percentiles was not accurate. Expression values shown on the x axis were centered log-ratio (CLR) normalized.

**PDF S2.** Ridge plots of all 64 antibodies with undetectable target antigen expression including a cluster of the remaining cells which were excluded during gating of major cell types. None of the target antigens were detectable in the remaining cell cluster. Expression values shown on the x axis were centered log-ratio (CLR) normalized.

**PDF S3.** Feature plots of all 124 antibodies with detectable target antigen expression. Expression of surface antigens at each of the four concentrations plotted on a unified UMAP, in which all cells, stained with any of the four antibody concentrations, are clustered by transcriptomes. Centered log-ratio (CLR) normalized expression levels in a low dimensional space are indicated by color (scaled between 1 and 3 as indicated by the scale bar on the first page; yellow = low to red = high expression).

**PDF S4.** Dose-response curves of all 124 antibodies with detectable target antigen expression. Centered log-ratio (CLR) normalized expression levels of surface antigens at each of the four concentrations in all major cell types. The concentrations (0.04x, 0.2x, 1x, and 2x), which were log transformed and scaled by ggplot2’s *scale_x_log10* function (lower to higher concentration), are shown on the x axis. The average expression values for each concentration were fit to a Poisson regression model, a type of a generalized linear model (red lines).

**Supplementary Excel Tables**

**Table S1.** Antibody list. Column A: target antigens of all antibodies included in the tested 192plex panel. Column B: Optimal concentrations of antibodies in relation to the recommended concentration (2x, 1x, 0.2x, and 0.04x, or target antigen not detectable). Column C: Tested antibodies which were included in the commercially available 137plex human universal cocktail marked with an x. Columns E and F: Antibodies (E) contained in the optimized 128plex panel and their concentrations (F).

**Table S2.** Target antigen expression in the five major cell types. Column A: Target antigens which were detectable in any cell type. Columns B-F: Antigen expression in the respective cell types (B cells, C4 T cells, CD8 T cells, Classical Monocytes (CM), and natural killer (NK) cells) is marked with an x.

**Table S3.** Differentially expressed surface proteins. The table contains one sheet for each of the four concentrations (0.04x, 0.2x, 1x, and 2x). Column A: surface proteins (that is target antigens of TotalSeq antibodies), ordered by the cell type in which they were differentially expressed. Surface protein expression was statistically compared between cell types within each concentration. Column B: P value. Column C: average log2 fold change. Columns D and E: principal components 1 (D) and 2 (E). Column D: adjusted P value. Column E: cell type, in which the antigen was differentially expressed (compared to all other cell types).

**Table S4.** Differentially expressed genes. The table contains one sheet for each of the four concentrations (0.04x, 0.2x, 1x, and 2x). Gene expression was statistically compared between cell types within each concentration. Column A: antigens, ordered by the cell type in which they were differentially expressed. Column B: P value. Column C: average log2 fold change. Columns D and E: principal components 1 (D) and 2 (E). Column D: adjusted P value. Column E: cell type, in which the antigen was differentially expressed (compared to all other cell types).
