# Supplementary material for: Titration of 124 antibodies using CITE-Seq on human PBMCs
Source: Sci Rep. 2022 Dec 2;12:20817. doi: 10.1038/s41598-022-24371-7 (PMC9718773; doi:10.1038/s41598-022-24371-7)
Supplement: Supplementary file 2 — Supplementary Information 2. [file 41598_2022_24371_MOESM2_ESM.pdf]

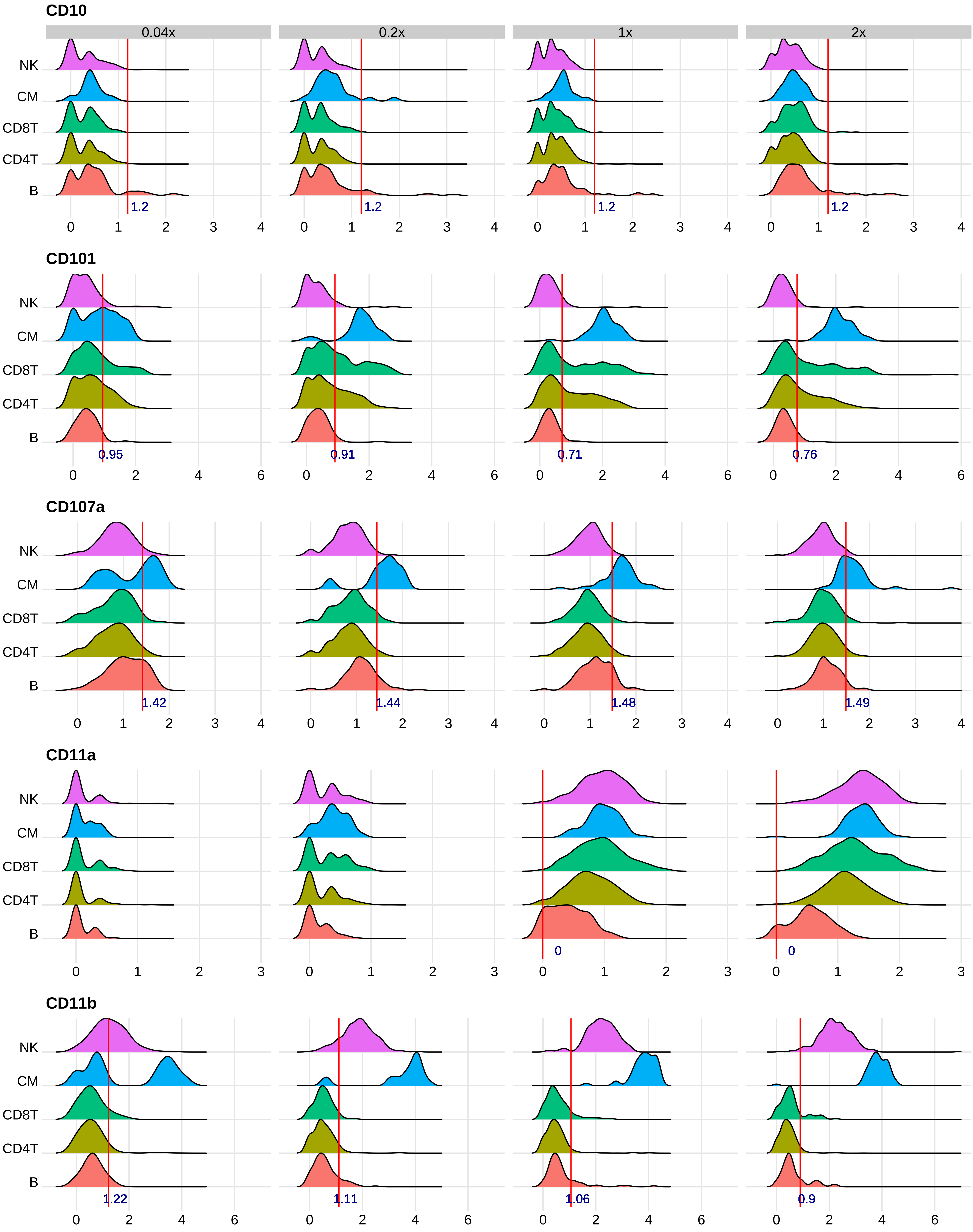
CD10
0.04x
0.2x
1x
2x
NK
CM
CD8T
CD4T
B
1.2
1.2
1.2
1.2
CD101
0.95
0.91
0.71
0.76
CD107a
1.42
1.44
1.48
1.49
CD11a
0
0
CD11b
1.22
1.11
1.06
0.9

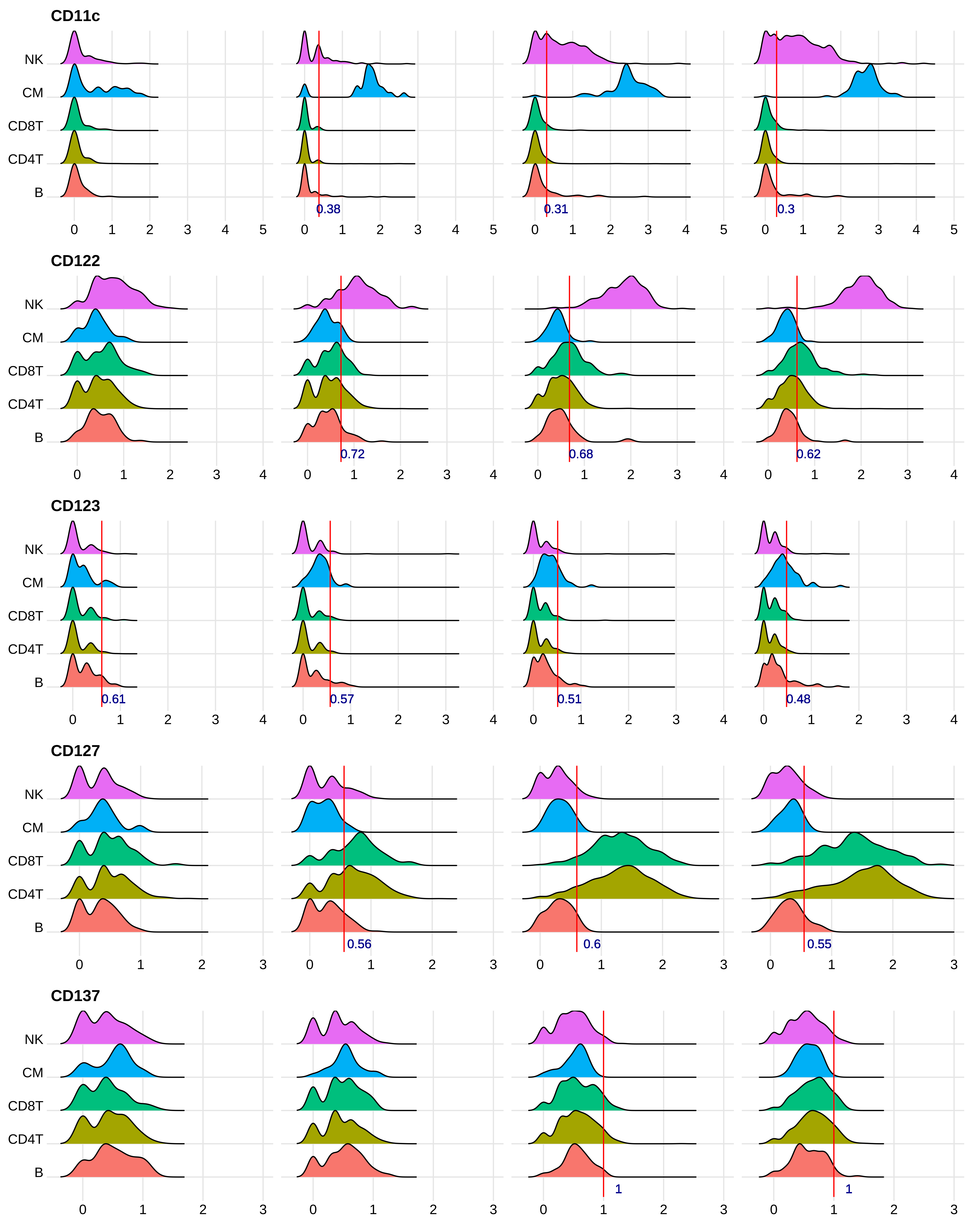
CD11c
NK
CM
CD8T
CD4T
B
0.38
0.31
0.3
0 1 2 3 4 5
CD122
NK
CM
CD8T
CD4T
B
0.72
0.68
0.62
0 1 2 3 4
CD123
NK
CM
CD8T
CD4T
B
0.61
0.57
0.51
0.48
0 1 2 3 4
CD127
NK
CM
CD8T
CD4T
B
0.56
0.6
0.55
0 1 2 3
CD137
NK
CM
CD8T
CD4T
B
1
1
0 1 2 3

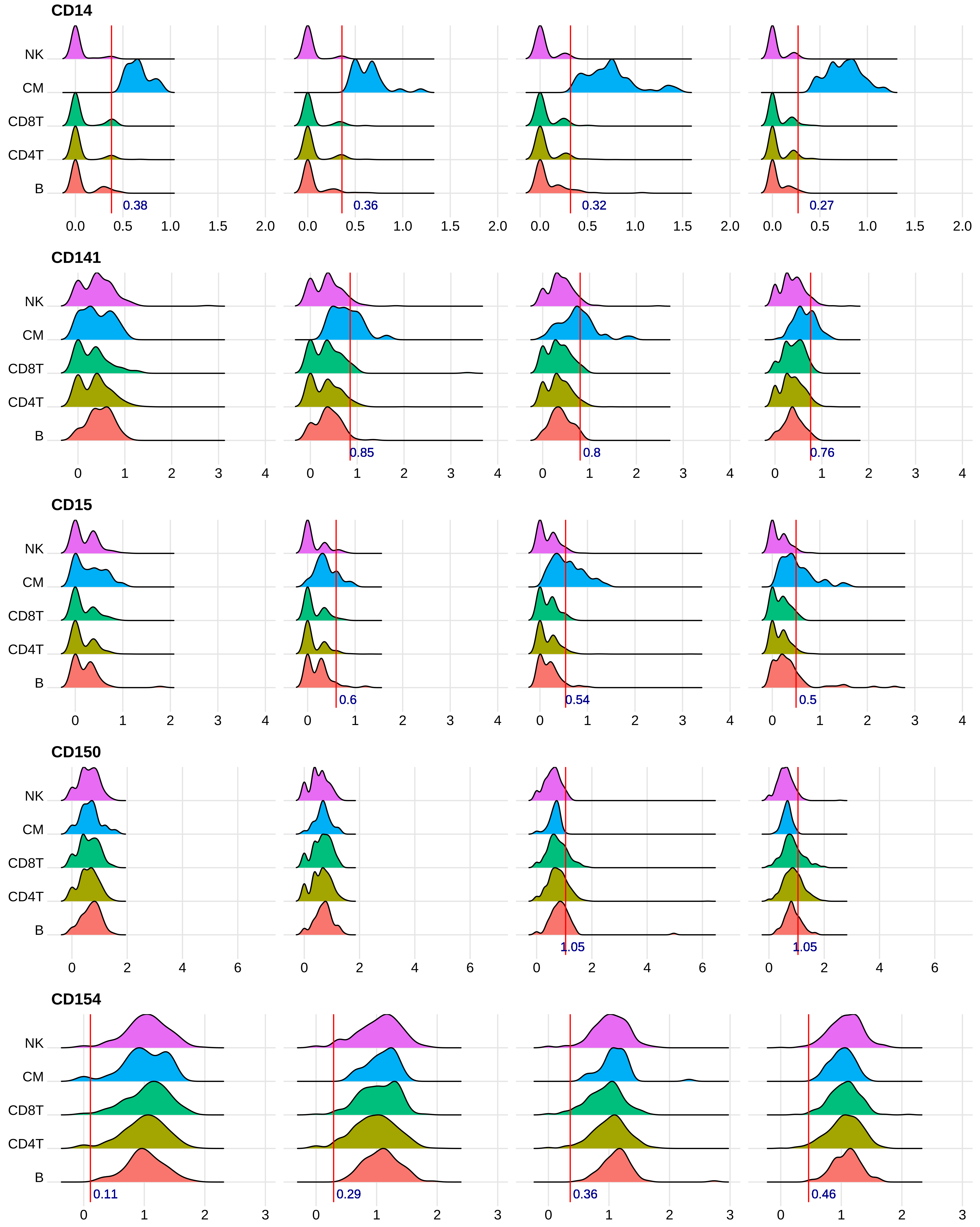
CD14
NK
CM
CD8T
CD4T
B
0.38
0.36
0.32
0.27
0.0 0.5 1.0 1.5 2.0
CD141
0.85
0.8
0.76
0 1 2 3 4
CD15
0.6
0.54
0.5
0 1 2 3 4
CD150
1.05
1.05
0 2 4 6
CD154
0.11
0.29
0.36
0.46
0 1 2 3

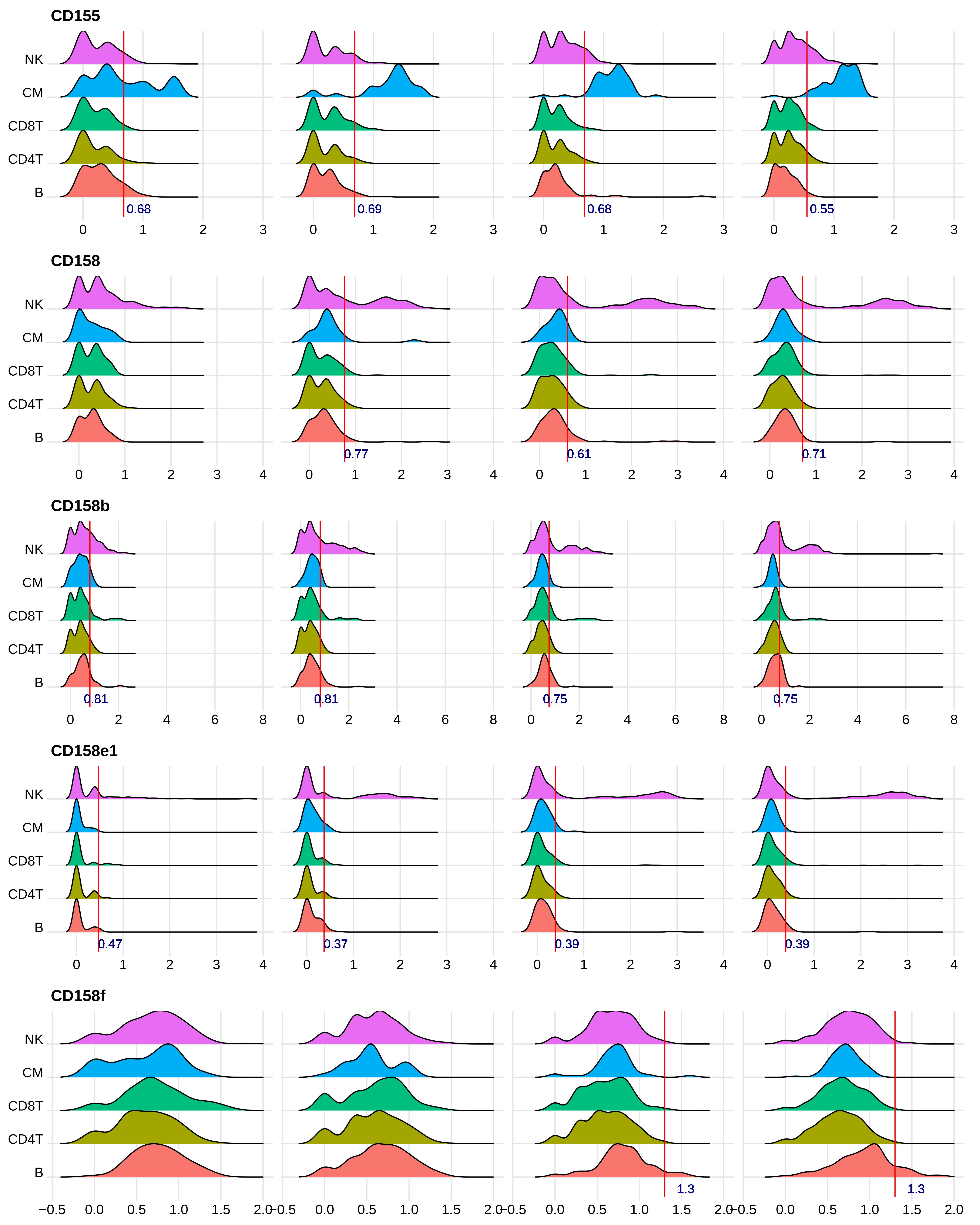
CD155
NK
CM
CD8T
CD4T
B
0.68
0.69
0.68
0.55
CD158
0.77
0.61
0.71
CD158b
0.81
0.81
0.75
0.75
CD158e1
0.47
0.37
0.39
0.39
CD158f
1.3
1.3

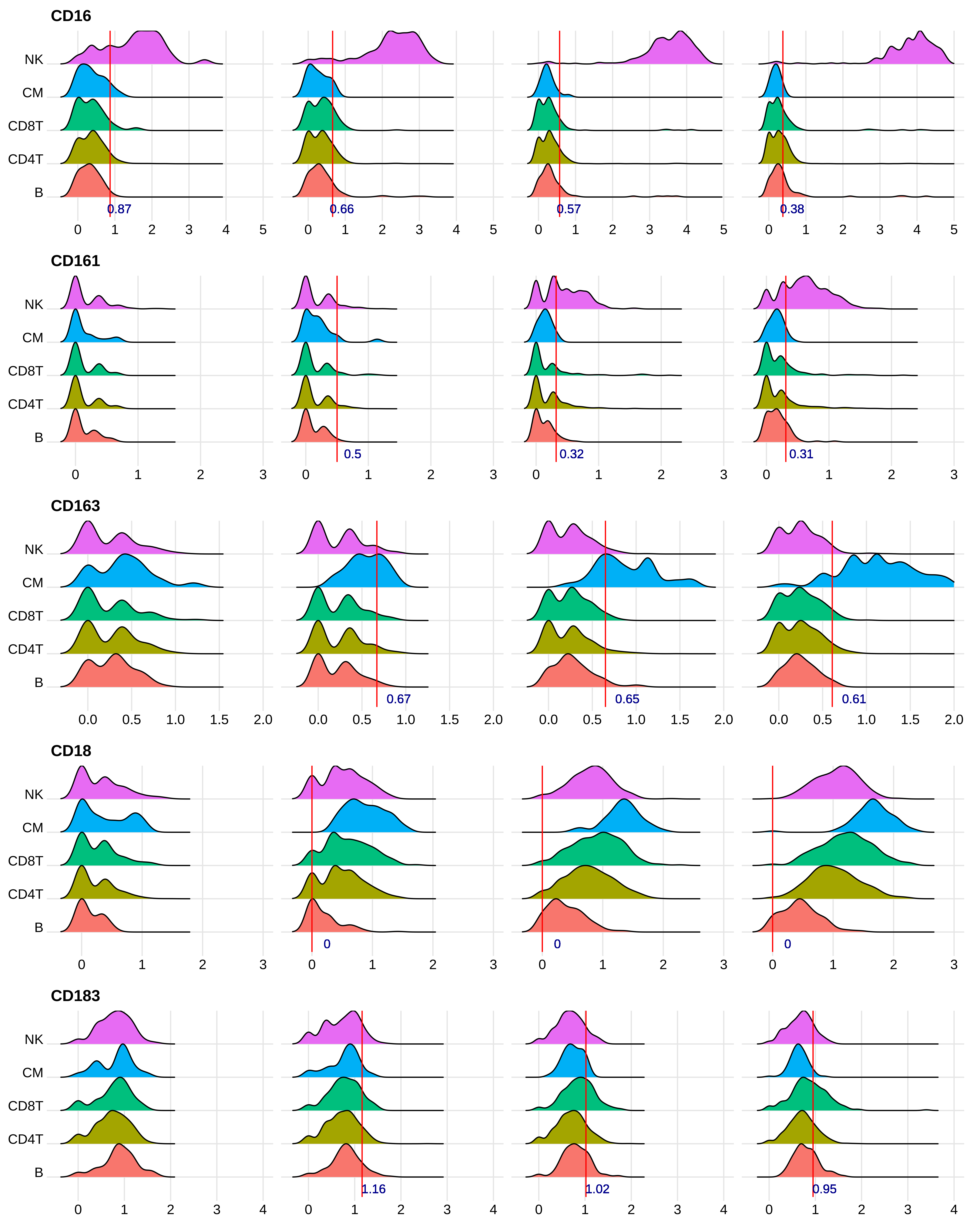

CD16
NK
CM
CD8T
CD4T
B
0.87
0.66
0.57
0.38
CD161
0.5
0.32
0.31
CD163
0.67
0.65
0.61
CD18
0
0
0
CD183
1.16
1.02
0.95

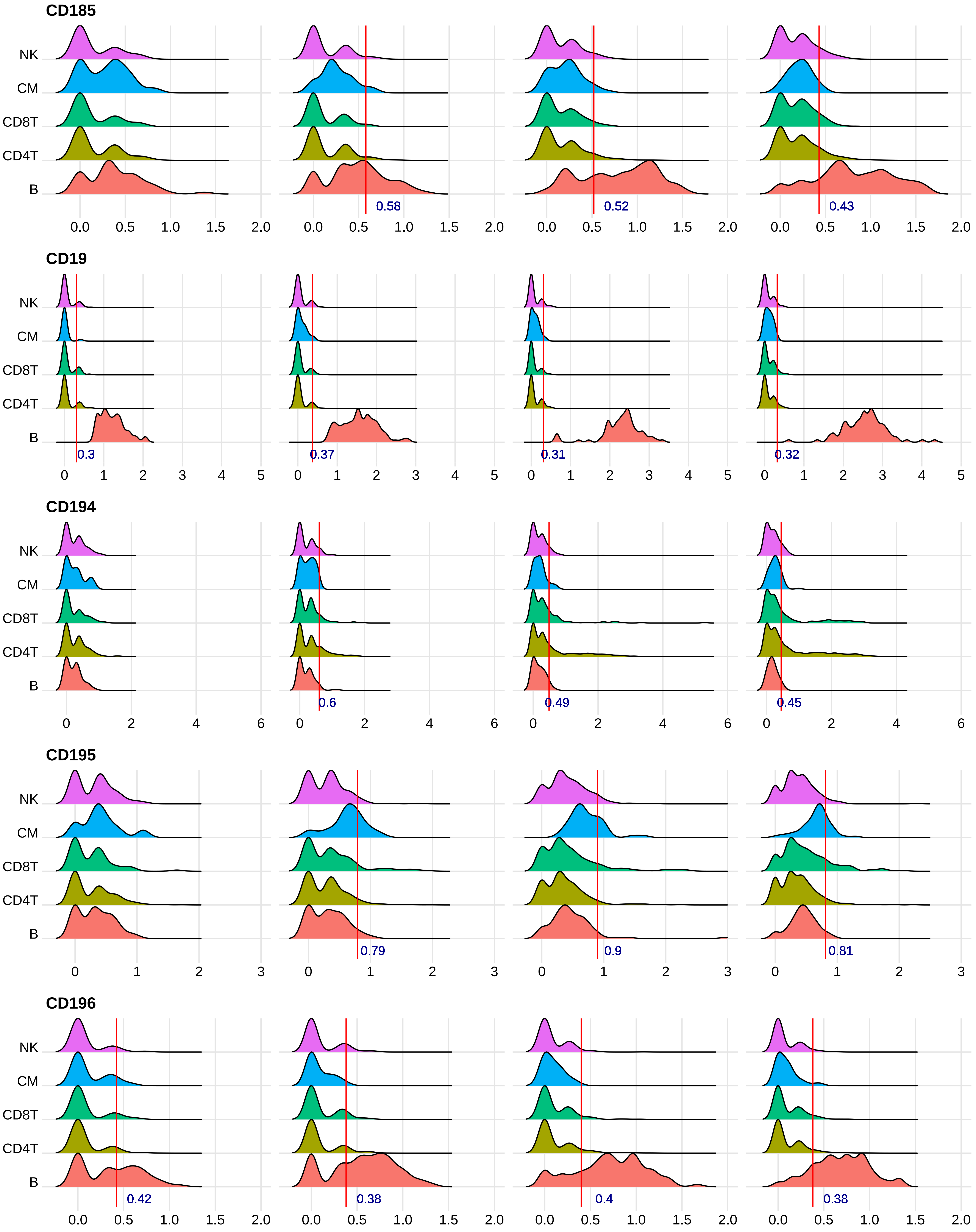

CD185
NK
CM
CD8T
CD4T
B
0.58
0.52
0.43
0.0 0.5 1.0 1.5 2.0
CD19
0.3
0.37
0.31
0.32
0 1 2 3 4 5
CD194
0.6
0.49
0.45
0 2 4 6
CD195
0.79
0.9
0.81
0 1 2 3
CD196
0.42
0.38
0.4
0.38
0.0 0.5 1.0 1.5 2.0

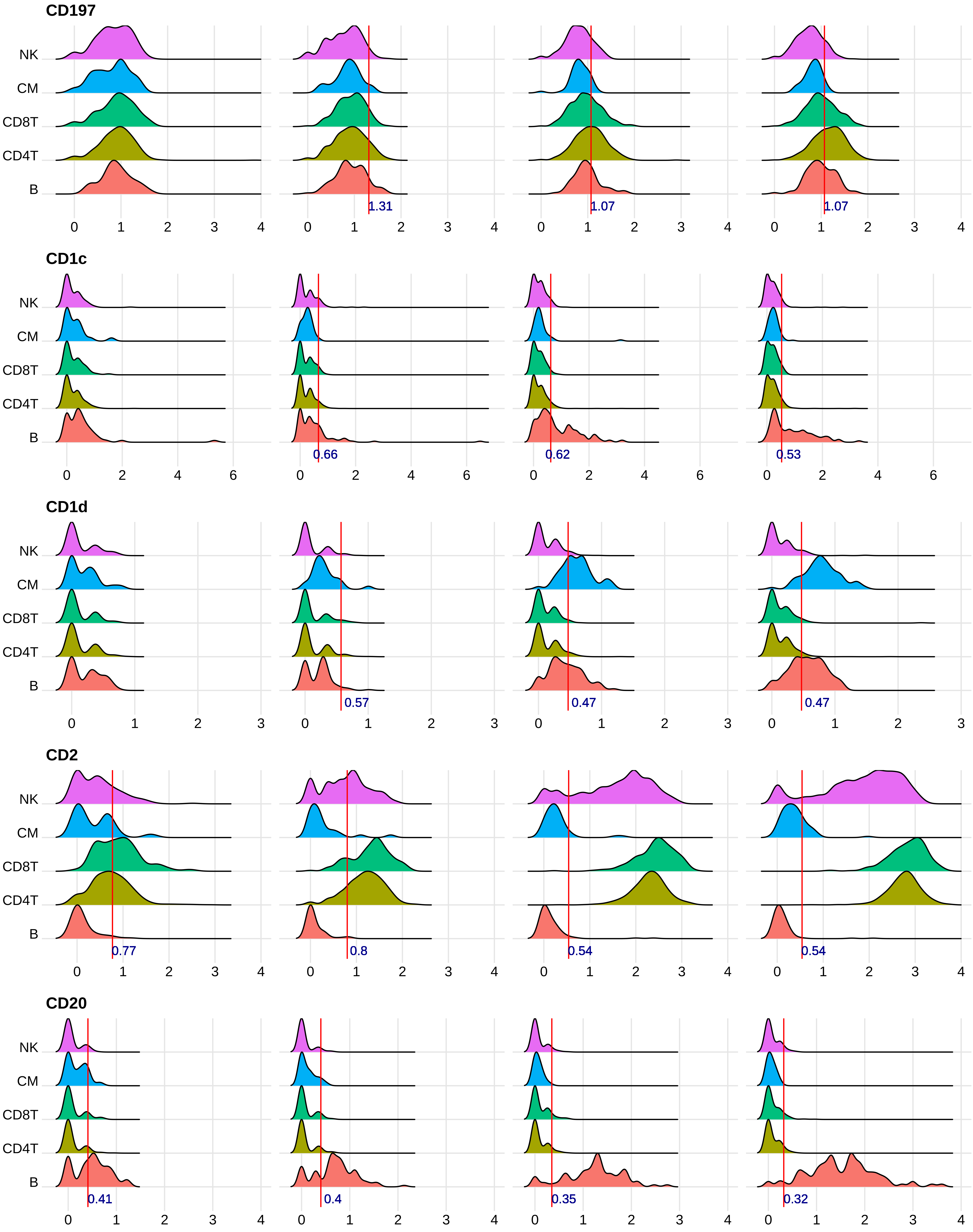
CD197
NK
CM
CD8T
CD4T
B
1.31
1.07
1.07
CD1c
0.66
0.62
0.53
CD1d
0.57
0.47
0.47
CD2
0.77
0.8
0.54
0.54
CD20
0.41
0.4
0.35
0.32

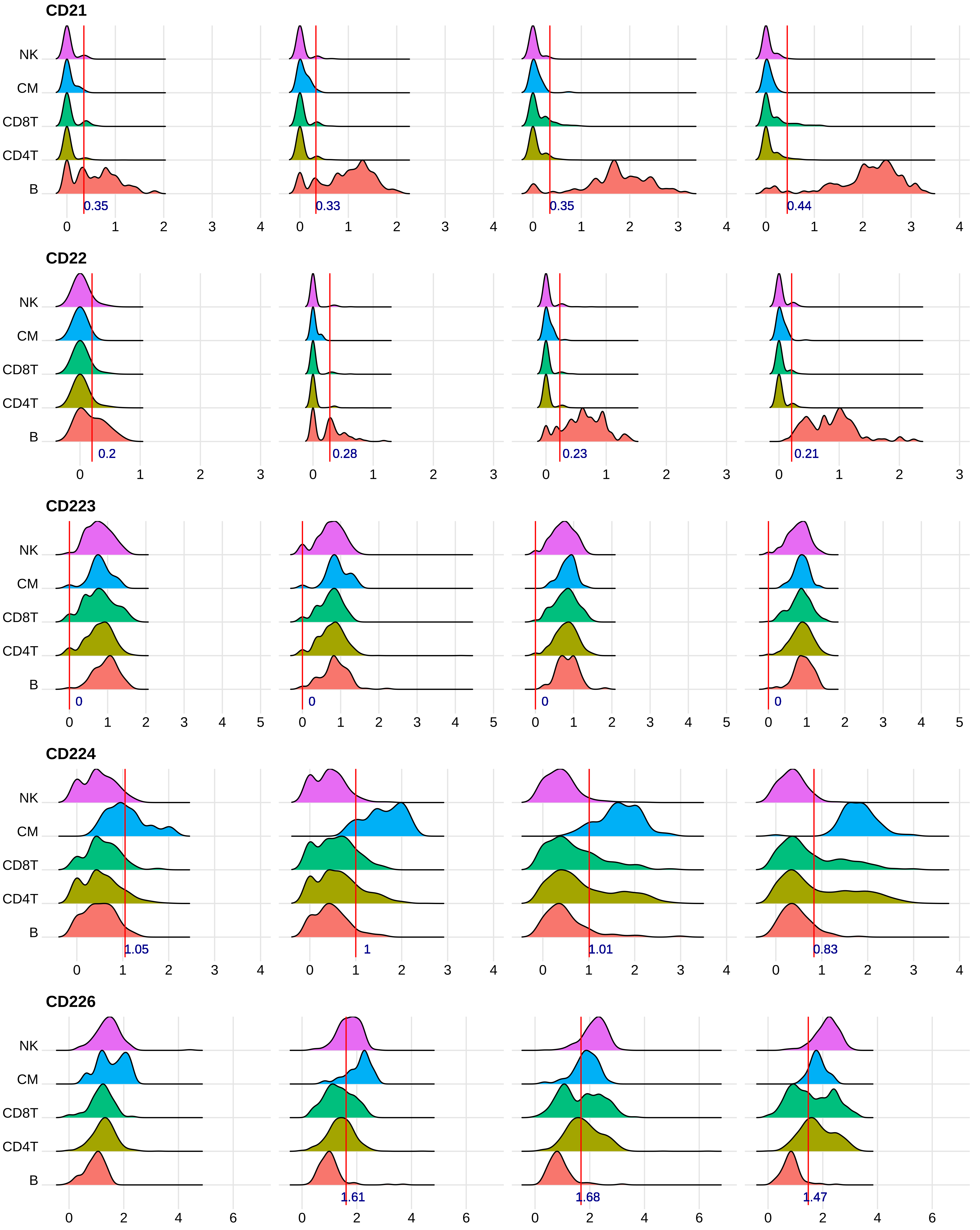
CD21
NK
CM
CD8T
CD4T
B
0.35
0.33
0.35
0.44
0 1 2 3 4
CD22
0.2
0.28
0.23
0.21
0 1 2 3
CD223
0
0
0
0
0 1 2 3 4 5
CD224
1.05
1
1.01
0.83
0 1 2 3 4
CD226
1.61
1.68
1.47
0 2 4 6

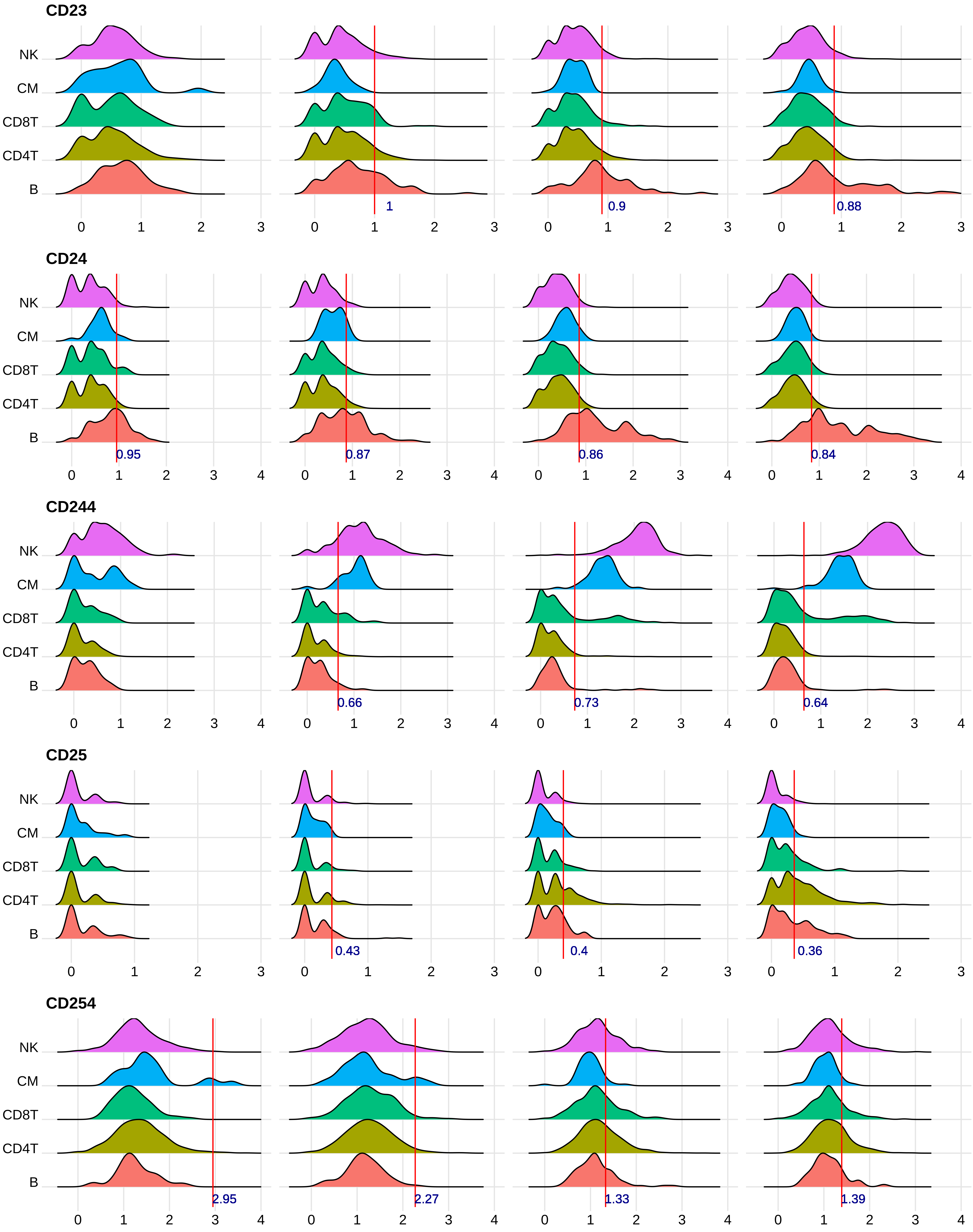

CD23
NK
CM
CD8T
CD4T
B
1
0.9
0.88
CD24
0.95
0.87
0.86
0.84
CD244
0.66
0.73
0.64
CD25
0.43
0.4
0.36
CD254
2.95
2.27
1.33
1.39

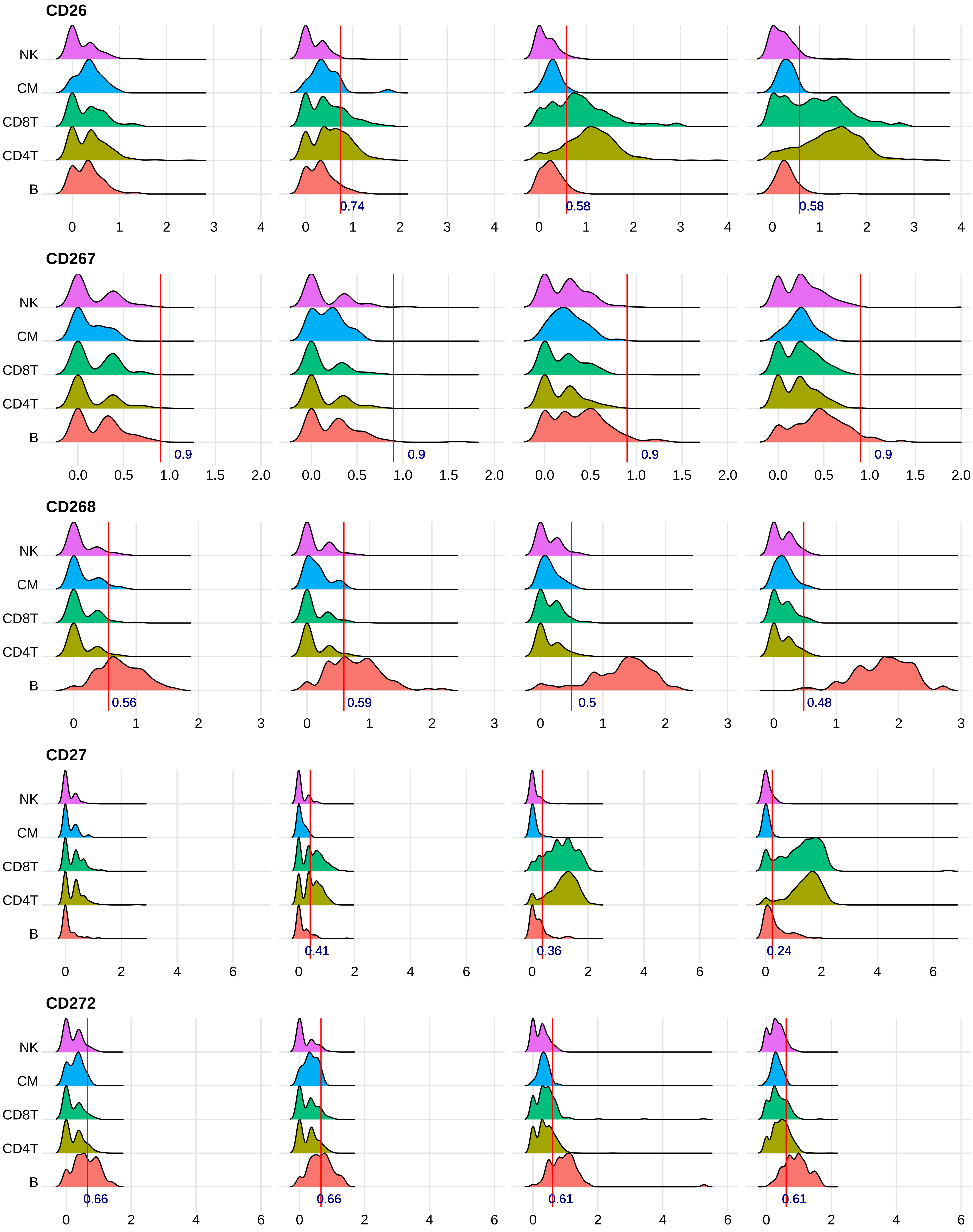
CD26
NK
CM
CD8T
CD4T
B
0.74
0.58
0.58
CD267
0.9
0.9
0.9
0.9
CD268
0.56
0.59
0.5
0.48
CD27
0.41
0.36
0.24
CD272
0.66
0.66
0.61
0.61

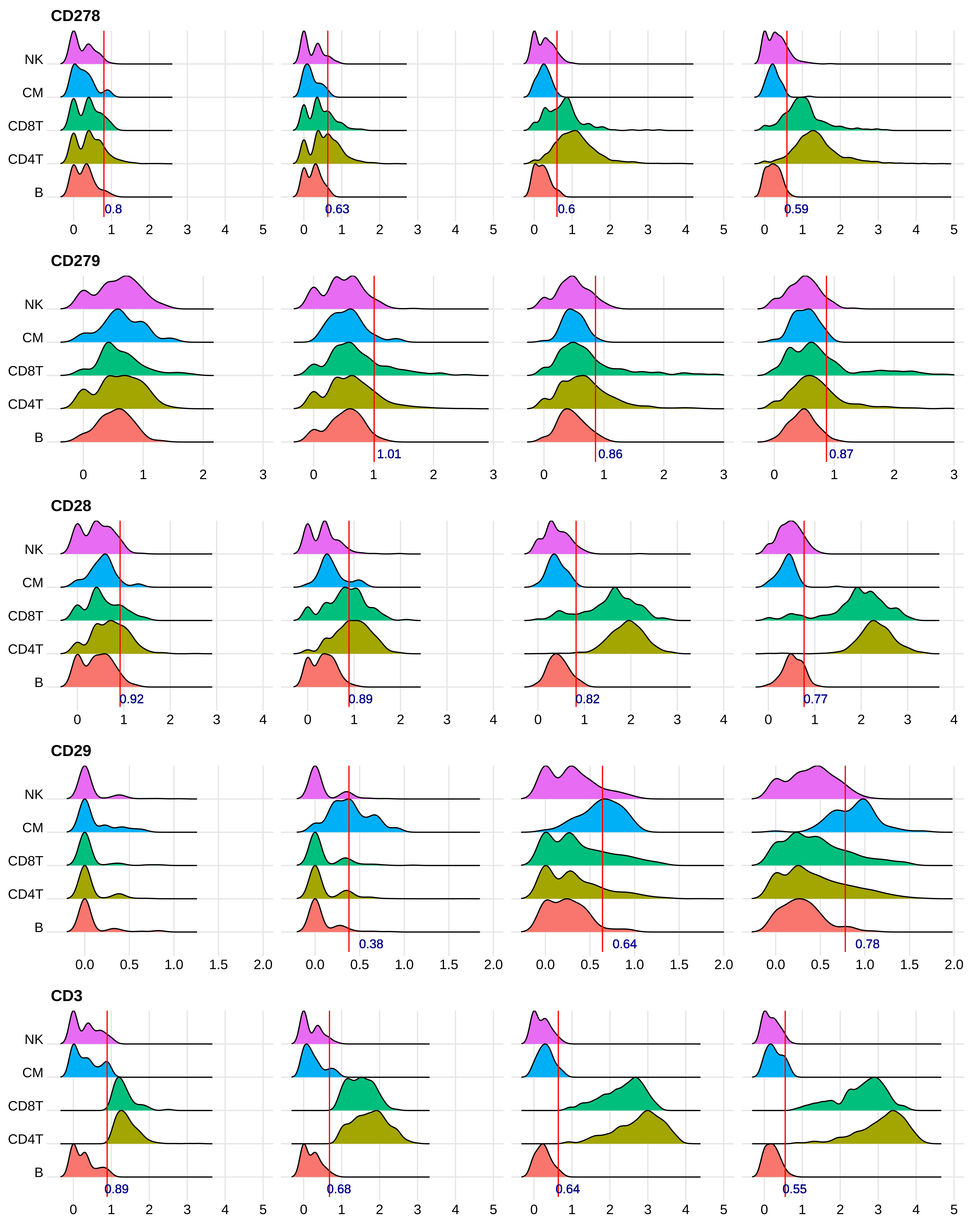

CD278
NK
CM
CD8T
CD4T
B
0.8
0.63
0.6
0.59
CD279
1.01
0.86
0.87
CD28
0.92
0.89
0.82
0.77
CD29
0.38
0.64
0.78
CD3
0.89
0.68
0.64
0.55

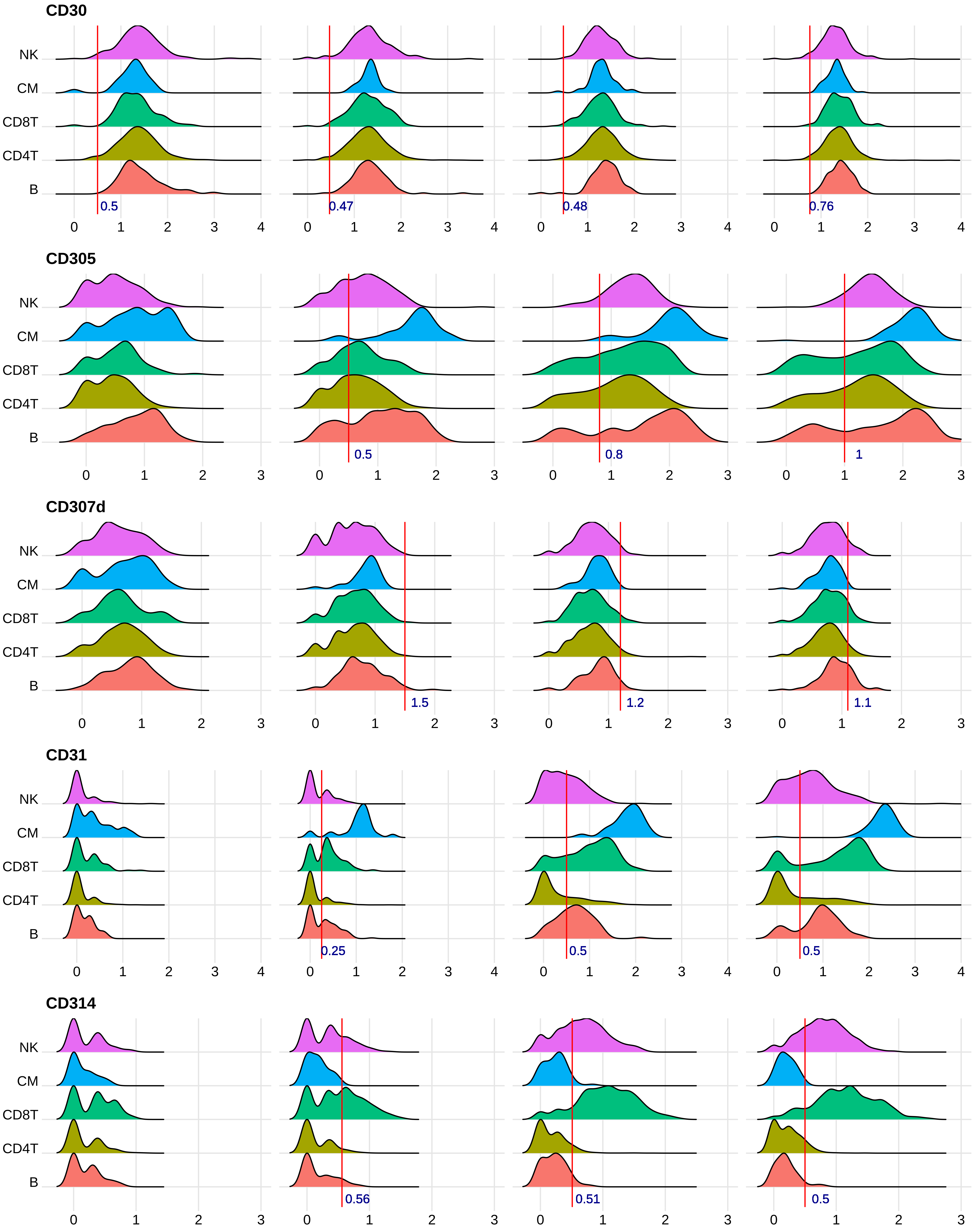
CD30
NK
CM
CD8T
CD4T
B
0.5
0.47
0.48
0.76
0 1 2 3 4
CD305
0.5
0.8
1
0 1 2 3
CD307d
1.5
1.2
1.1
CD31
0.25
0.5
0.5
CD314
0.56
0.51
0.5

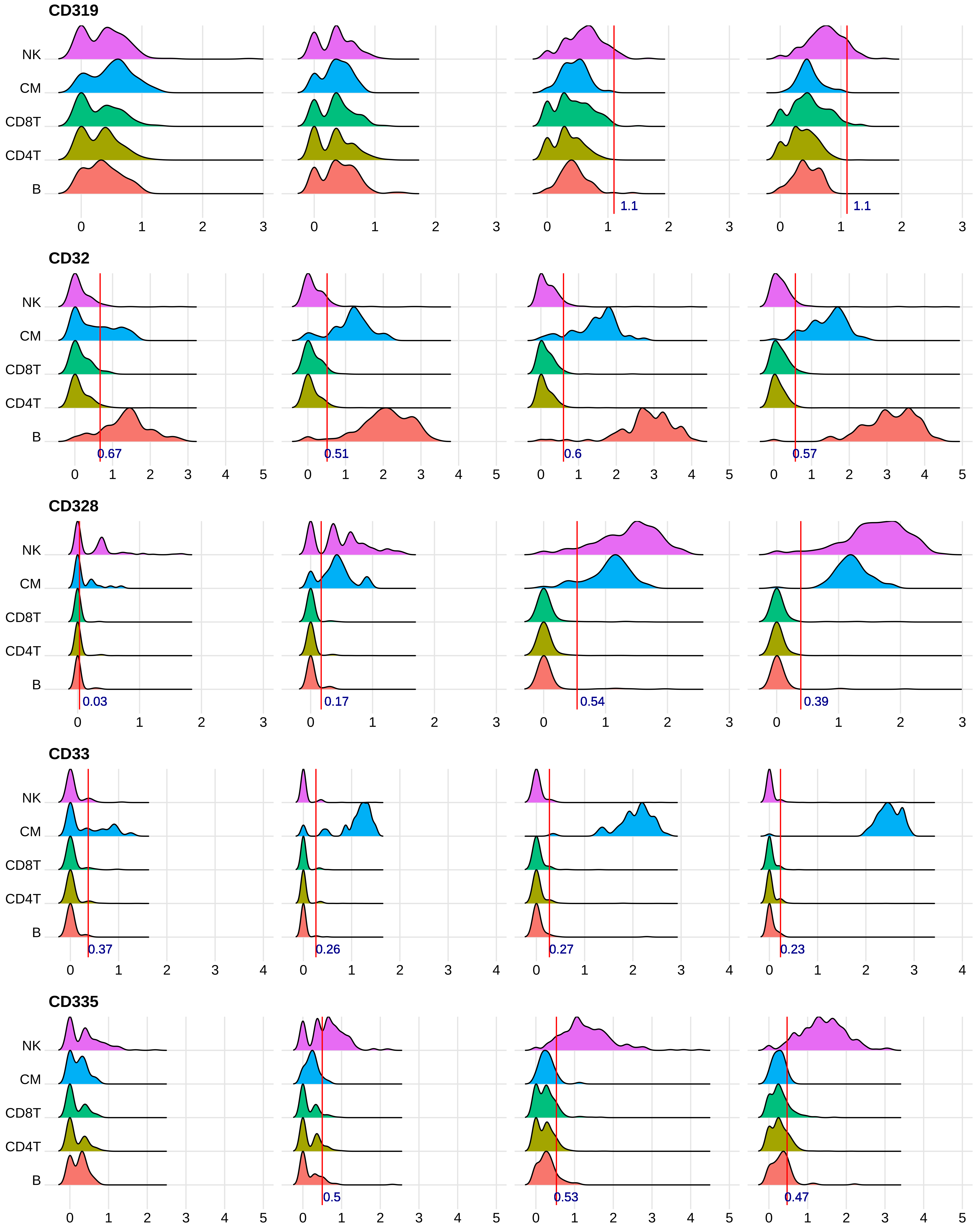
CD319
NK
CM
CD8T
CD4T
B
1.1
1.1
CD32
0.67
0.51
0.6
0.57
CD328
0.03
0.17
0.54
0.39
CD33
0.37
0.26
0.27
0.23
CD335
0.5
0.53
0.47

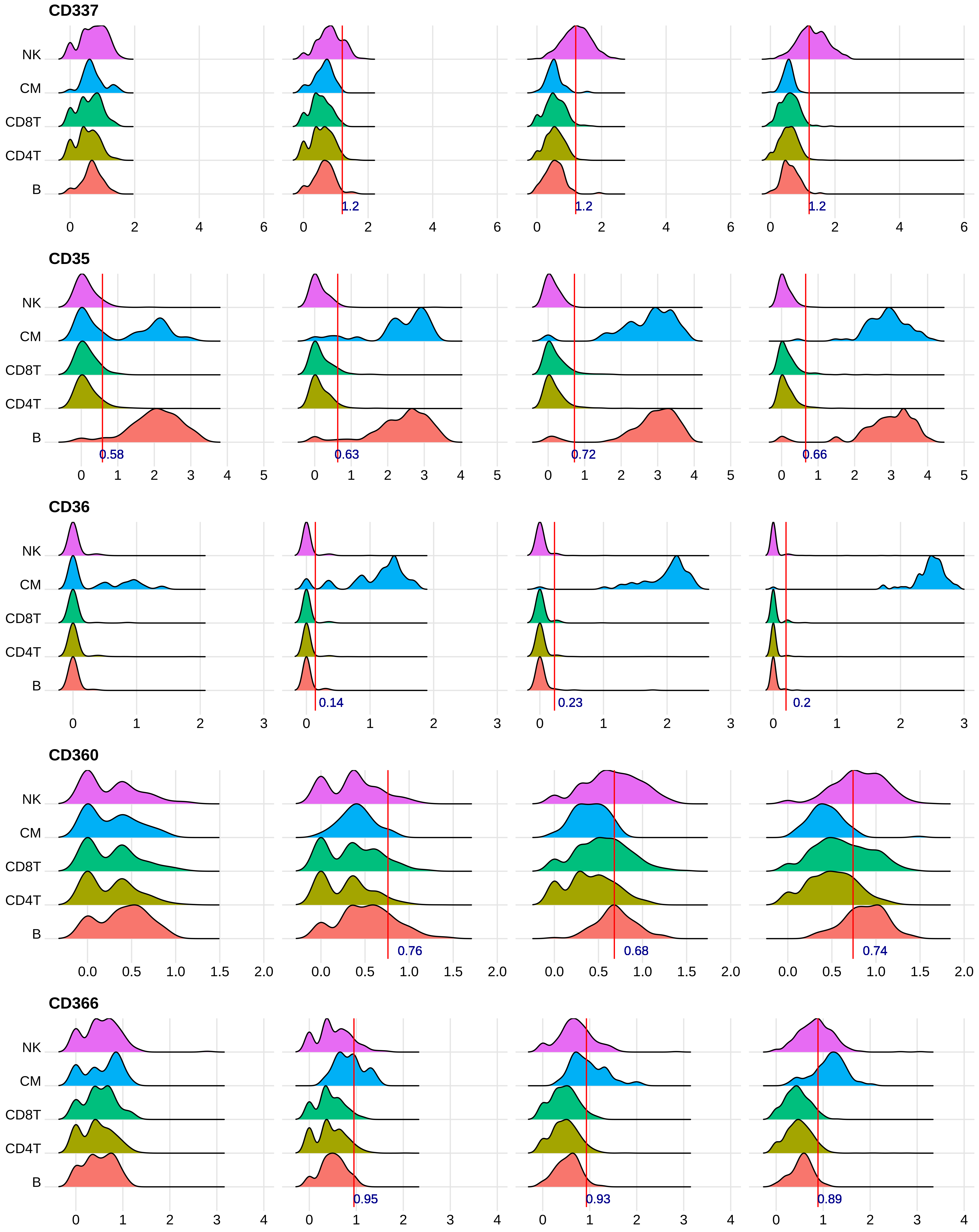

CD337
NK
CM
CD8T
CD4T
B
1.2
1.2
1.2
0 2 4 6
CD35
0.58
0.63
0.72
0.66
0 1 2 3 4 5
CD36
0.14
0.23
0.2
0 1 2 3
CD360
0.76
0.68
0.74
0.0 0.5 1.0 1.5 2.0
CD366
0.95
0.93
0.89
0 1 2 3 4

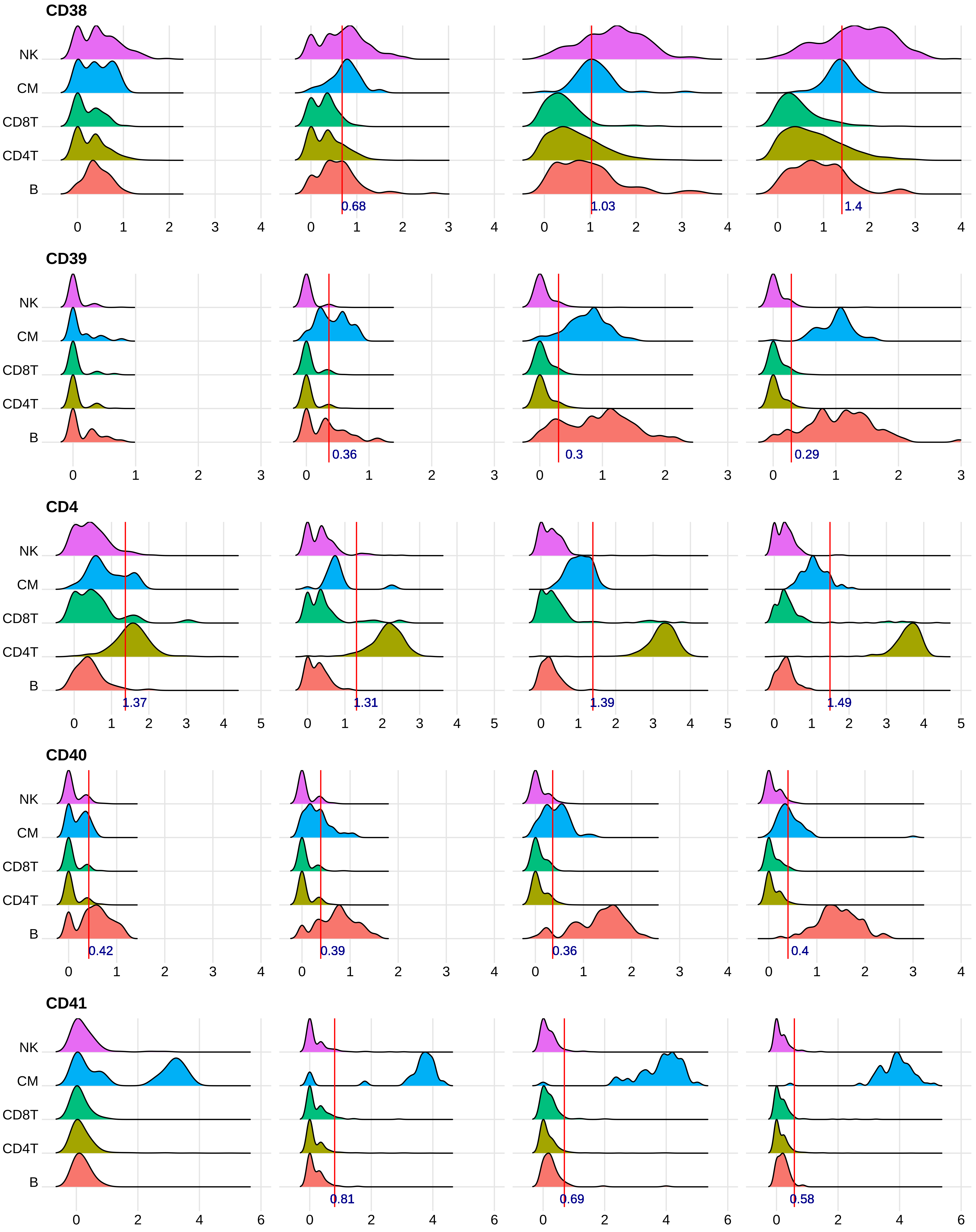

CD38
NK
CM
CD8T
CD4T
B
0.68
1.03
1.4
CD39
0.36
0.3
0.29
CD4
1.37
1.31
1.39
1.49
CD40
0.42
0.39
0.36
0.4
CD41
0.81
0.69
0.58

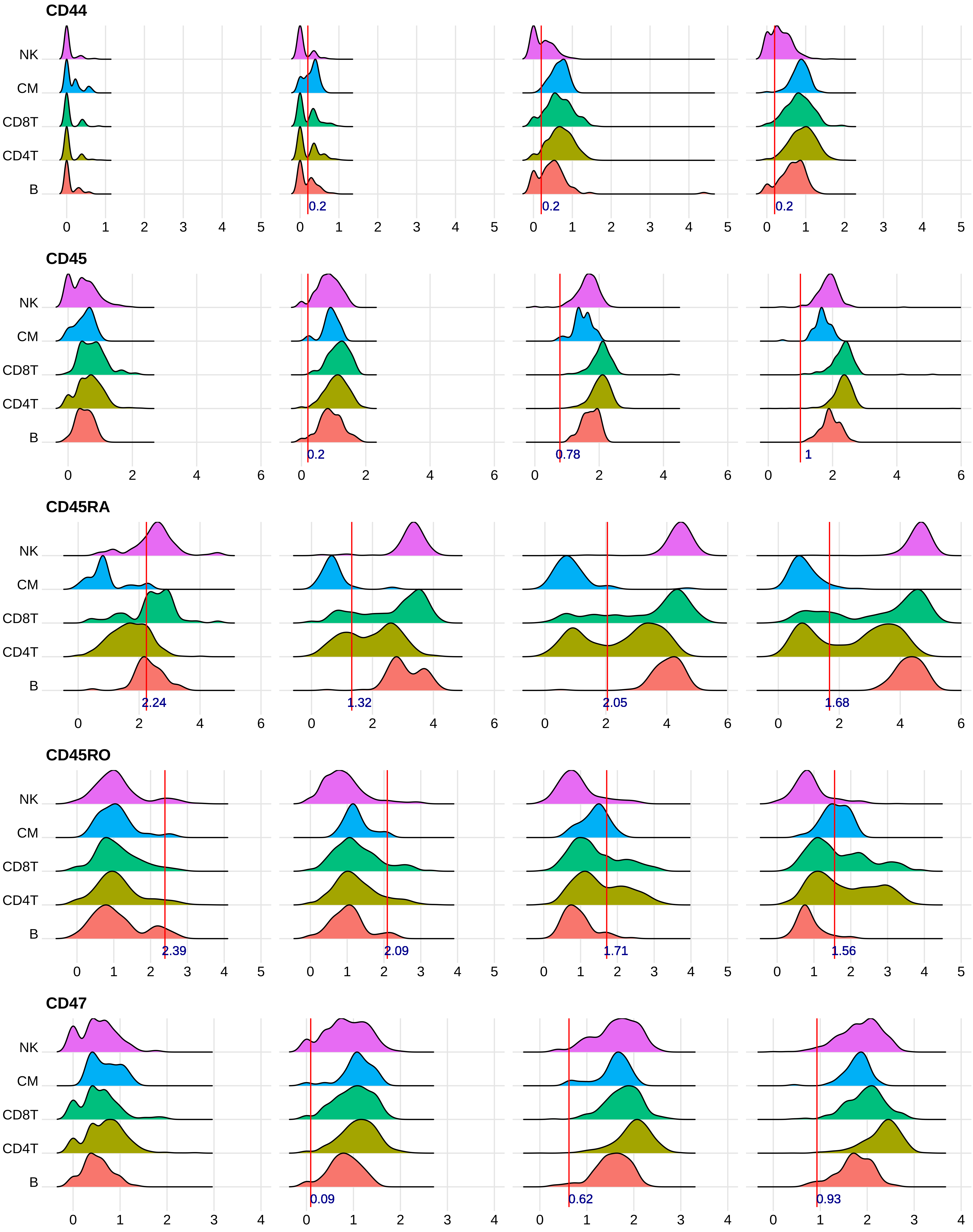
CD44
NK
CM
CD8T
CD4T
B
0.2
0.2
0.2
CD45
0.2
0.78
1
CD45RA
2.24
1.32
2.05
1.68
CD45RO
2.39
2.09
1.71
1.56
CD47
0.09
0.62
0.93

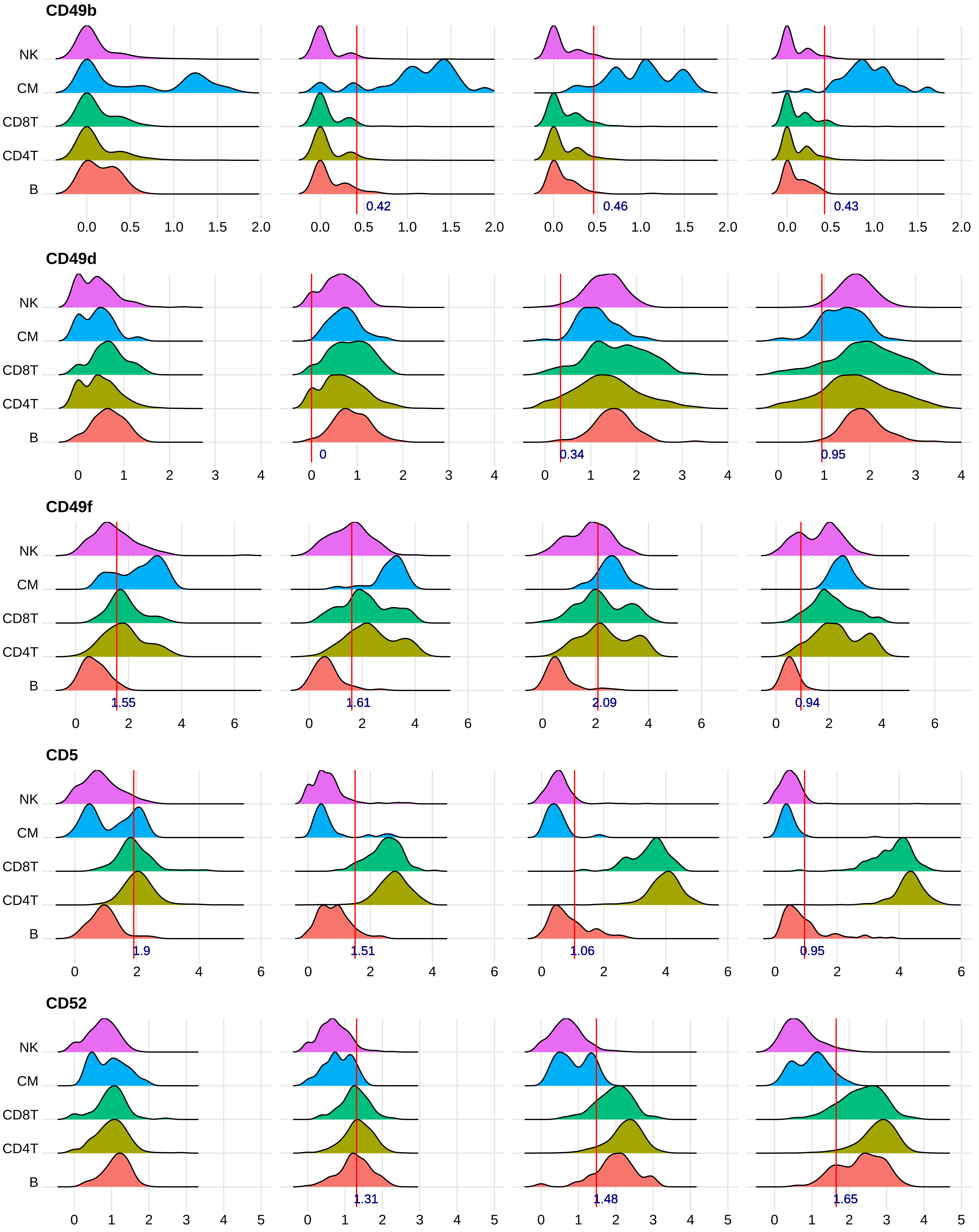

CD49b
NK
CM
CD8T
CD4T
B
0.0
0.5
1.0
1.5
2.0
0.42
0.46
0.43
CD49d
0
1
2
3
4
0
0.34
0.95
CD49f
6
1.55
1.61
2.09
0.94
CD5
1.9
1.51
1.06
0.95
CD52
5
1.31
1.48
1.65

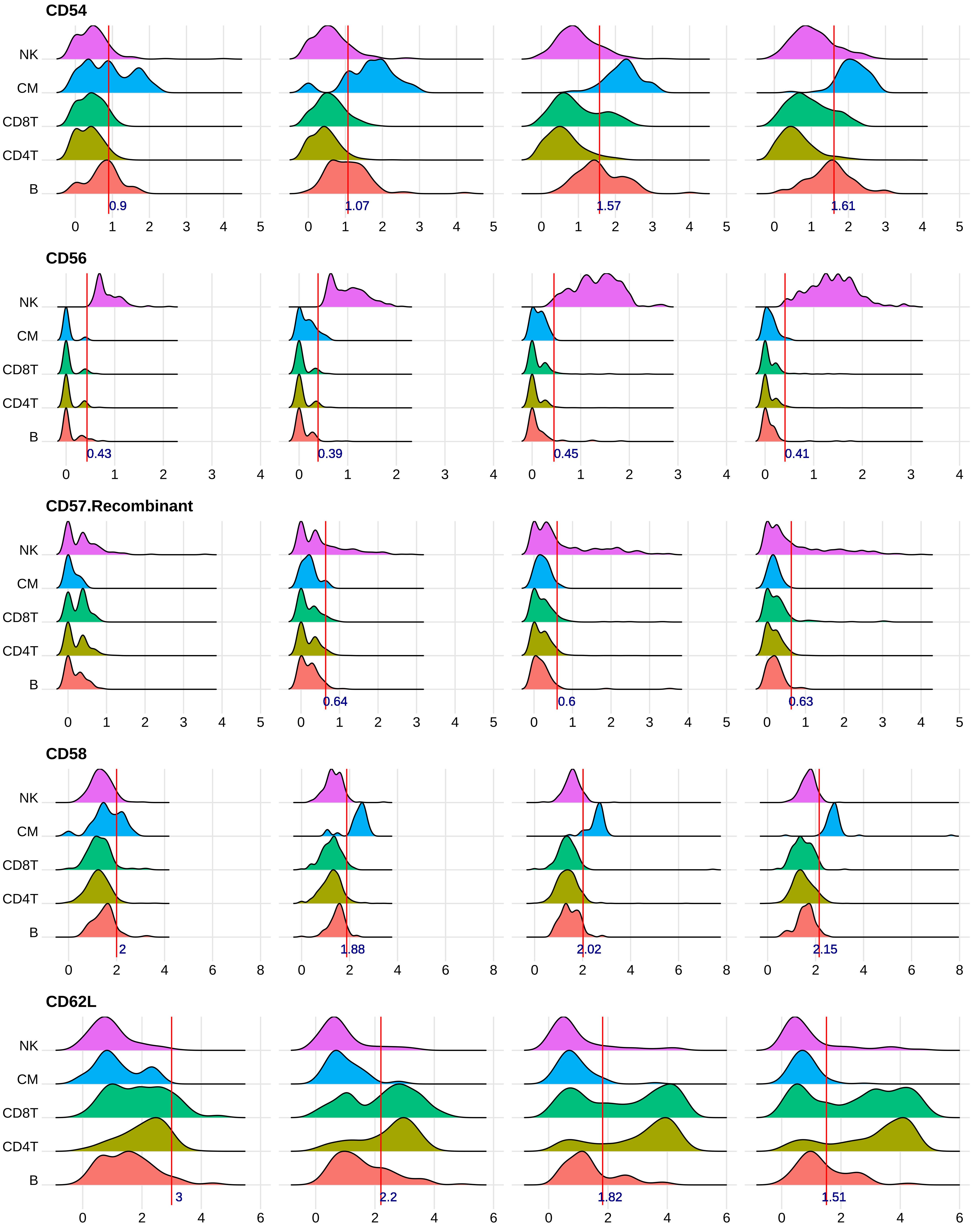
CD54
NK
CM
CD8T
CD4T
B
0.9
1.07
1.57
1.61
CD56
0.43
0.39
0.45
0.41
CD57.Recombinant
0.64
0.6
0.63
CD58
2
1.88
2.02
2.15
CD62L
3
2.2
1.82
1.51

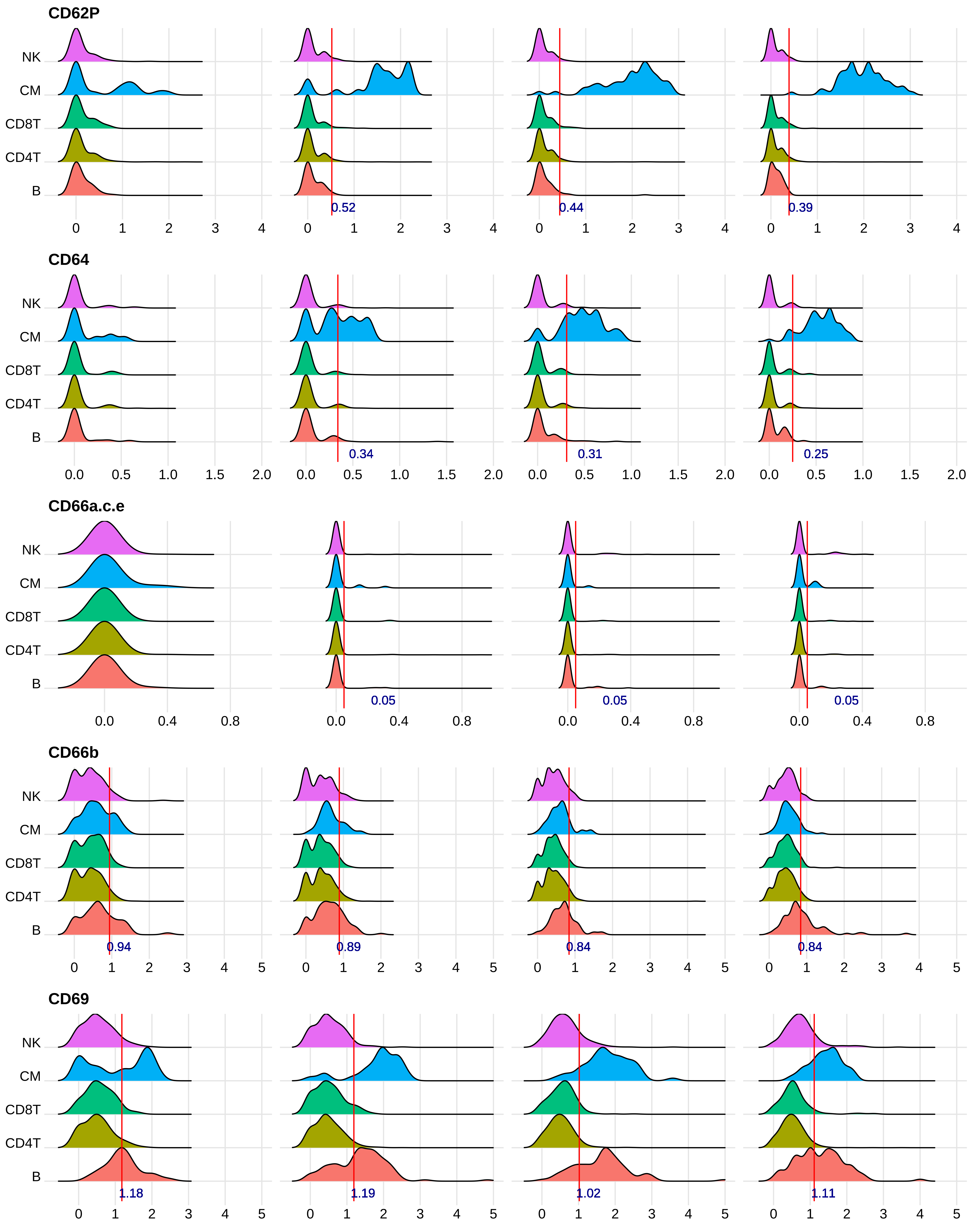
CD62P
NK
CM
CD8T
CD4T
B
0.52
0.44
0.39
0 1 2 3 4
CD64
0.34
0.31
0.25
0.0 0.5 1.0 1.5 2.0
CD66a.c.e
0.05
0.05
0.05
0.0 0.4 0.8
CD66b
0.94
0.89
0.84
0.84
0 1 2 3 4 5
CD69
1.18
1.19
1.02
1.11
0 1 2 3 4 5

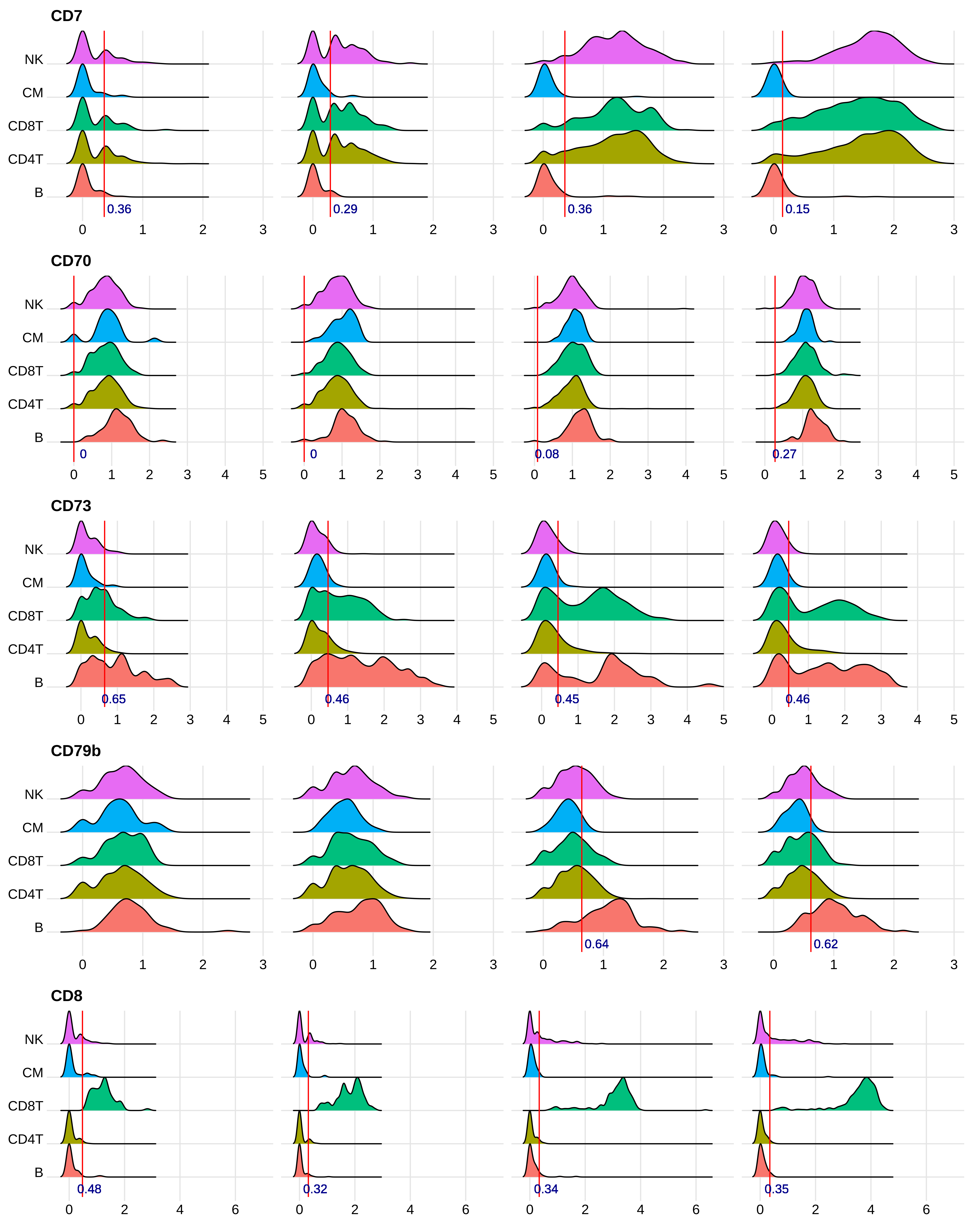
CD7
NK
CM
CD8T
CD4T
B
0.36
0.29
0.36
0.15
CD70
0
0
0.08
0.27
CD73
0.65
0.46
0.45
0.46
CD79b
0.64
0.62
CD8
0.48
0.32
0.34
0.35

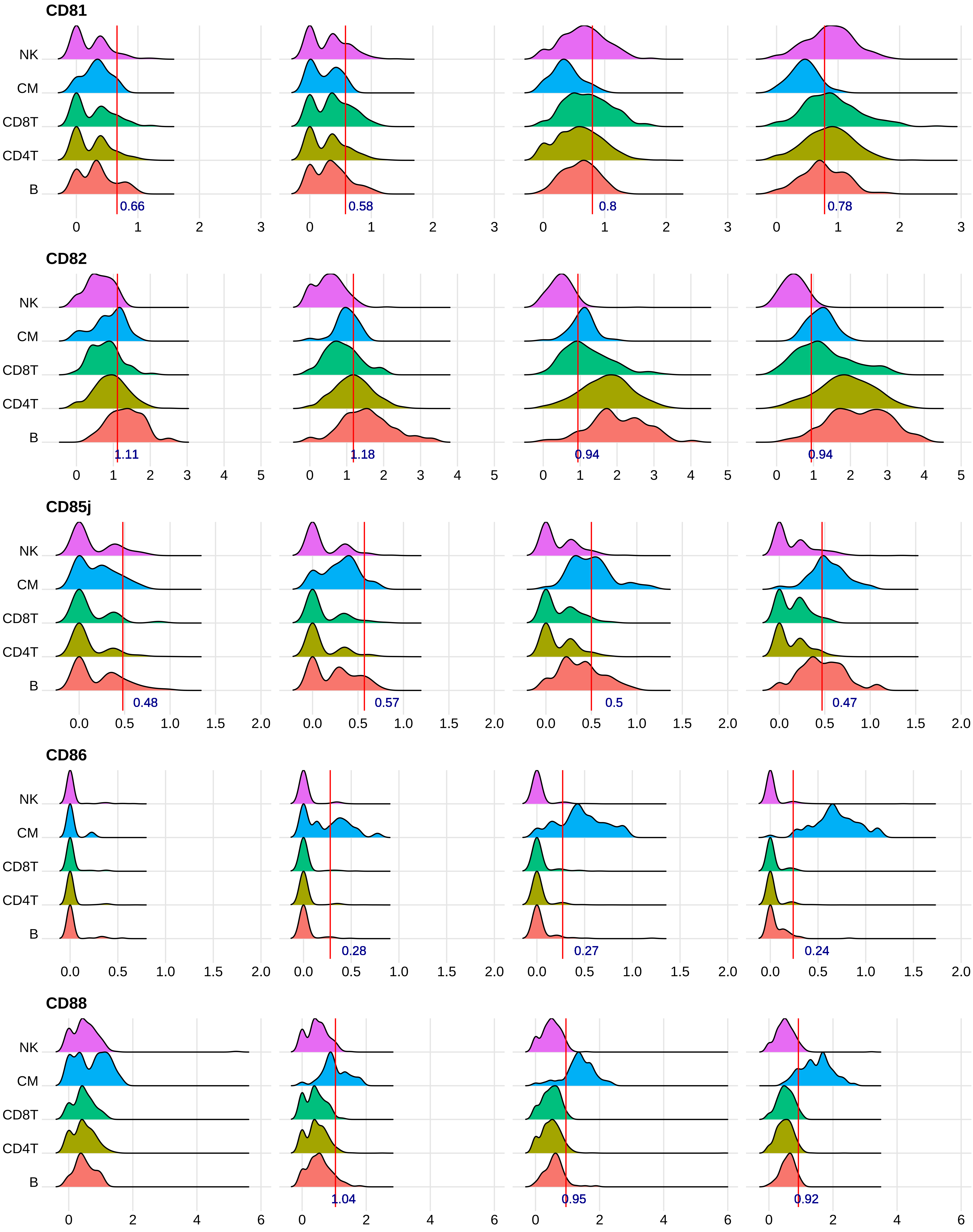

CD81
NK
CM
CD8T
CD4T
B
0.66
0.58
0.8
0.78
0
1
2
3
CD82
1.11
1.18
0.94
0.94
0
1
2
3
4
5
CD85j
0.48
0.57
0.5
0.47
0.0
0.5
1.0
1.5
2.0
CD86
0.28
0.27
0.24
CD88
1.04
0.95
0.92
0
2
4
6

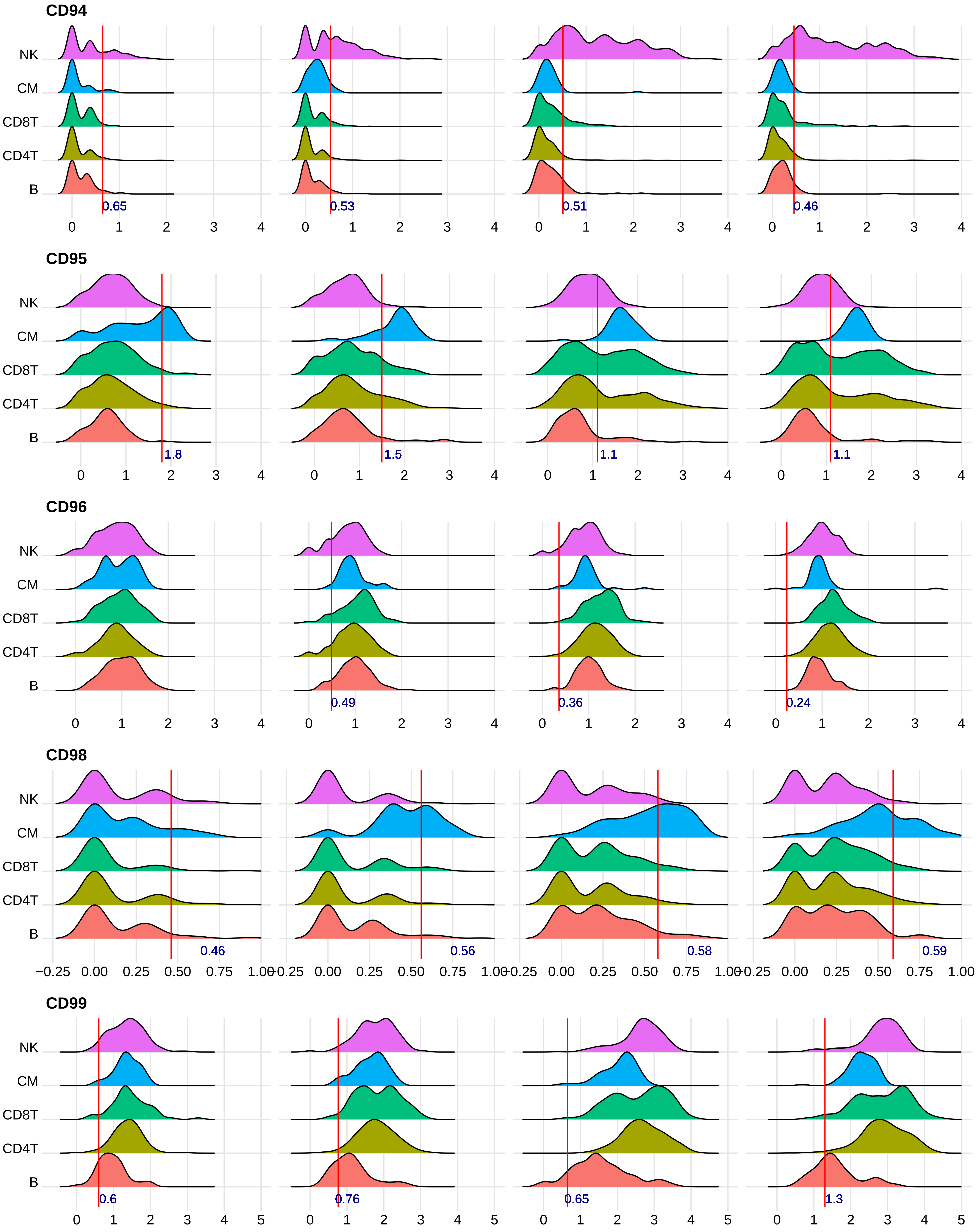
CD94
NK
CM
CD8T
CD4T
B
0.65
0.53
0.51
0.46
CD95
1.8
1.5
1.1
1.1
CD96
0.49
0.36
0.24
CD98
0.46
0.56
0.58
0.59
CD99
0.6
0.76
0.65
1.3

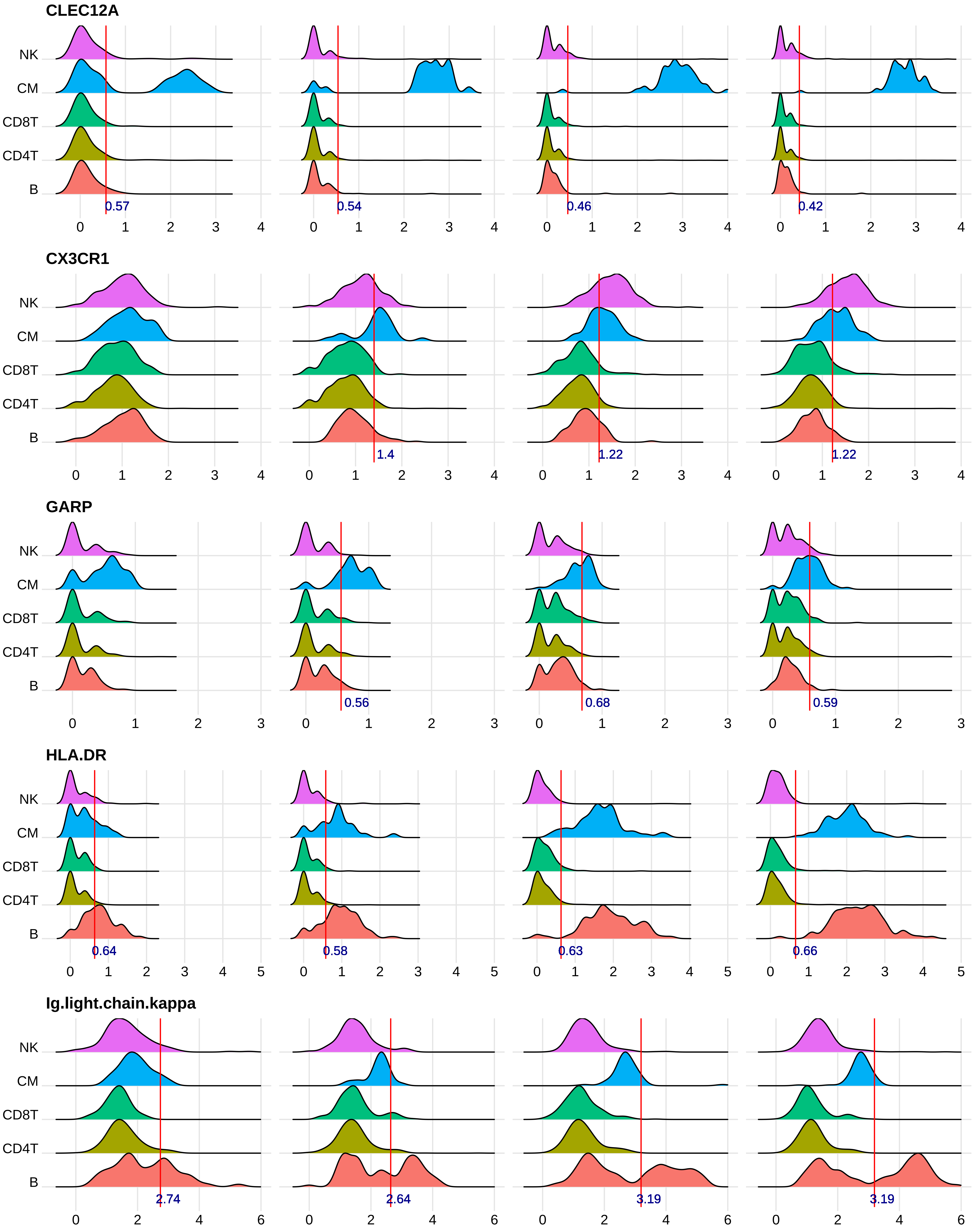
CLEC12A
NK
CM
CD8T
CD4T
B
0.57
0.54
0.46
0.42
0 1 2 3 4
CX3CR1
1.4
1.22
1.22
GARP
0.56
0.68
0.59
0 1 2 3
HLA.DR
0.64
0.58
0.63
0.66
0 1 2 3 4 5
Ig.light.chain.kappa
2.74
2.64
3.19
3.19
0 2 4 6

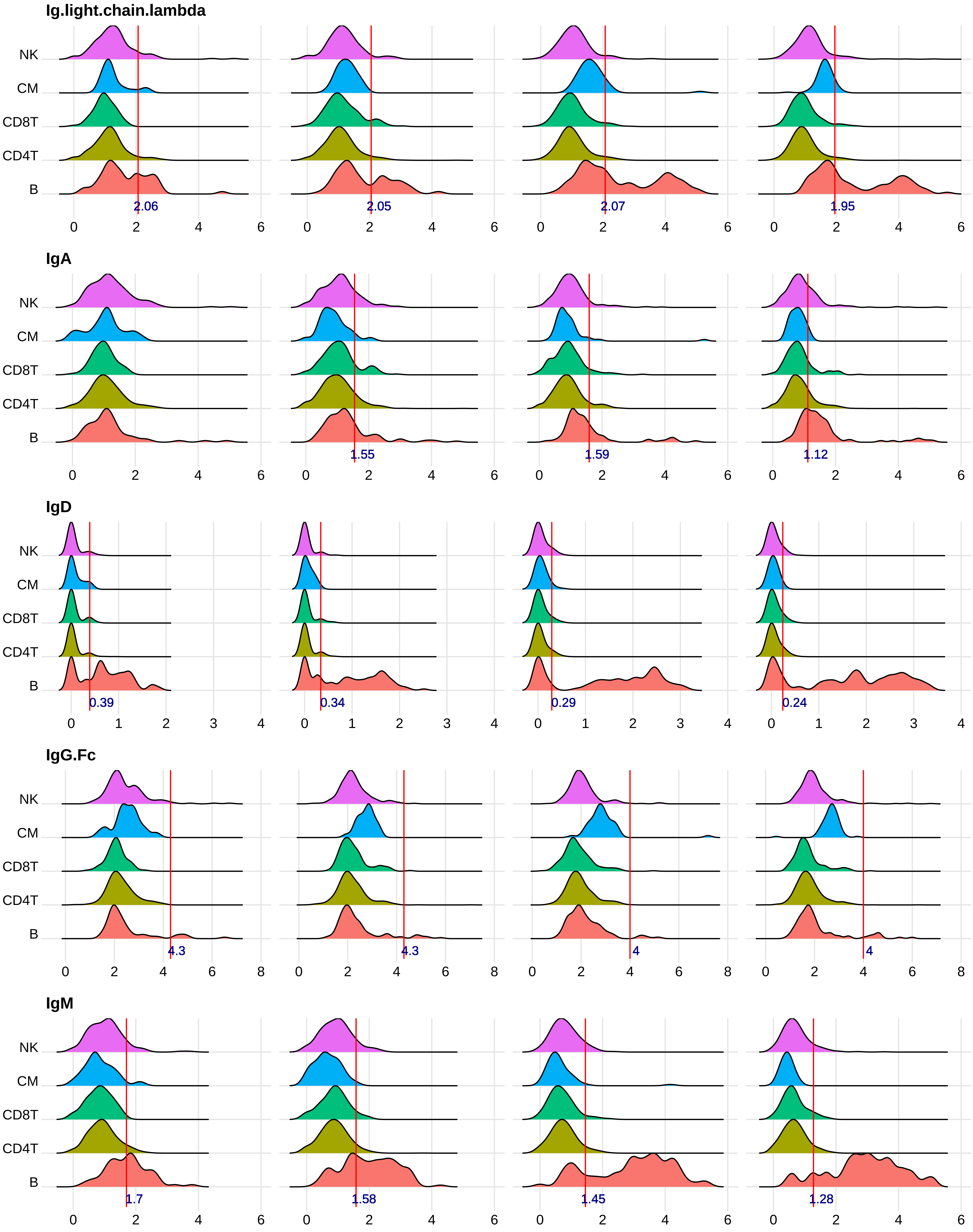
Ig.light.chain.lambda
NK
CM
CD8T
CD4T
B
2.06
2.05
2.07
1.95
IgA
1.55
1.59
1.12
IgD
0.39
0.34
0.29
0.24
IgG.Fc
4.3
4.3
4
4
IgM
1.7
1.58
1.45
1.28

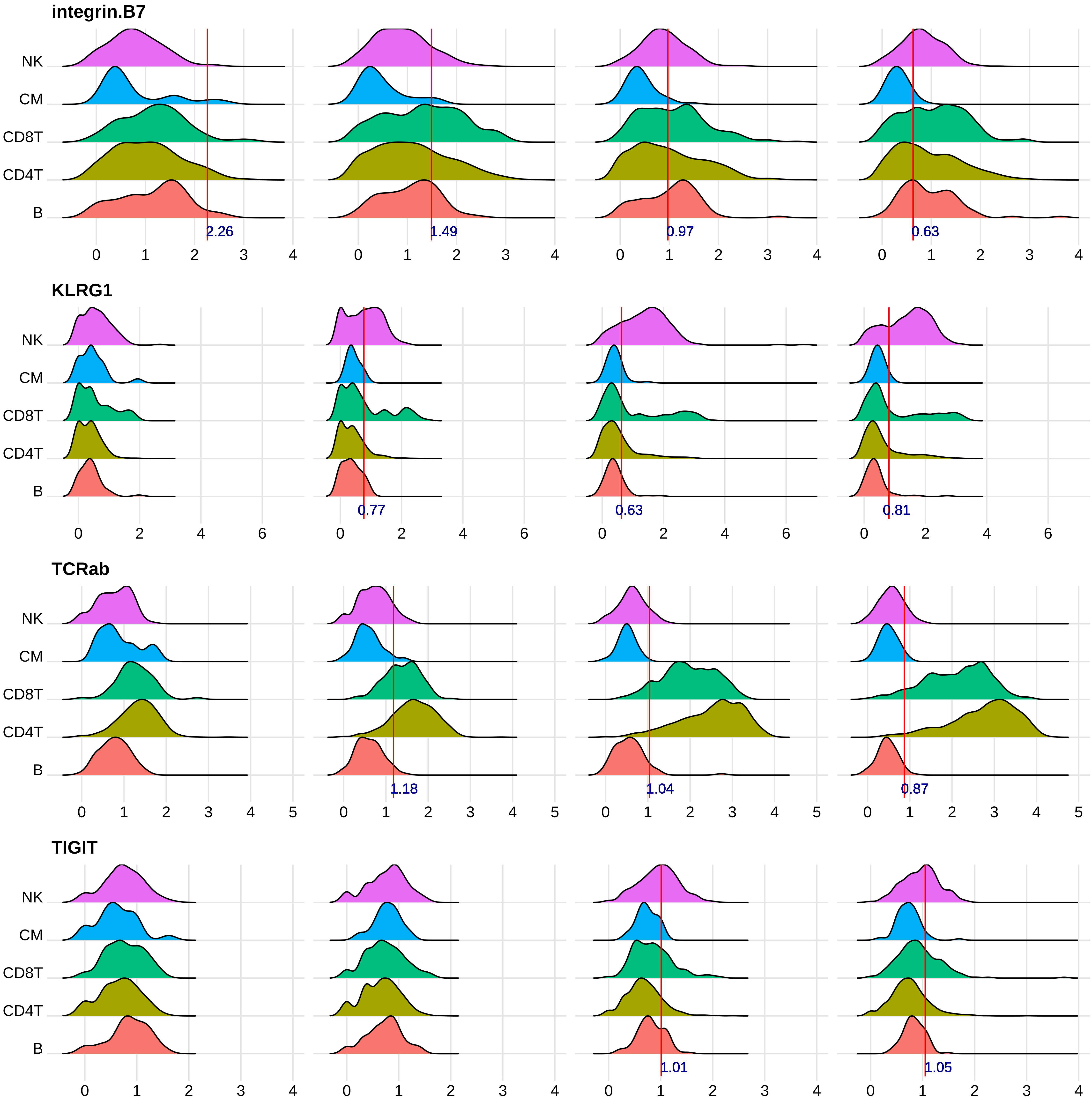
integrin.B7
KLRG1
TCRab
TIGIT
NK
CM
CD8T
CD4T
B
2.26
1.49
0.97
0.63
0.77
0.63
0.81
1.18
1.04
0.87
1.01
1.05
