# Supplementary material for: Titration of 124 antibodies using CITE-Seq on human PBMCs
Source: Sci Rep. 2022 Dec 2;12:20817. doi: 10.1038/s41598-022-24371-7 (PMC9718773; doi:10.1038/s41598-022-24371-7)
Supplement: Supplementary file 3 — Supplementary Information 3. [file 41598_2022_24371_MOESM3_ESM.pdf]

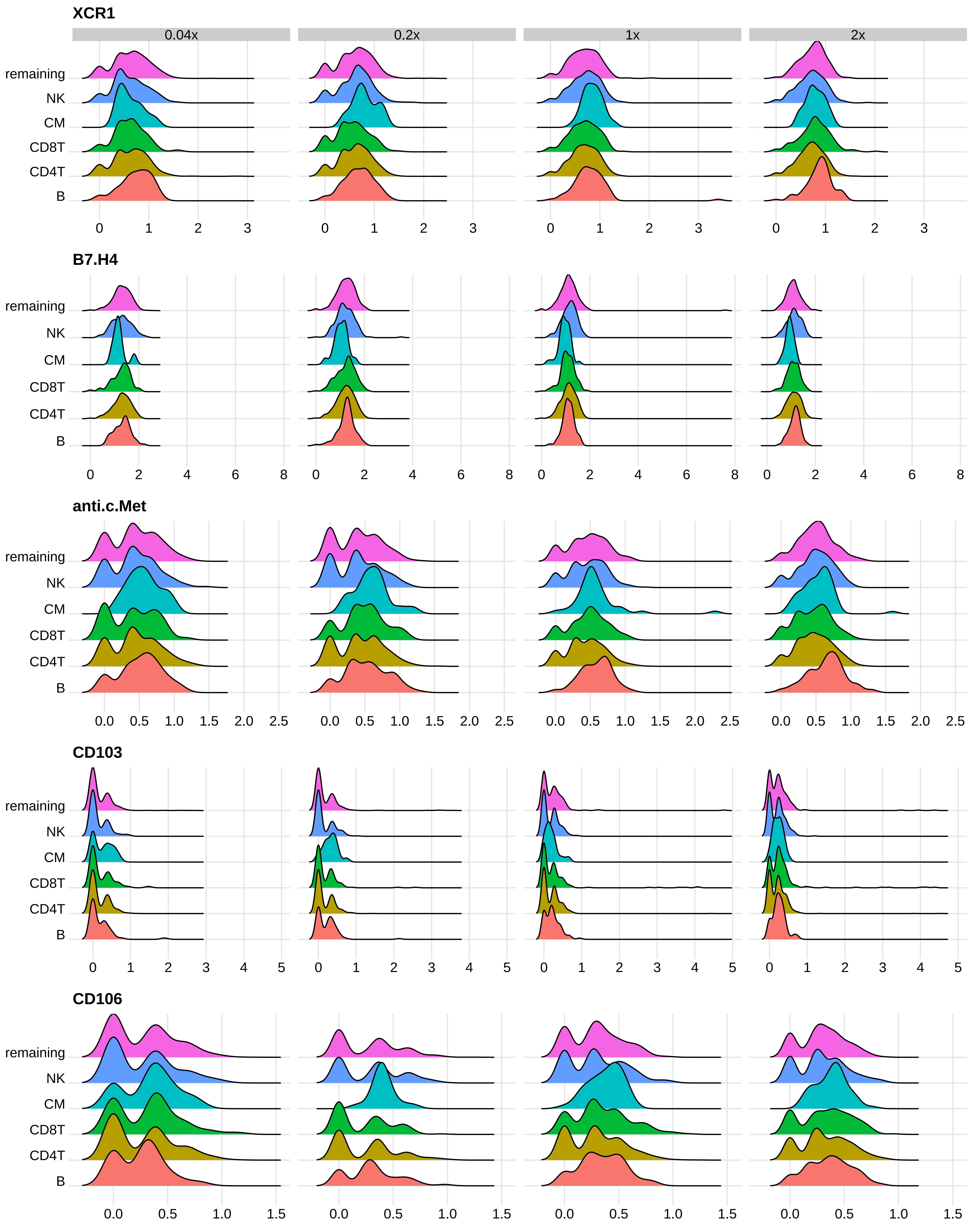
XCR1
0.04x
0.2x
1x
2x
remaining
NK
CM
CD8T
CD4T
B
0 1 2 3
B7.H4
0 2 4 6 8
anti.c.Met
0.0 0.5 1.0 1.5 2.0 2.5
CD103
0 1 2 3 4 5
CD106
0.0 0.5 1.0 1.5

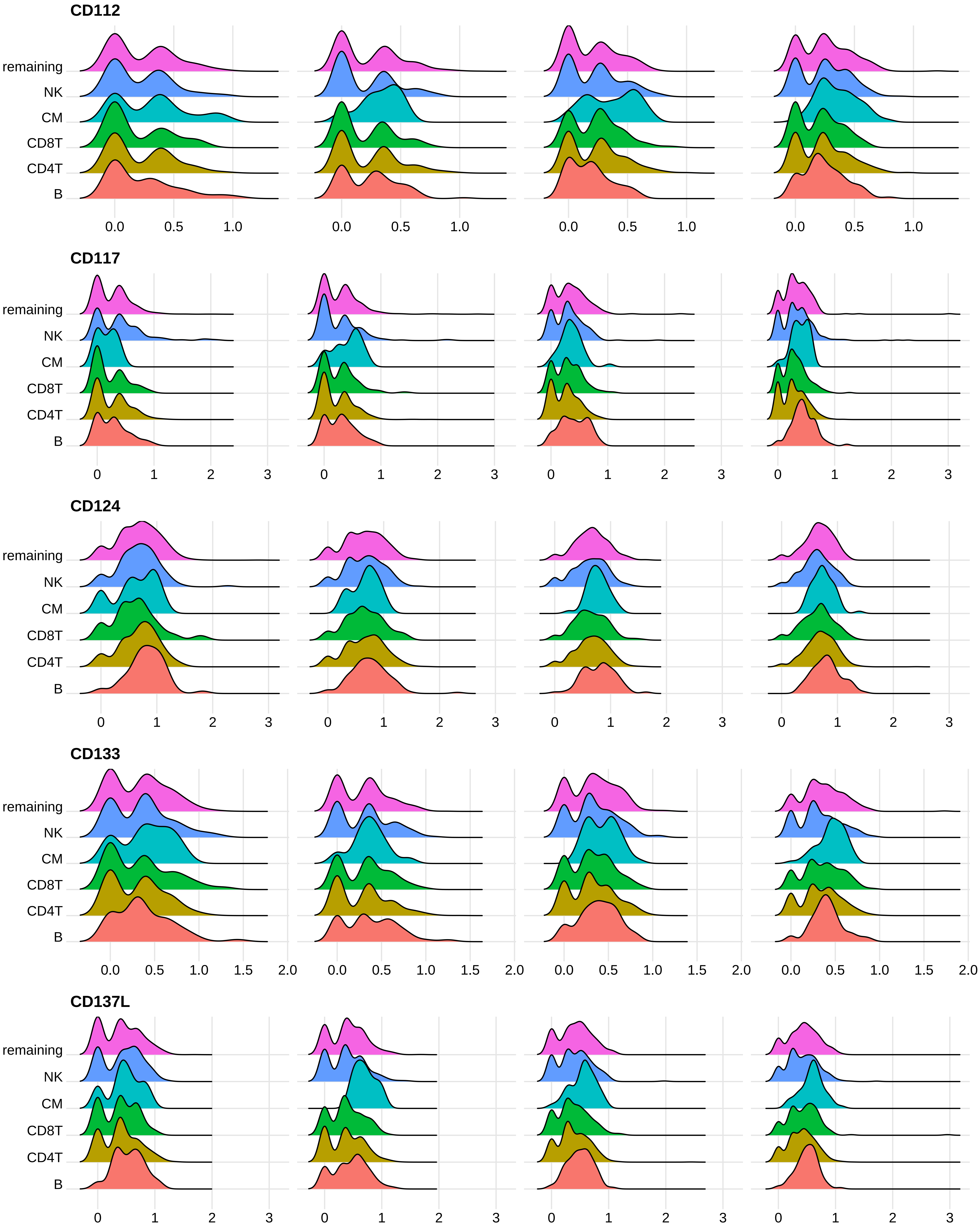

CD112
CD117
CD124
CD133
CD137L
remaining
NK
CM
CD8T
CD4T
B
0.0
0.5
1.0
1.5
2.0
0
1
2
3

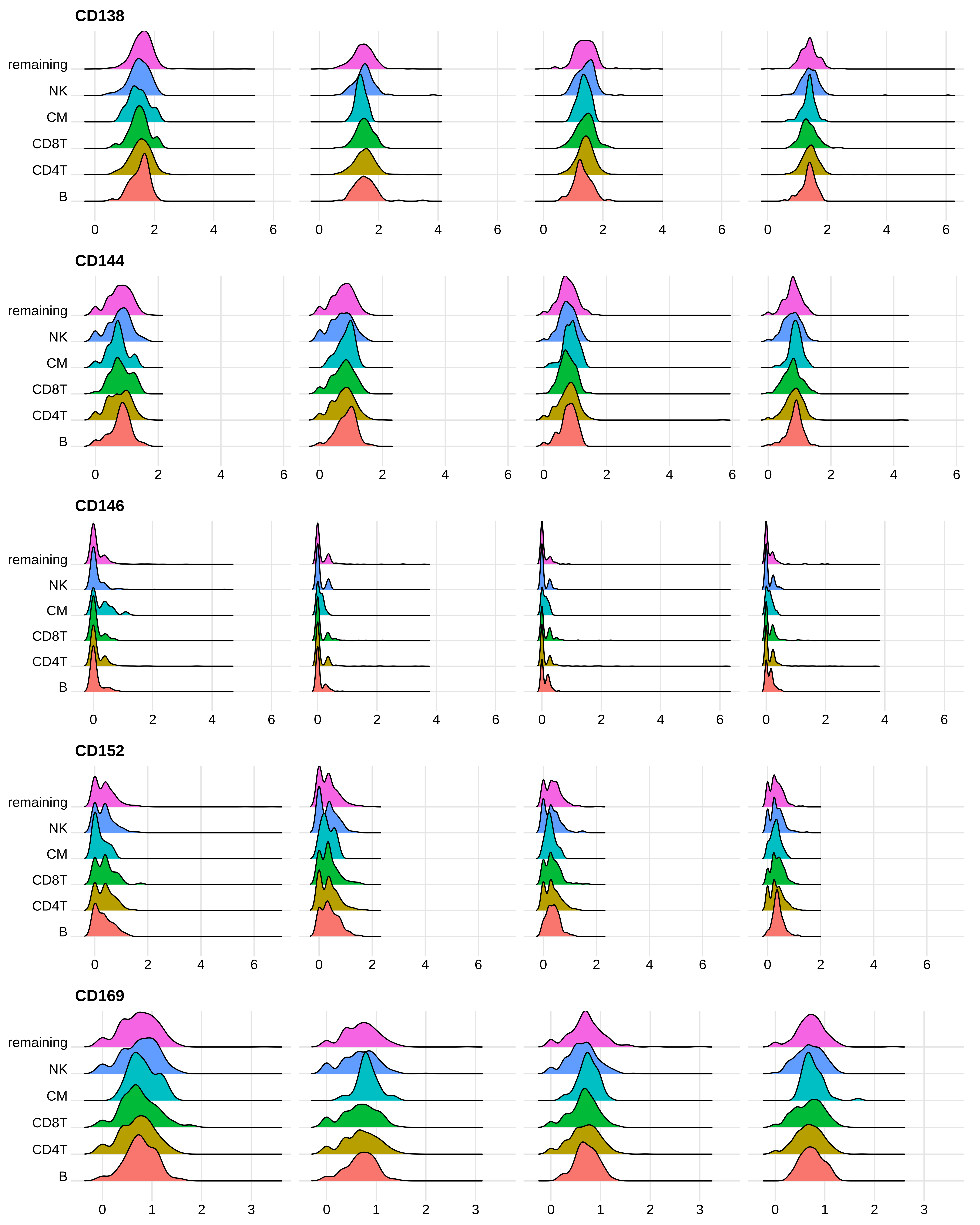

CD138
CD144
CD146
CD152
CD169
remaining
NK
CM
CD8T
CD4T
B
0
1
2
3
4
6

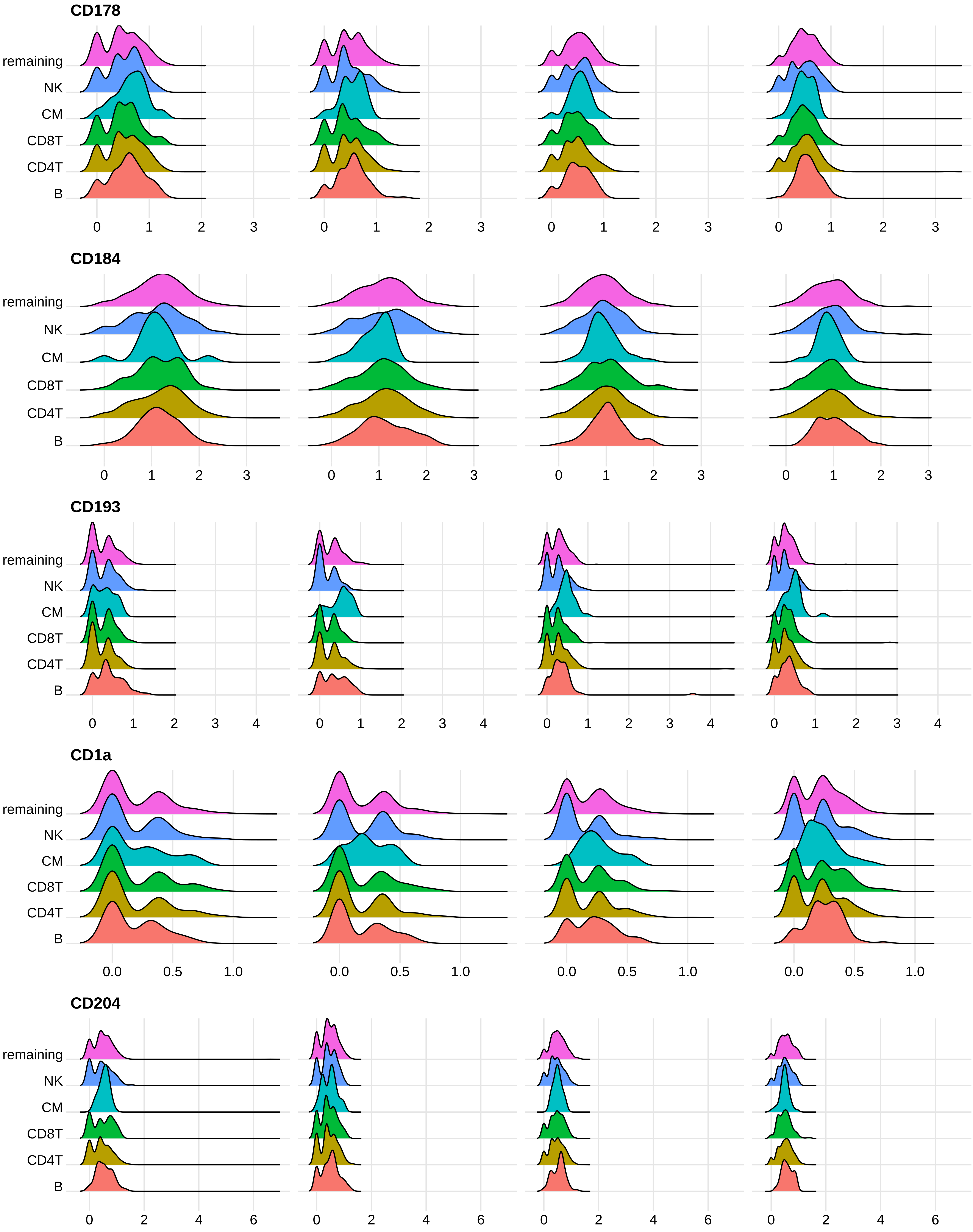
CD178
remaining
NK
CM
CD8T
CD4T
B
0
1
2
3
CD184
CD193
4
CD1a
0.0
0.5
1.0
CD204
6

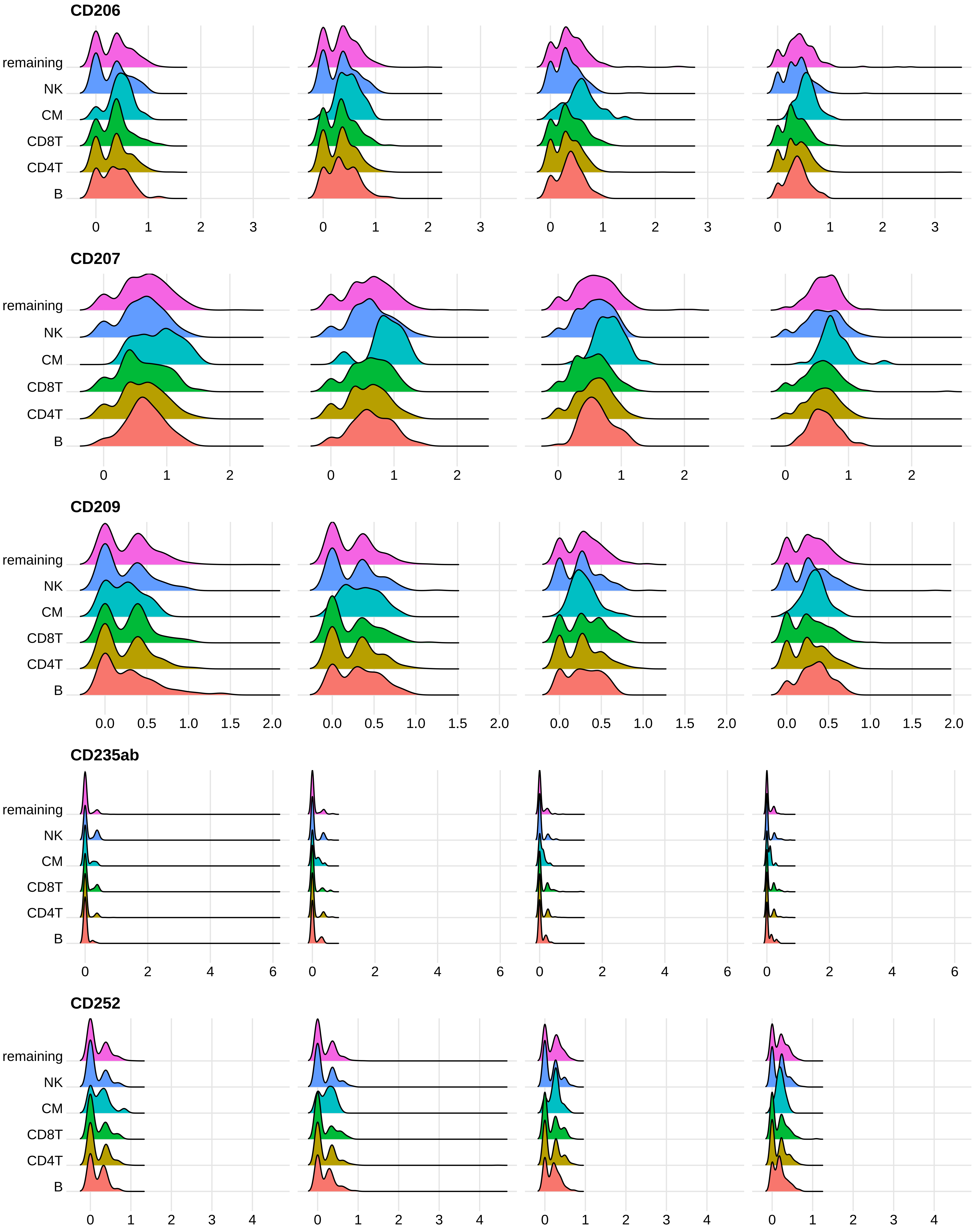
CD206
remaining
NK
CM
CD8T
CD4T
B
0 1 2 3
CD207
remaining
NK
CM
CD8T
CD4T
B
0 1 2
CD209
remaining
NK
CM
CD8T
CD4T
B
0.0 0.5 1.0 1.5 2.0
CD235ab
remaining
NK
CM
CD8T
CD4T
B
0 2 4 6
CD252
remaining
NK
CM
CD8T
CD4T
B
0 1 2 3 4

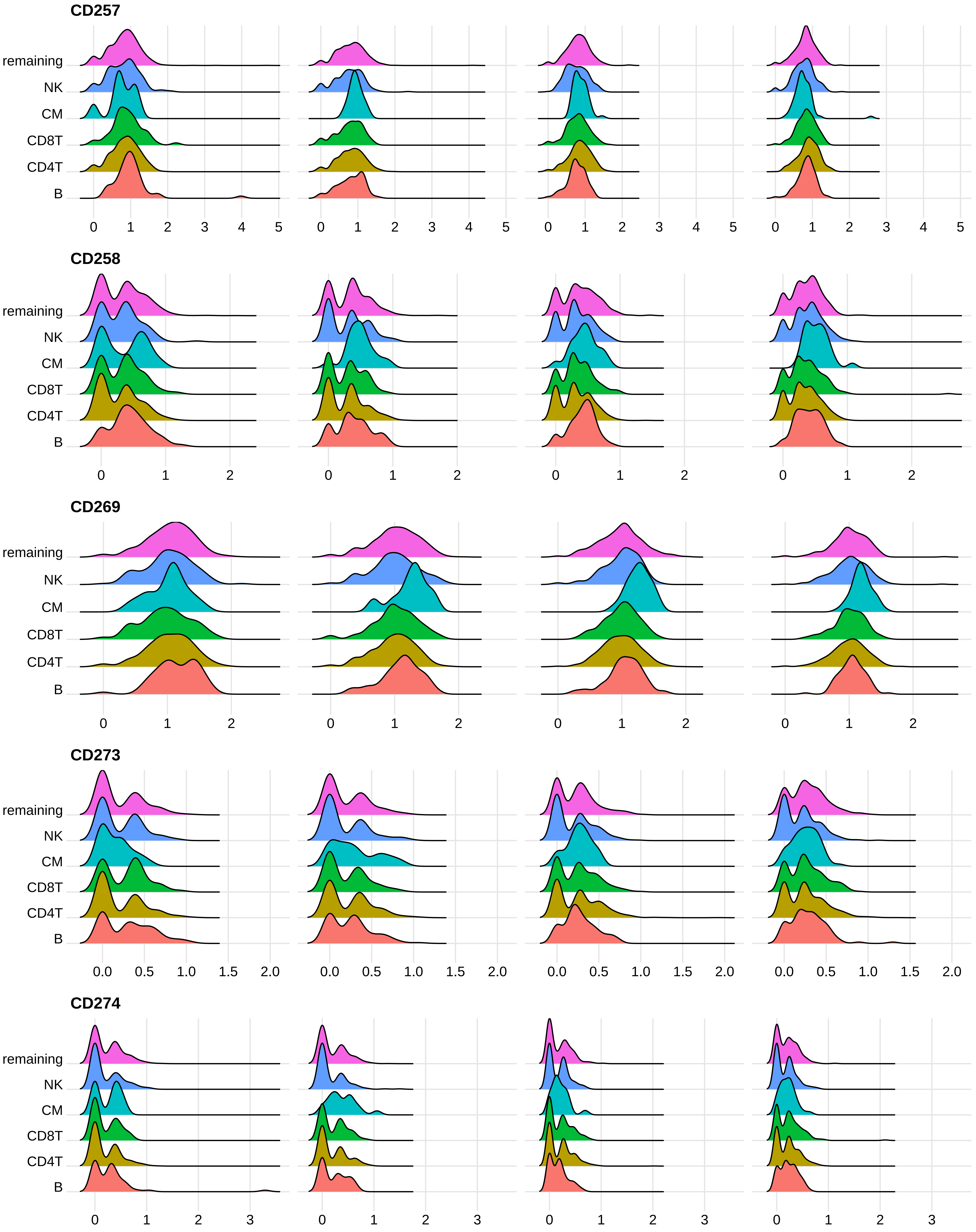
CD257
CD258
CD269
CD273
CD274
remaining
NK
CM
CD8T
CD4T
B
0
1
2
3
4
5
0.0
0.5
1.0
1.5
2.0

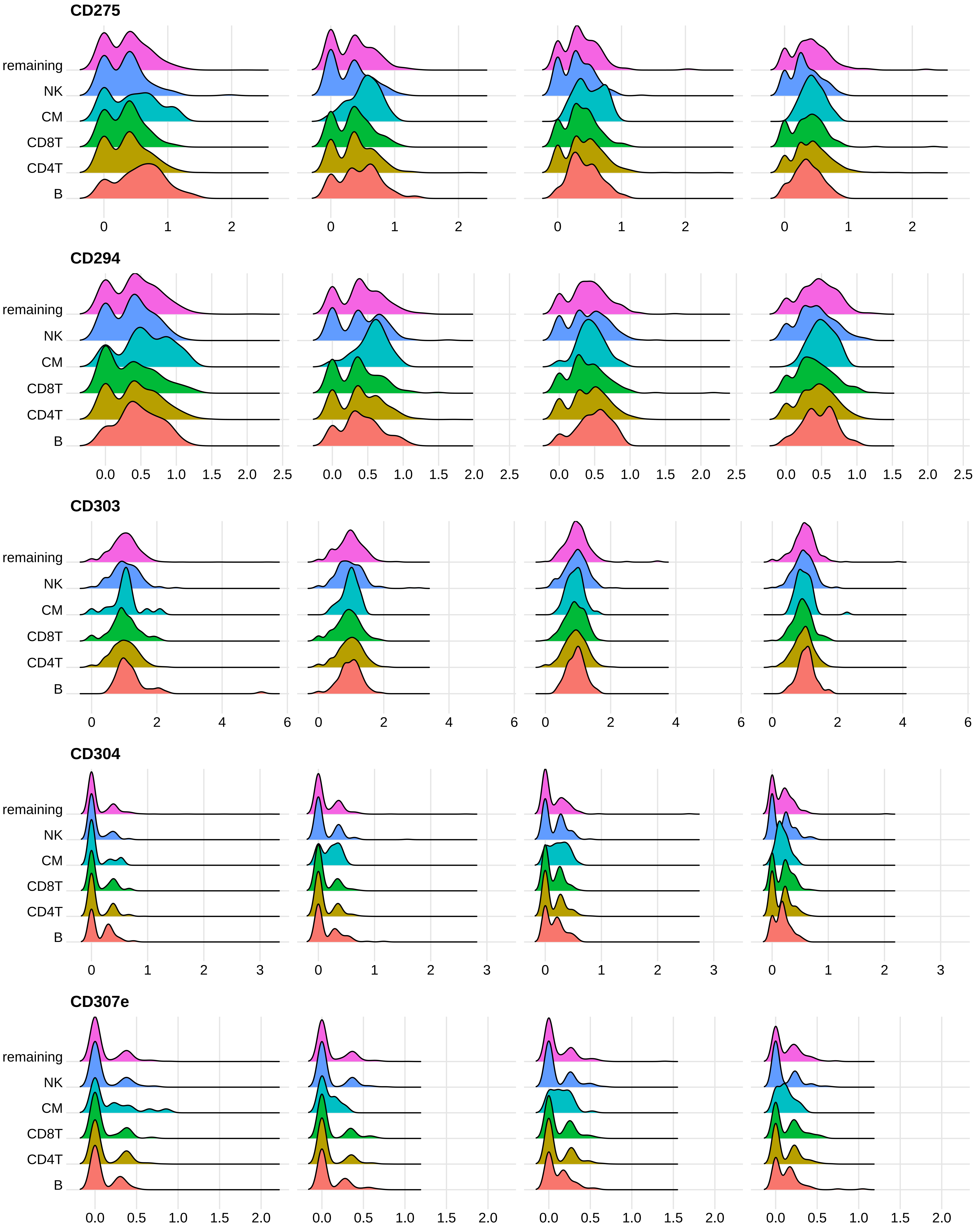
CD275
remaining
NK
CM
CD8T
CD4T
B
0
1
2
CD294
0.0
0.5
1.0
1.5
2.0
2.5
CD303
4
6
CD304
3
CD307e

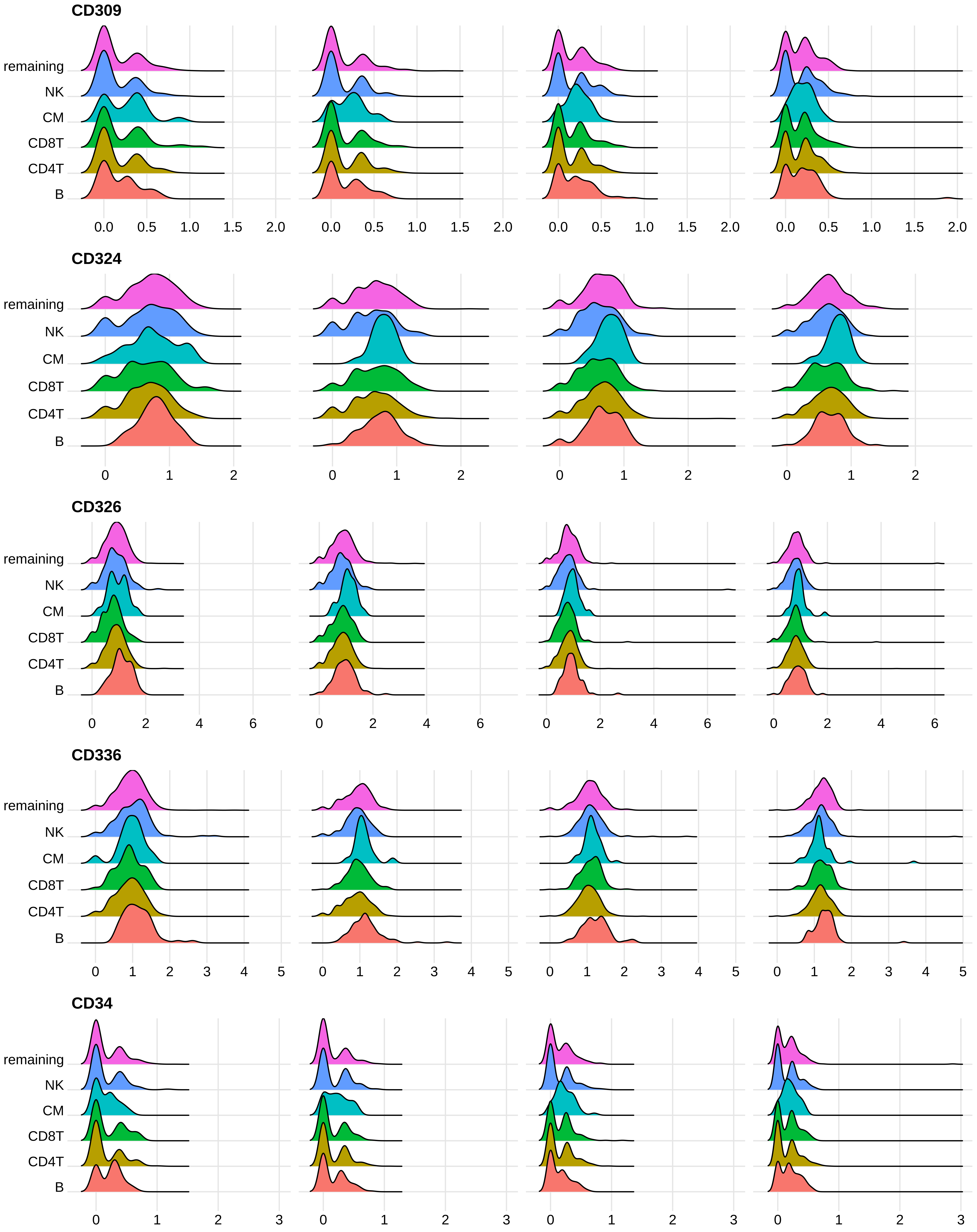
CD309
CD324
CD326
CD336
CD34
remaining
NK
CM
CD8T
CD4T
B
0.0 0.5 1.0 1.5 2.0
0 1 2
0 2 4 6
0 1 2 3 4 5
0 1 2 3

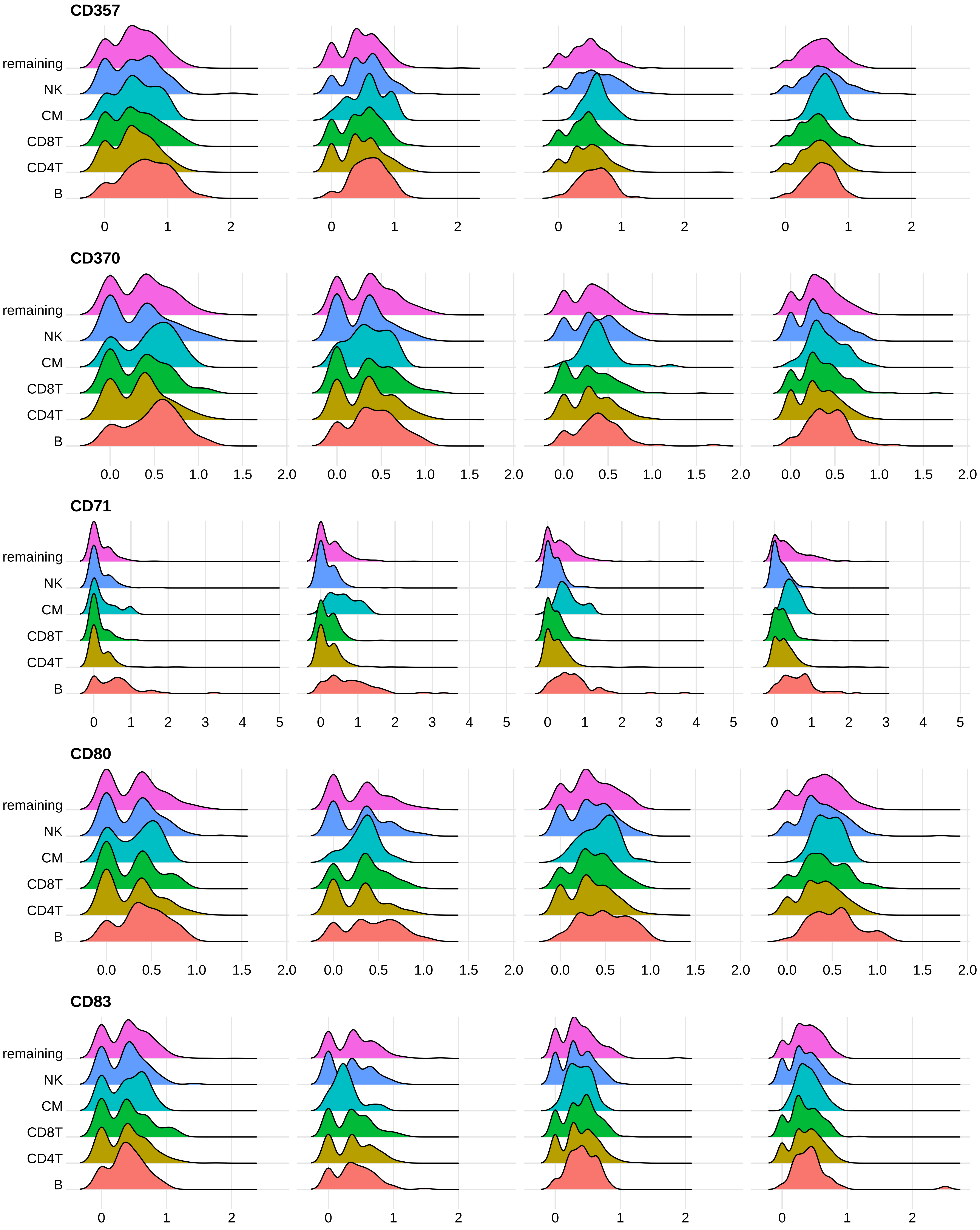
CD357
CD370
CD71
CD80
CD83
remaining
NK
CM
CD8T
CD4T
B

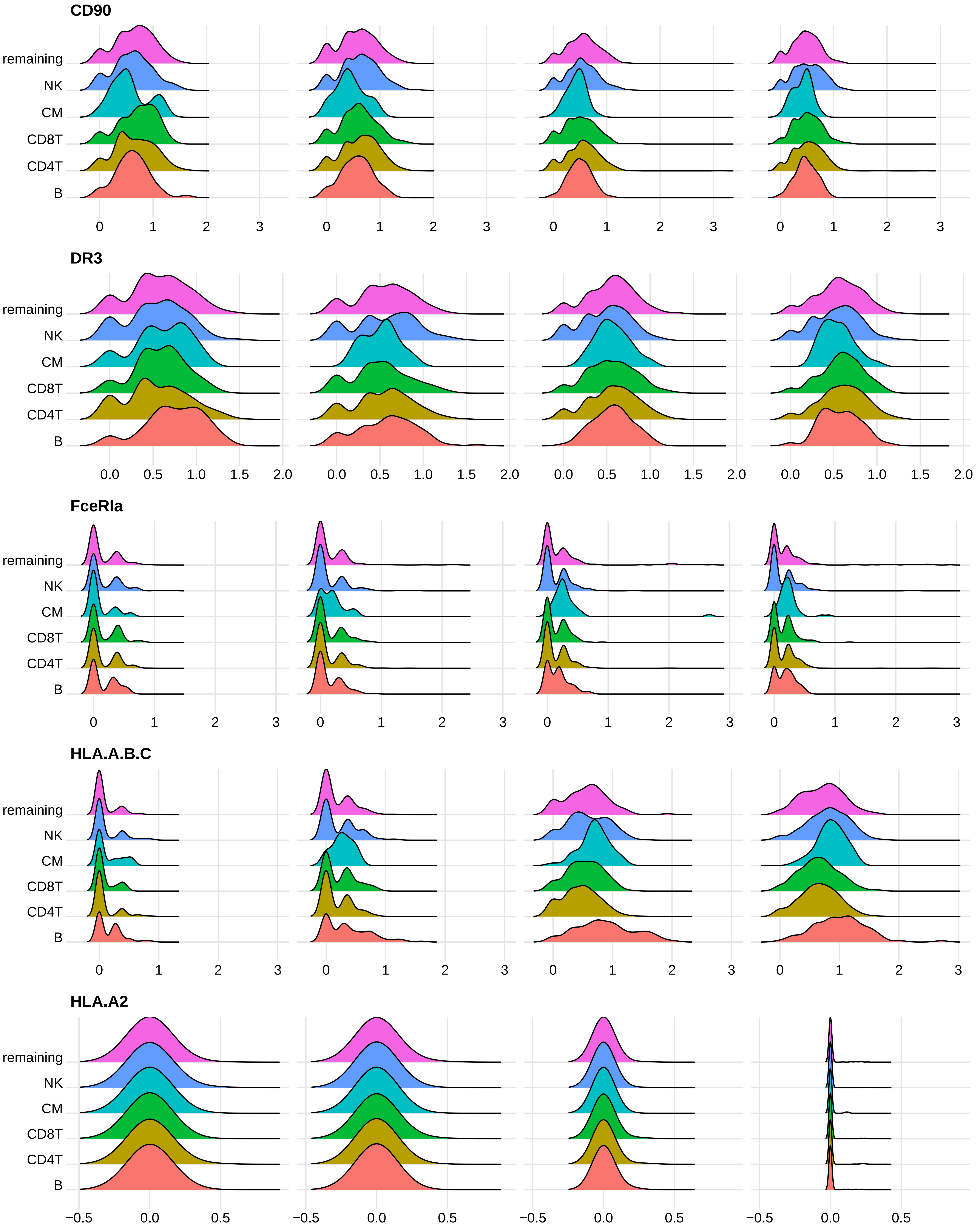

CD90
DR3
FceRIa
HLA.A.B.C
HLA.A2
remaining
NK
CM
CD8T
CD4T
B
0 1 2 3
0.0 0.5 1.0 1.5 2.0
−0.5 0.0 0.5

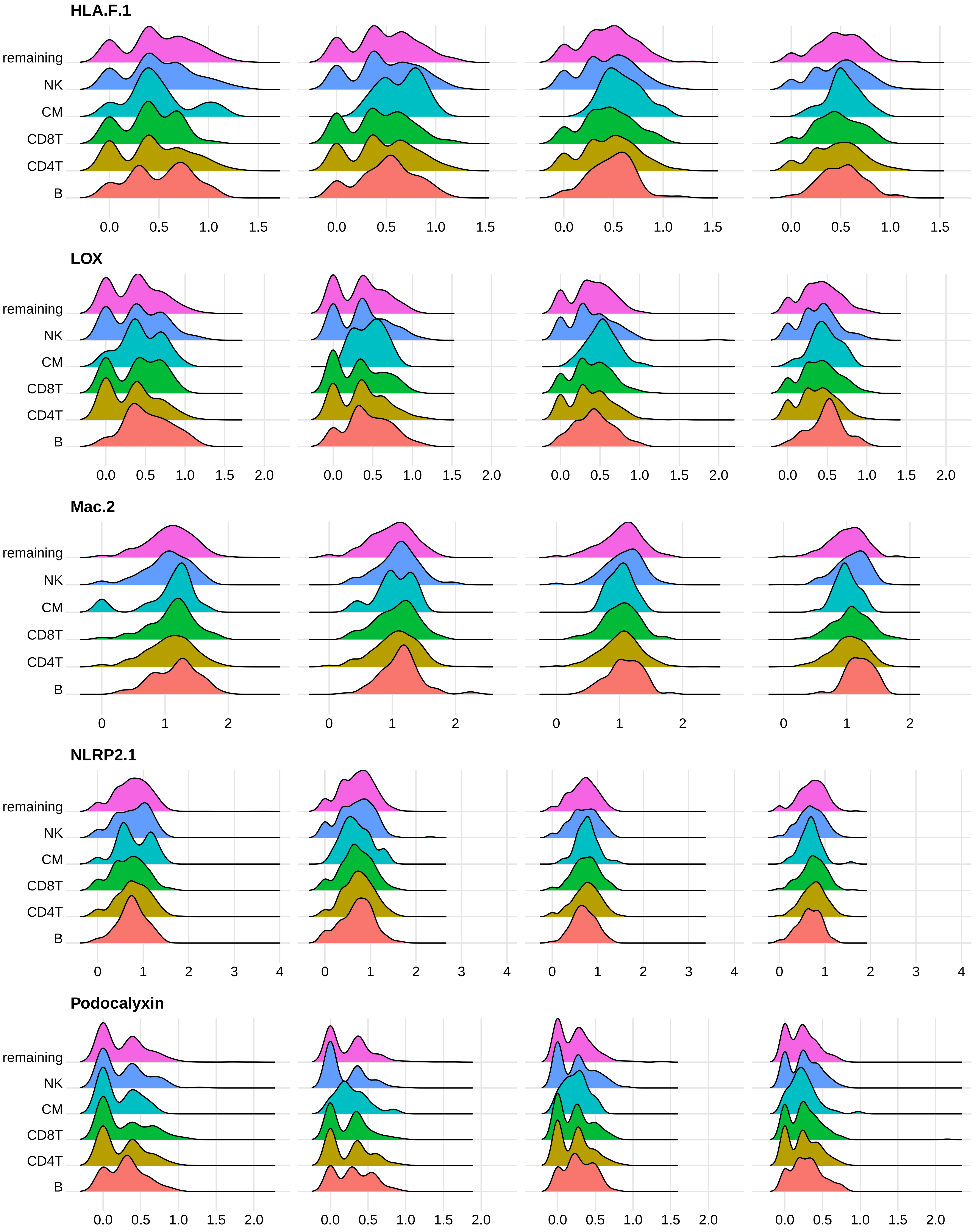

HLA.F.1
LOX
Mac.2
NLRP2.1
Podocalyxin
remaining
NK
CM
CD8T
CD4T
B

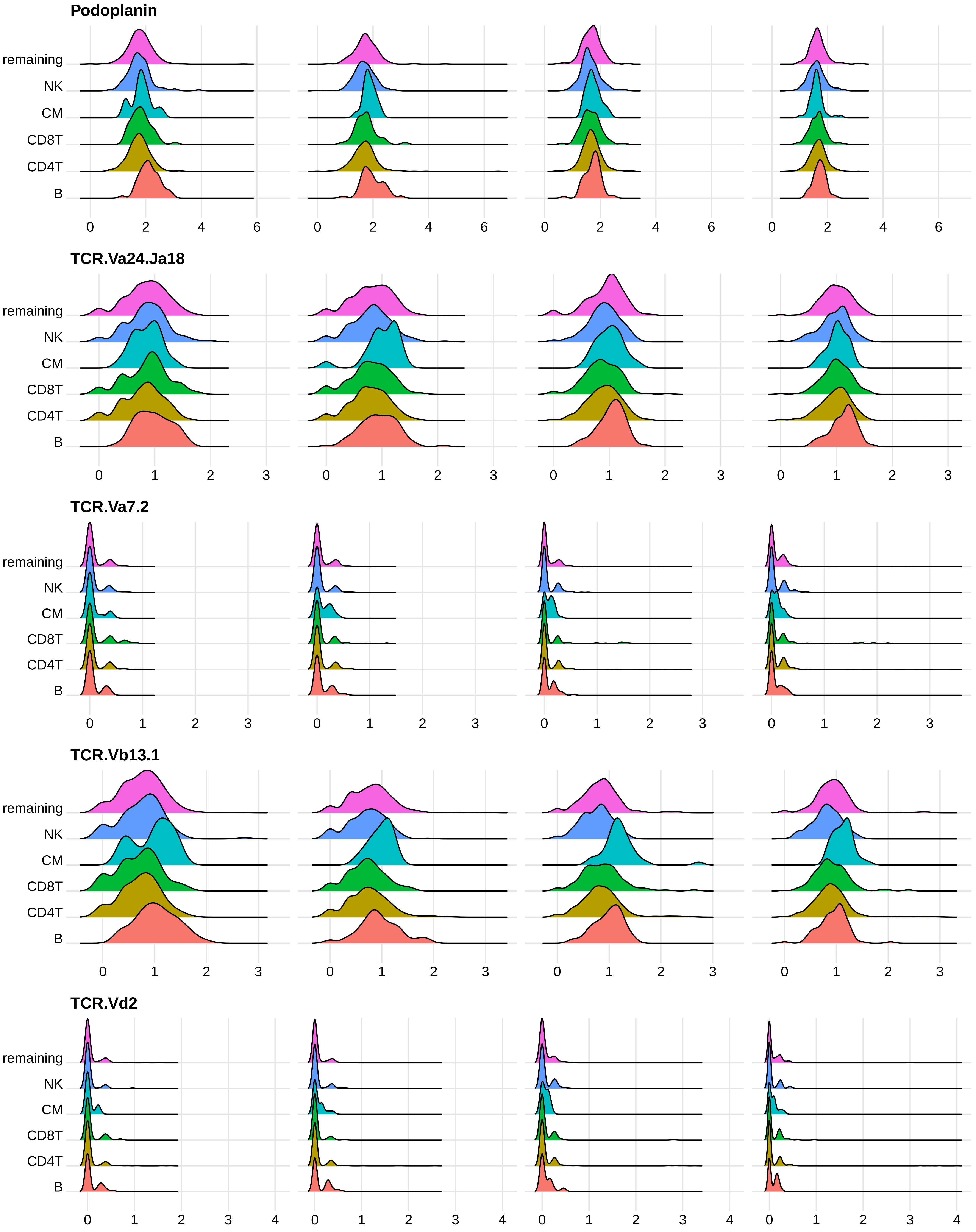

Podoplanin
remaining
NK
CM
CD8T
CD4T
B
0
2
4
6
TCR.Va24.Ja18
0
1
2
3
TCR.Va7.2
TCR.Vb13.1
TCR.Vd2

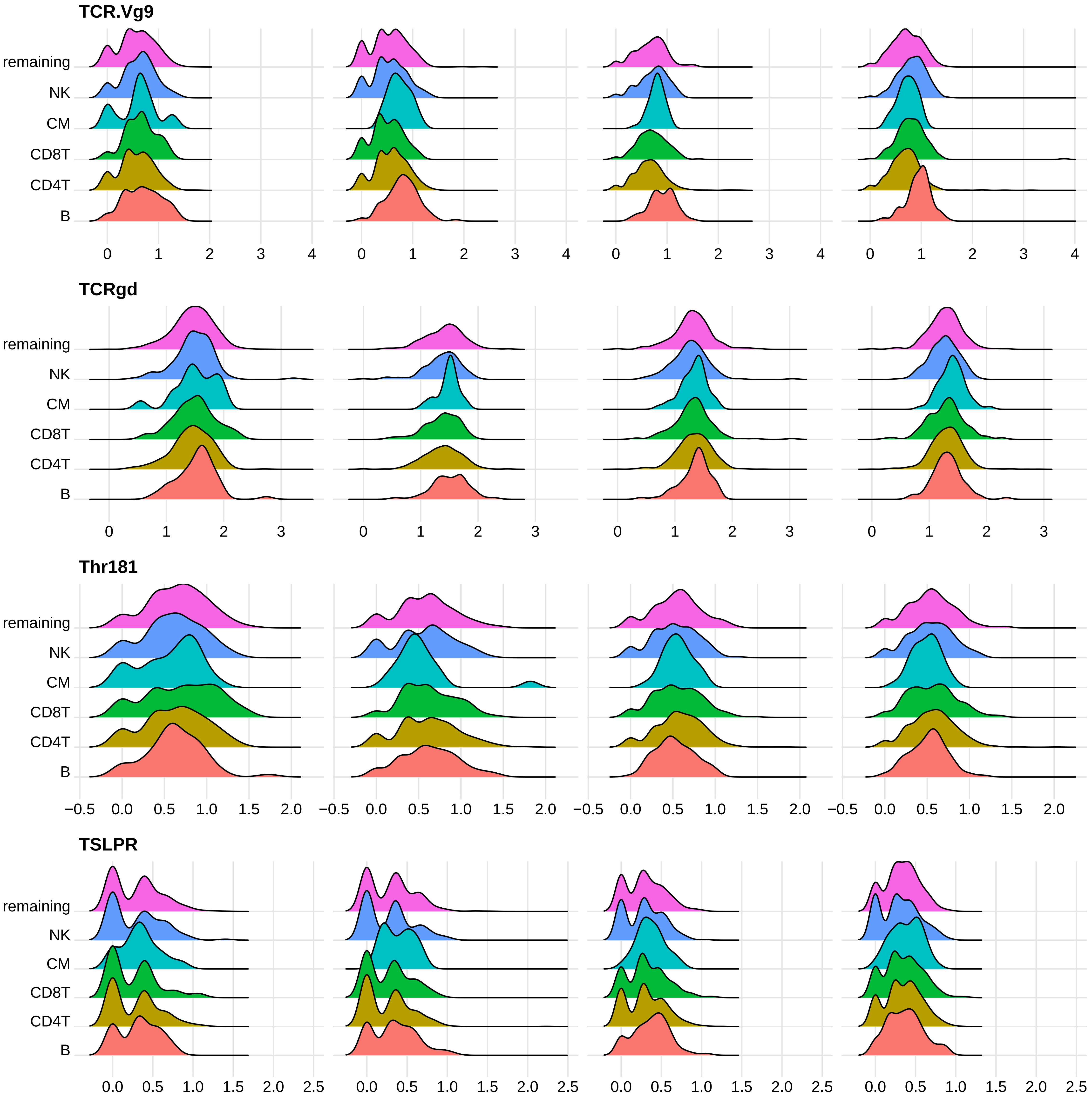

TCR.Vg9
TCRgd
Thr181
TSLPR
remaining
NK
CM
CD8T
CD4T
B
