# Supplementary figures and images for: Titration of 124 antibodies using CITE-Seq on human PBMCs
Source: Sci Rep. 2022 Dec 2;12:20817. doi: 10.1038/s41598-022-24371-7 (PMC9718773; doi:10.1038/s41598-022-24371-7)

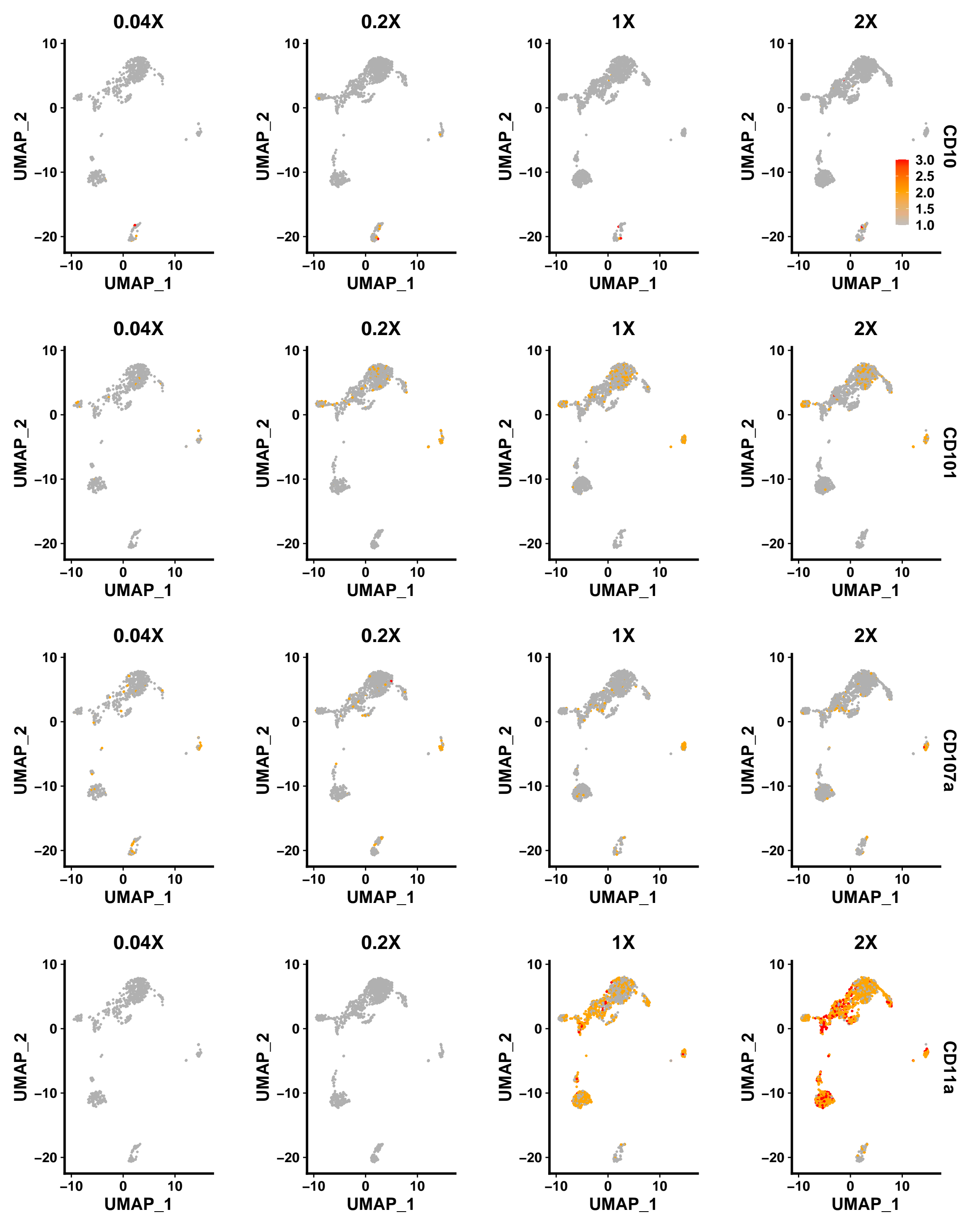

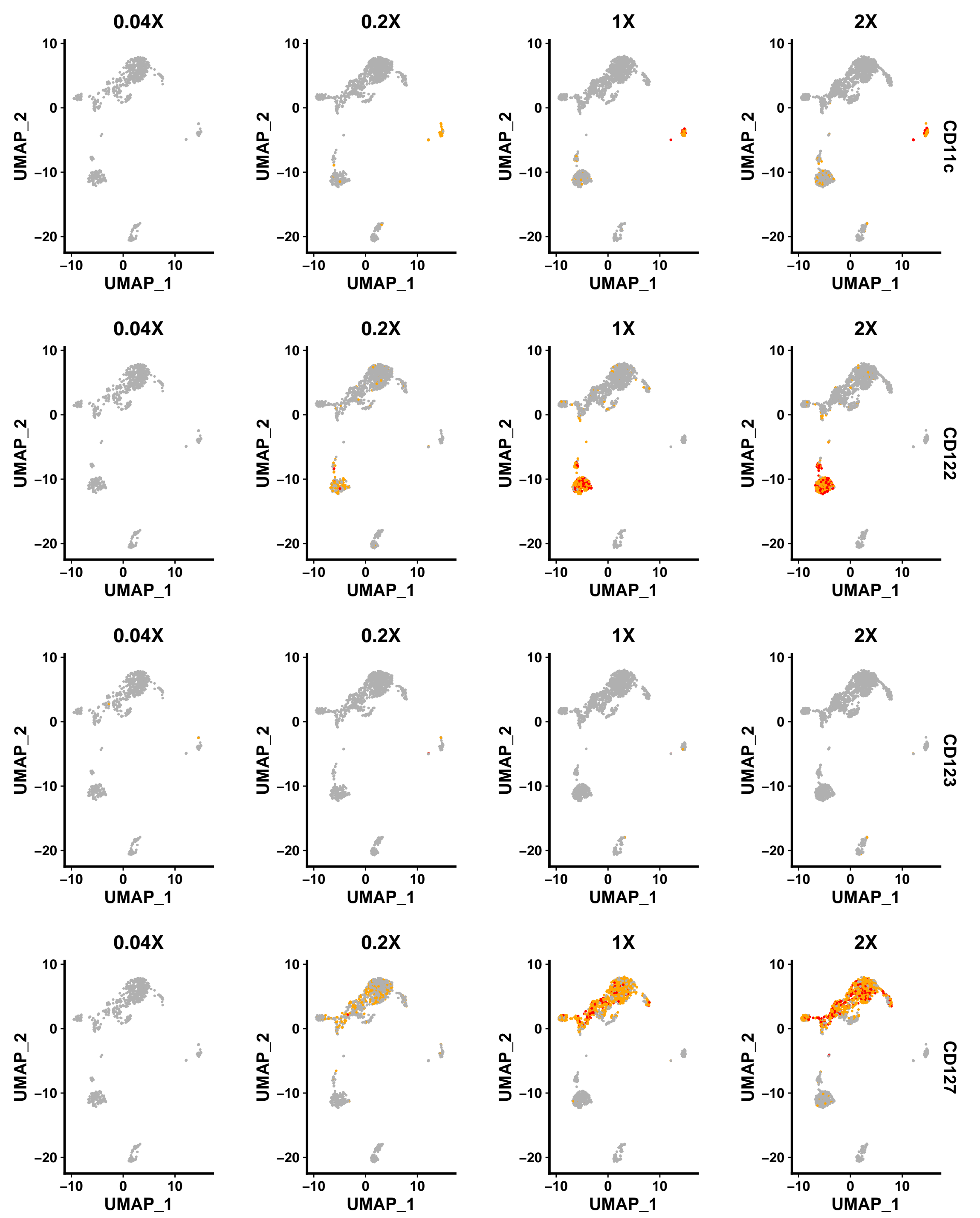

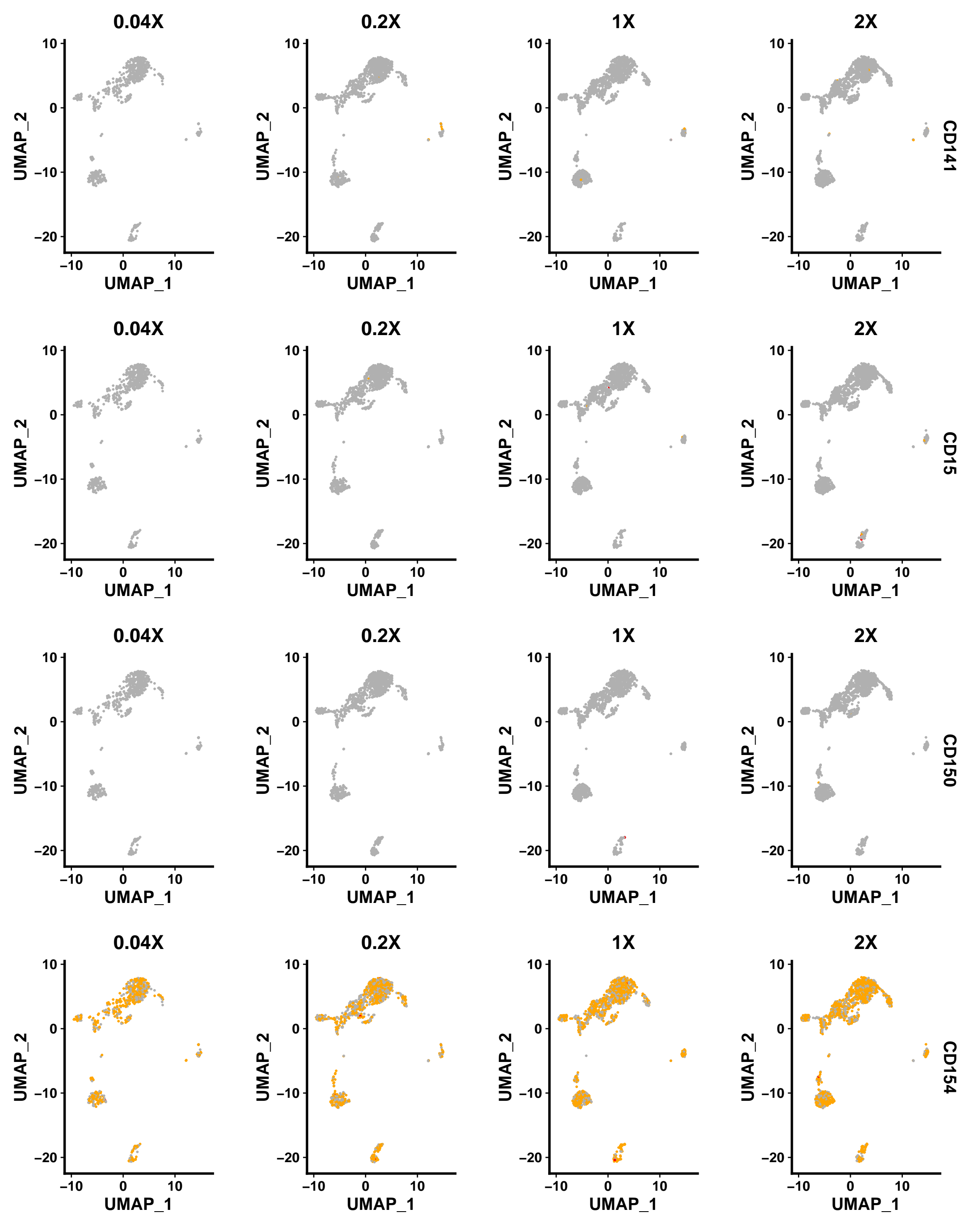

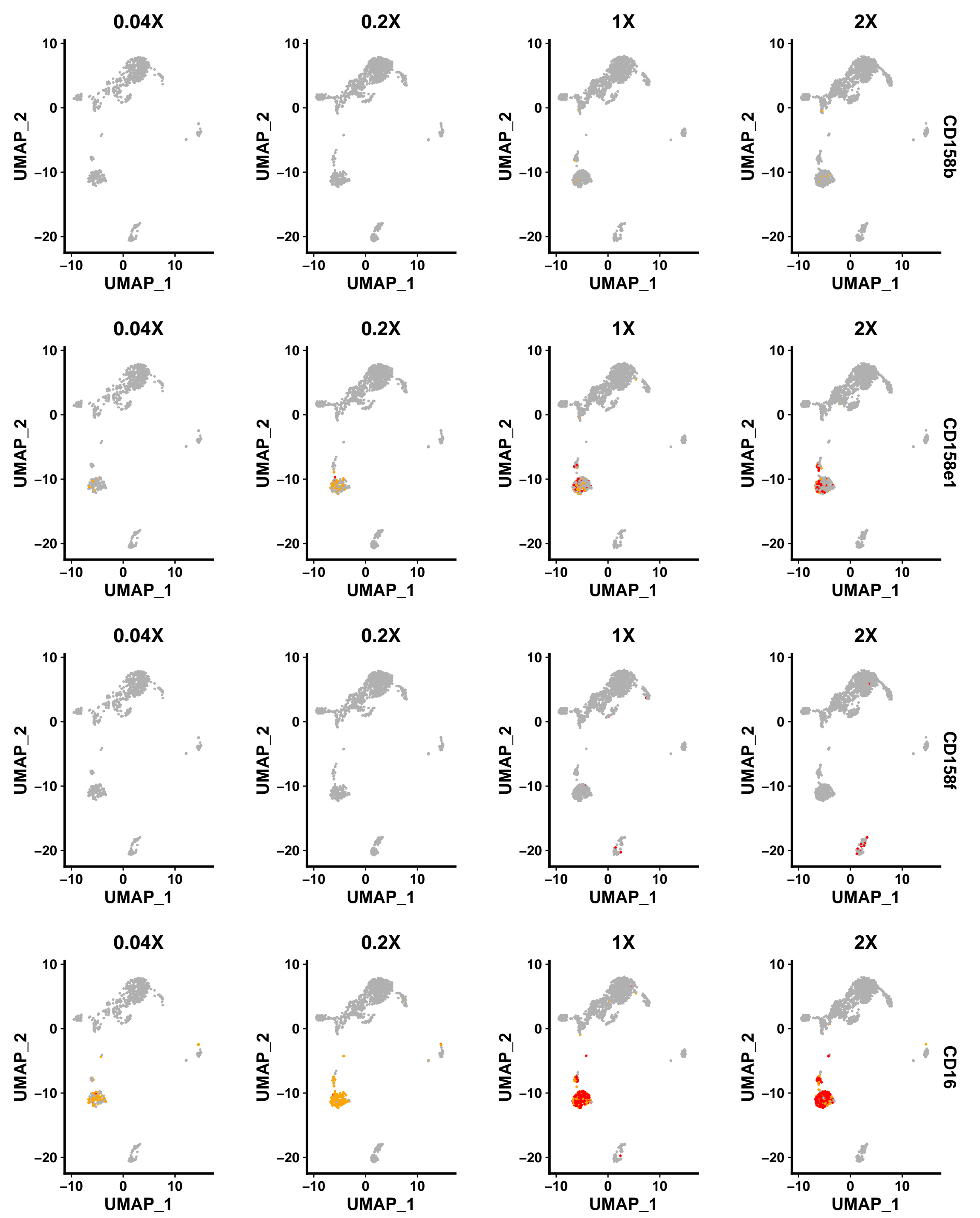

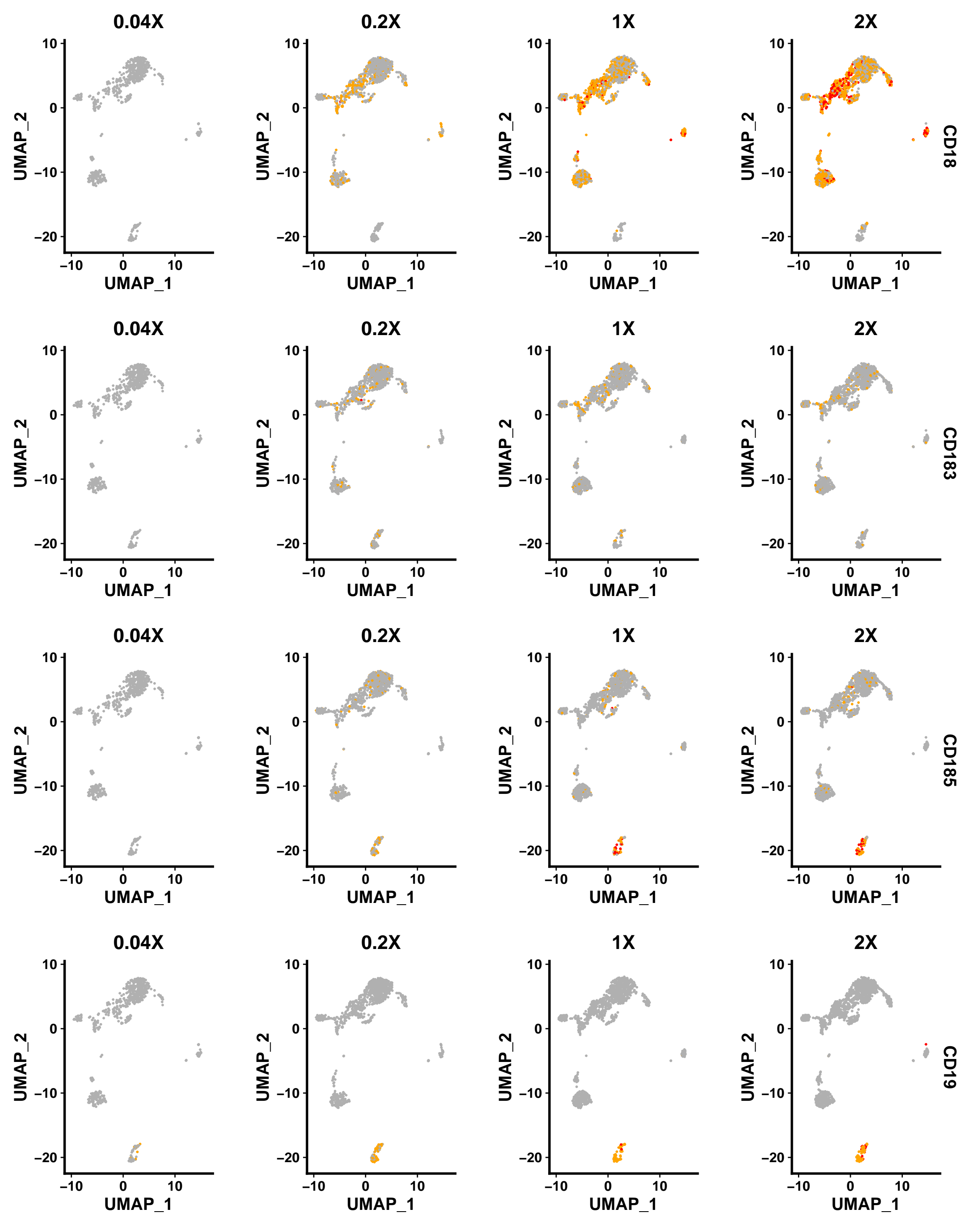

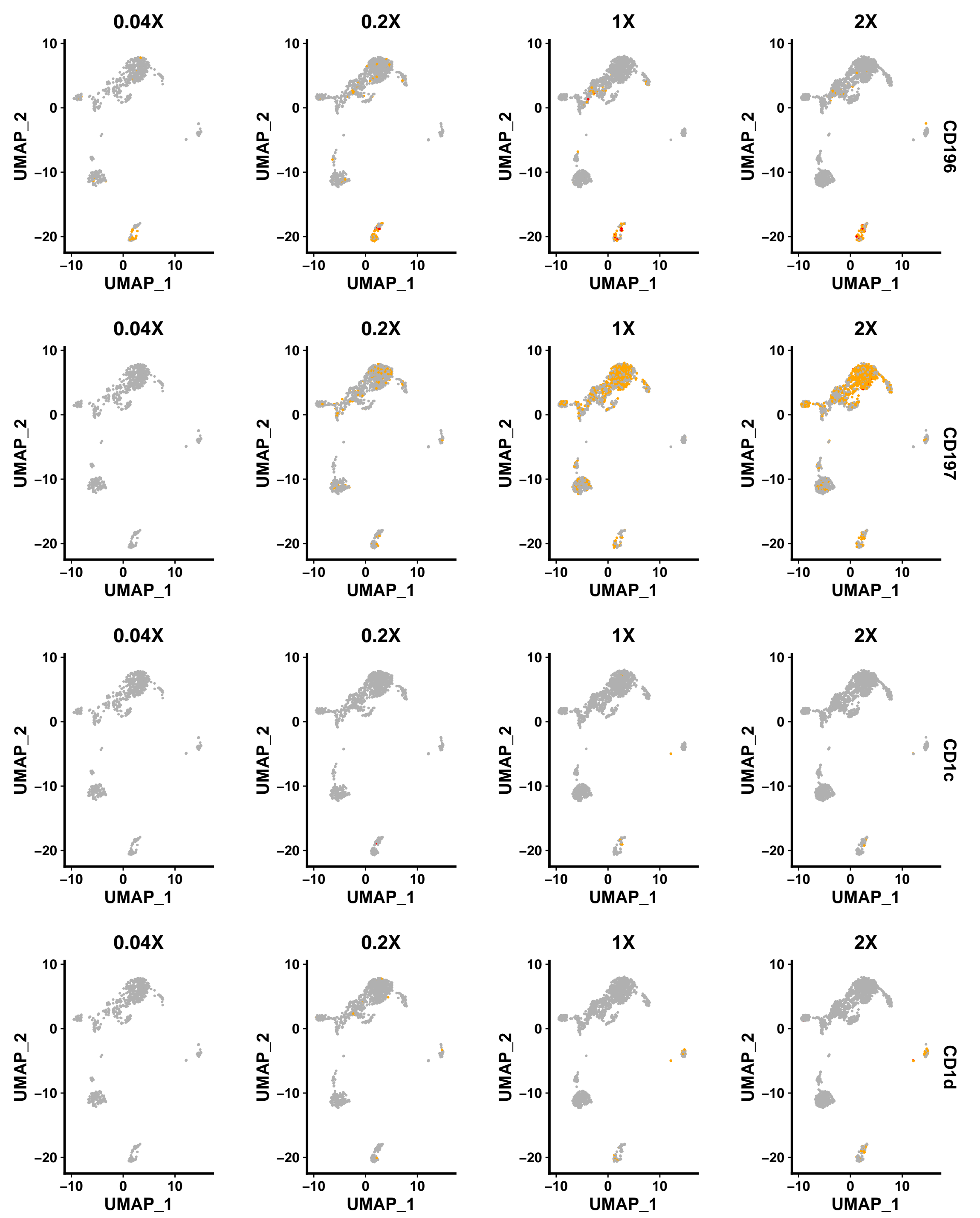

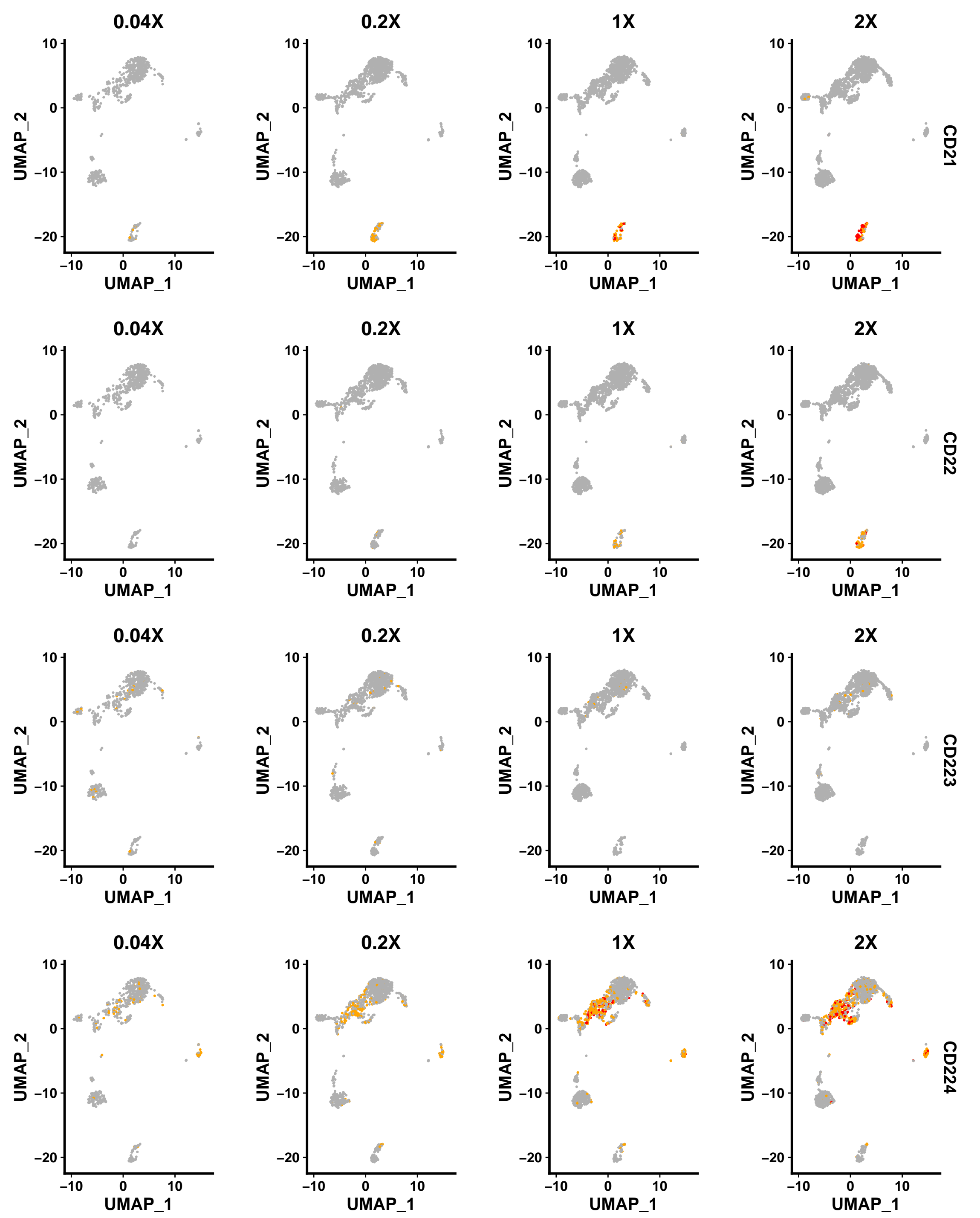

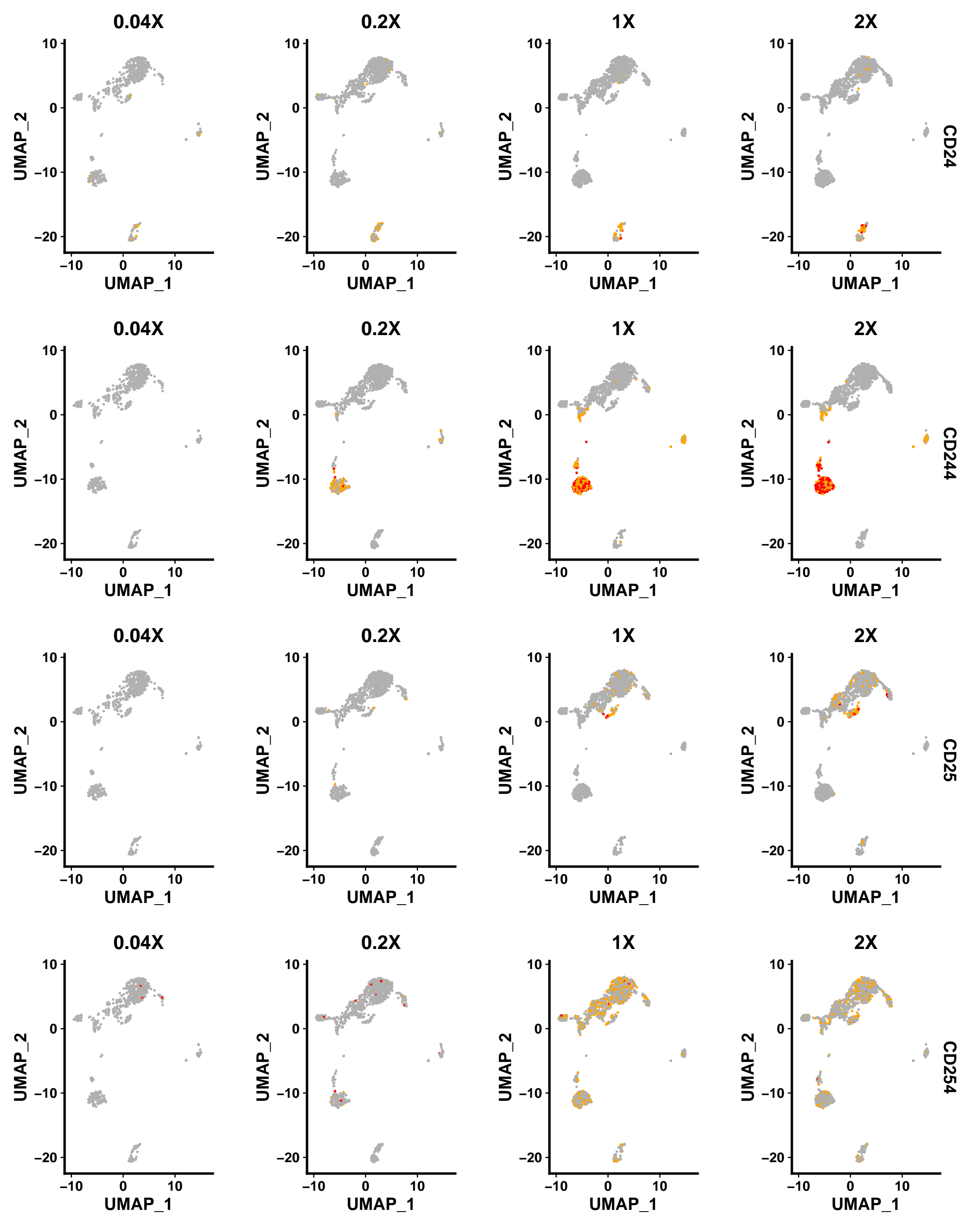

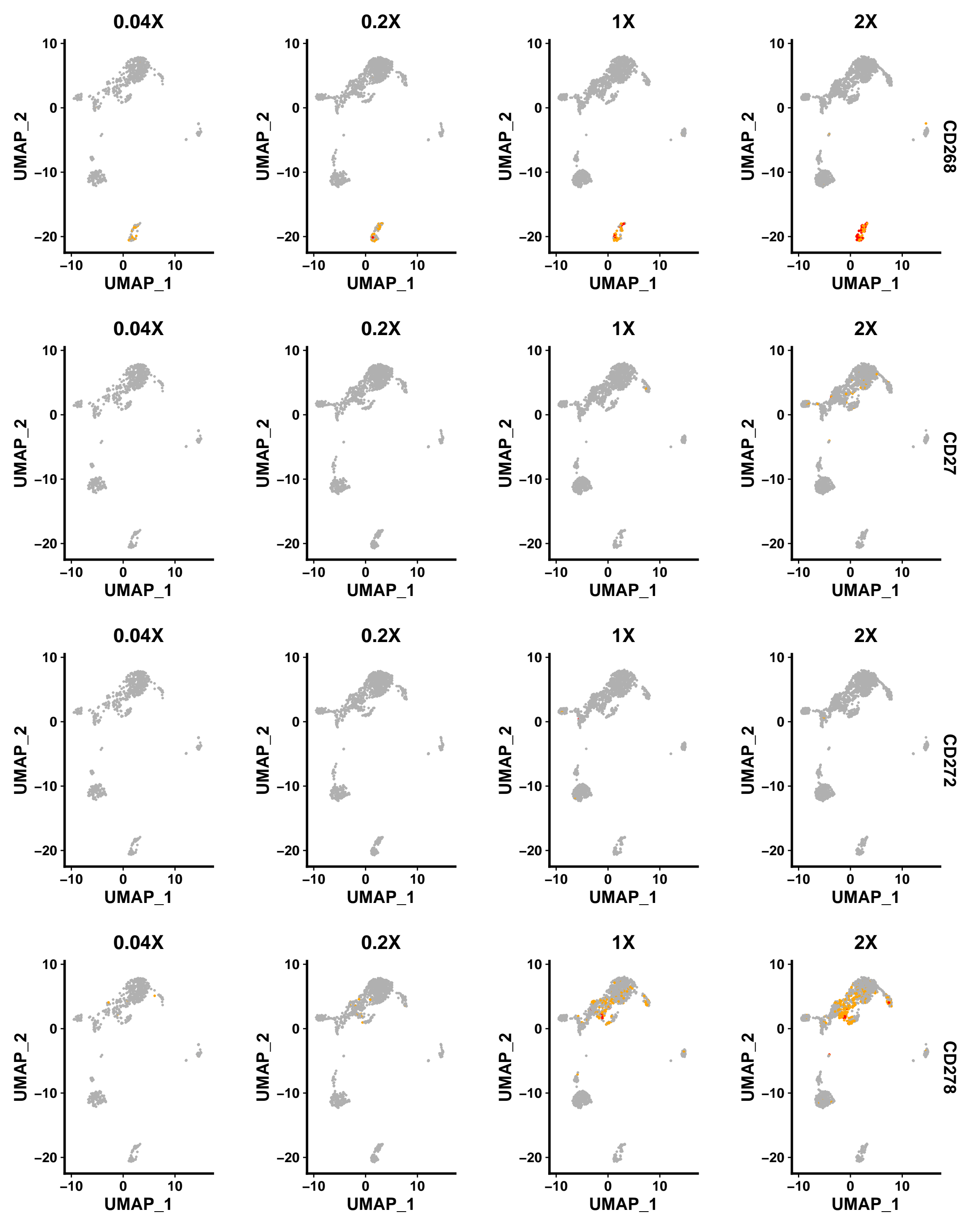

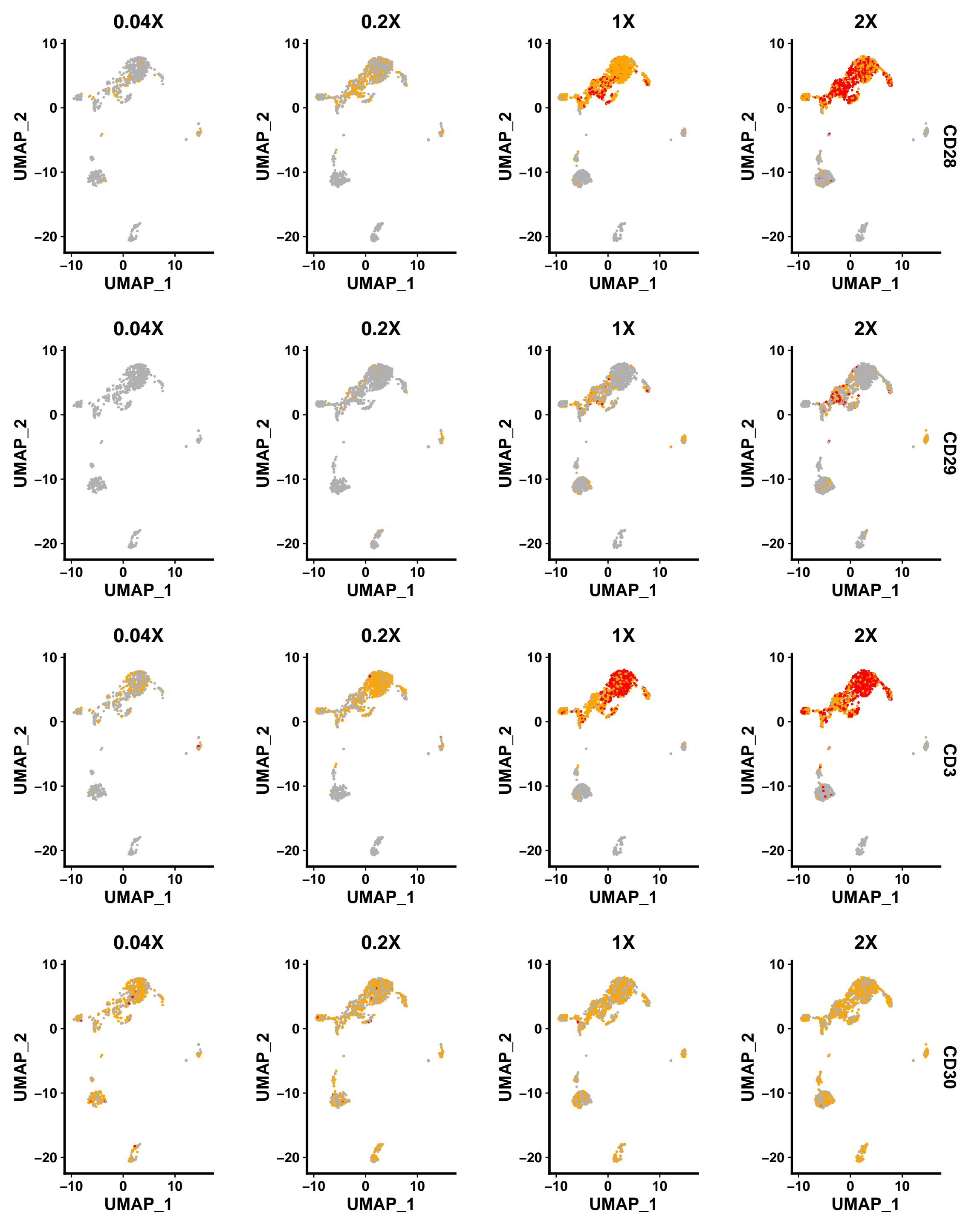

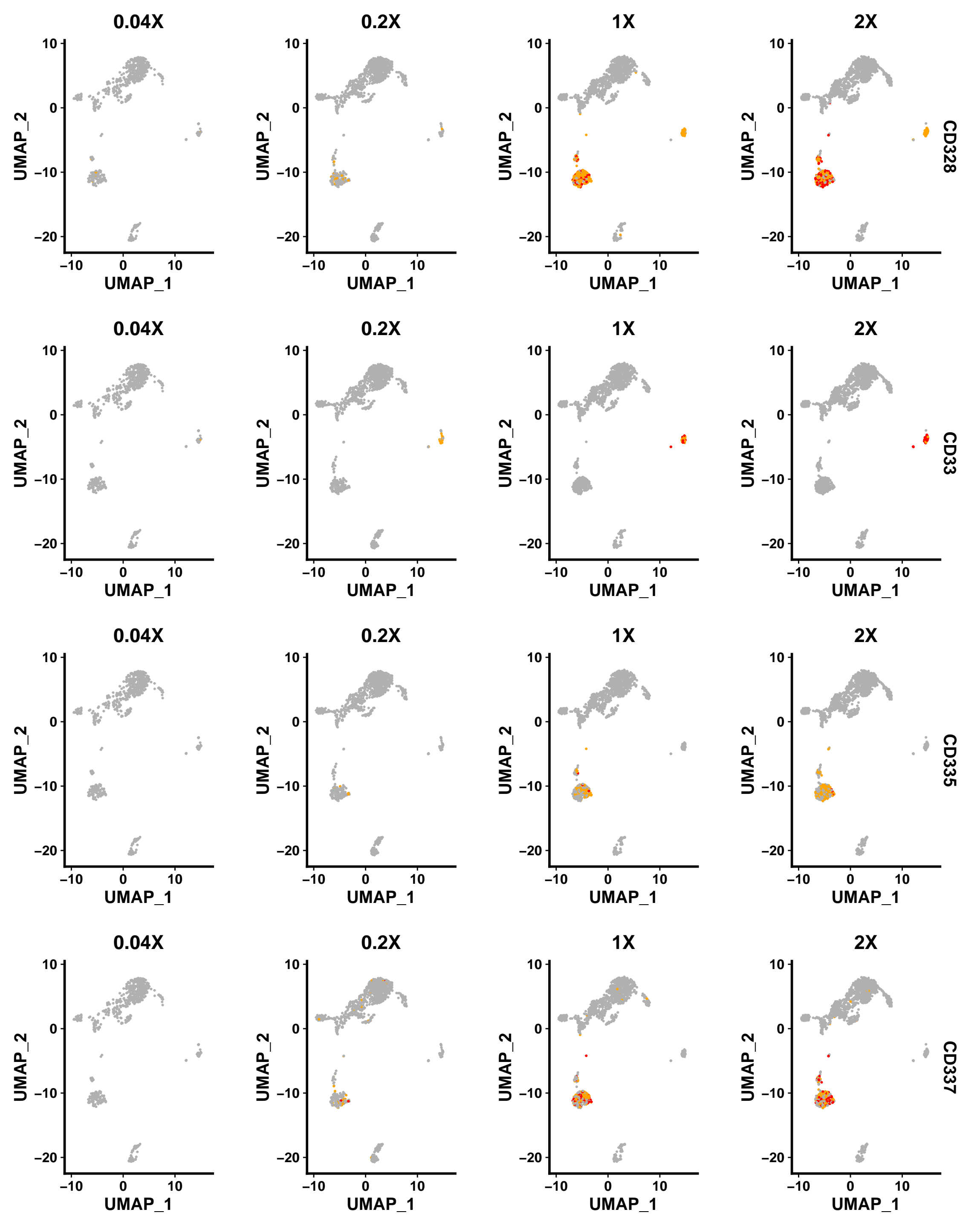

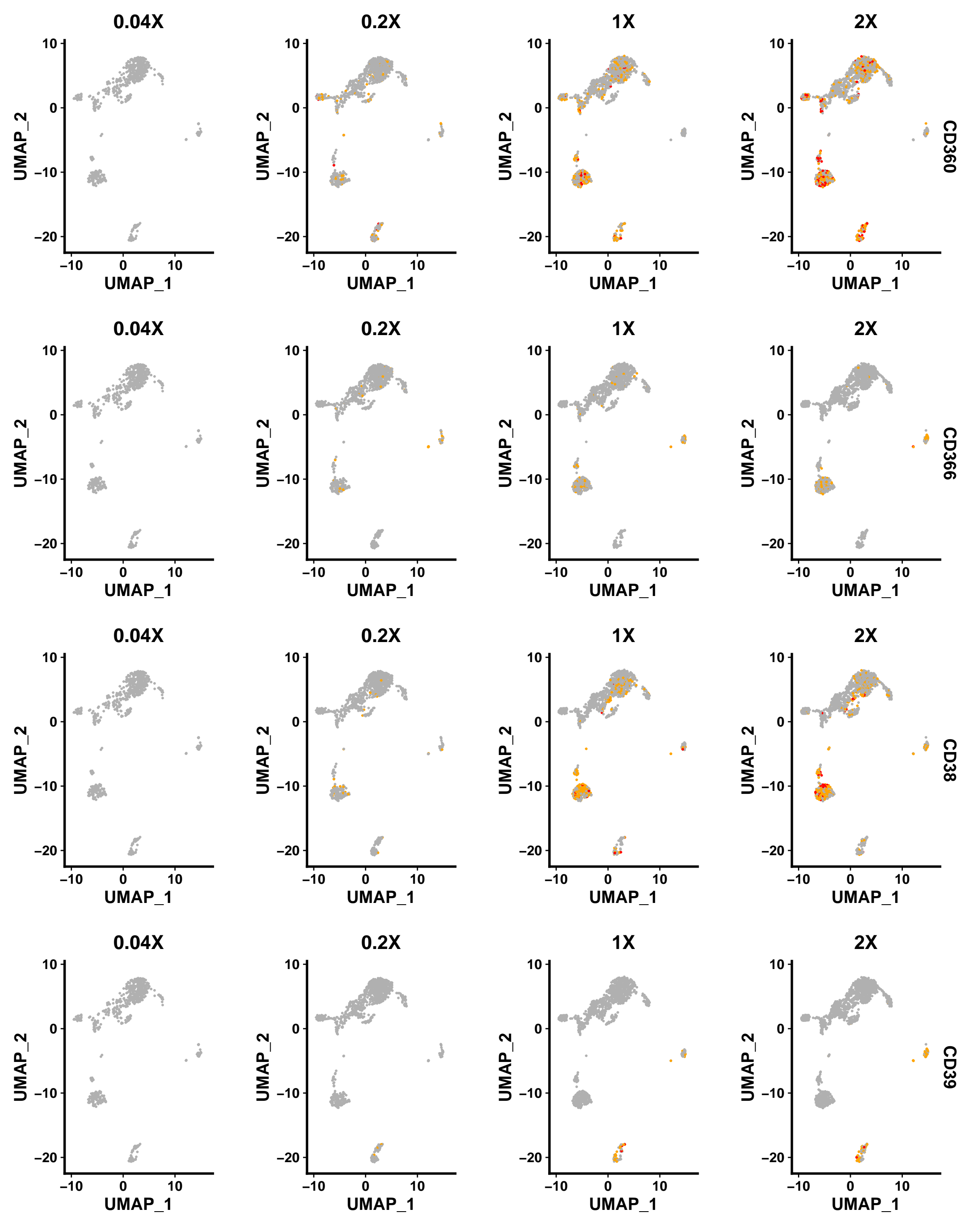

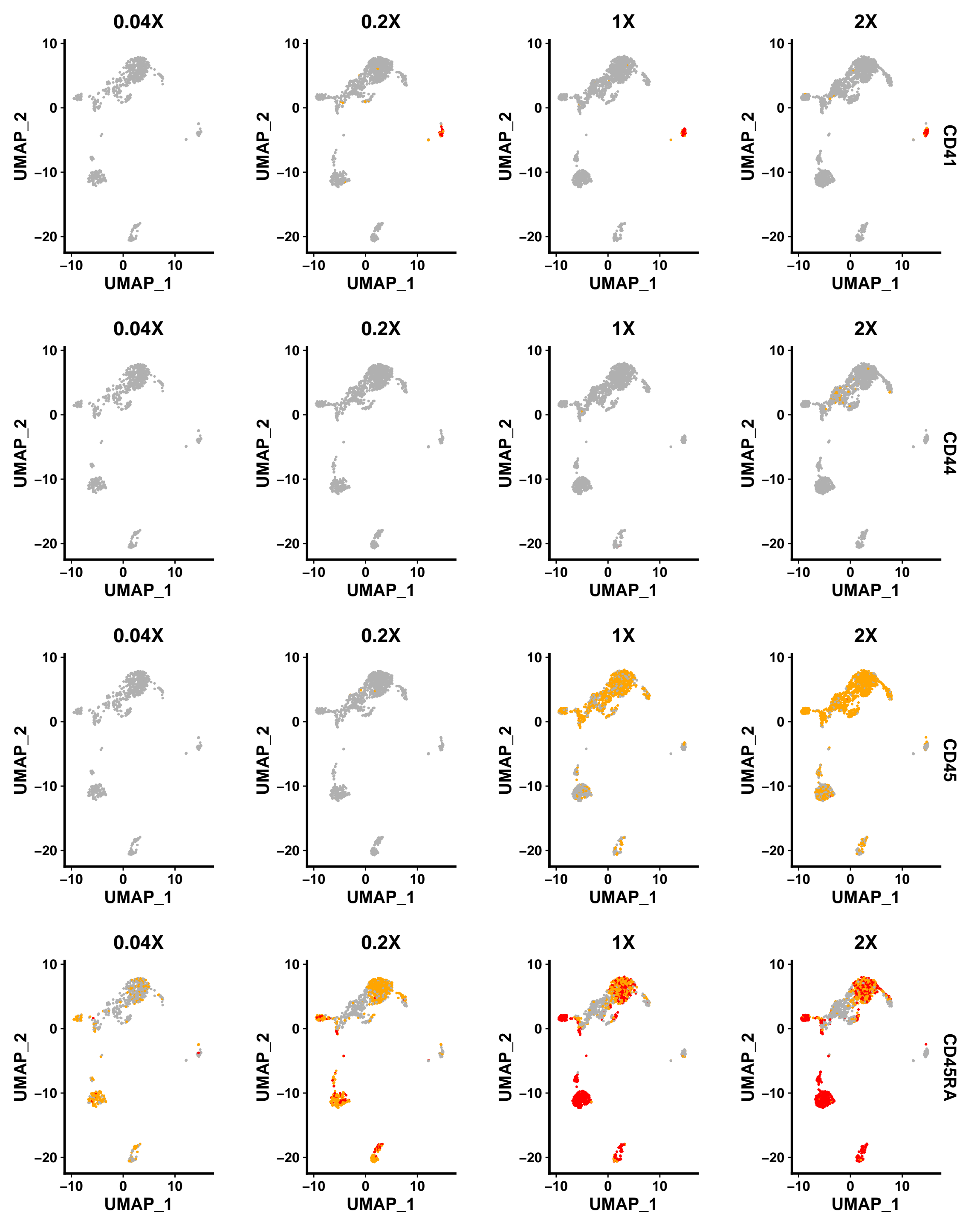

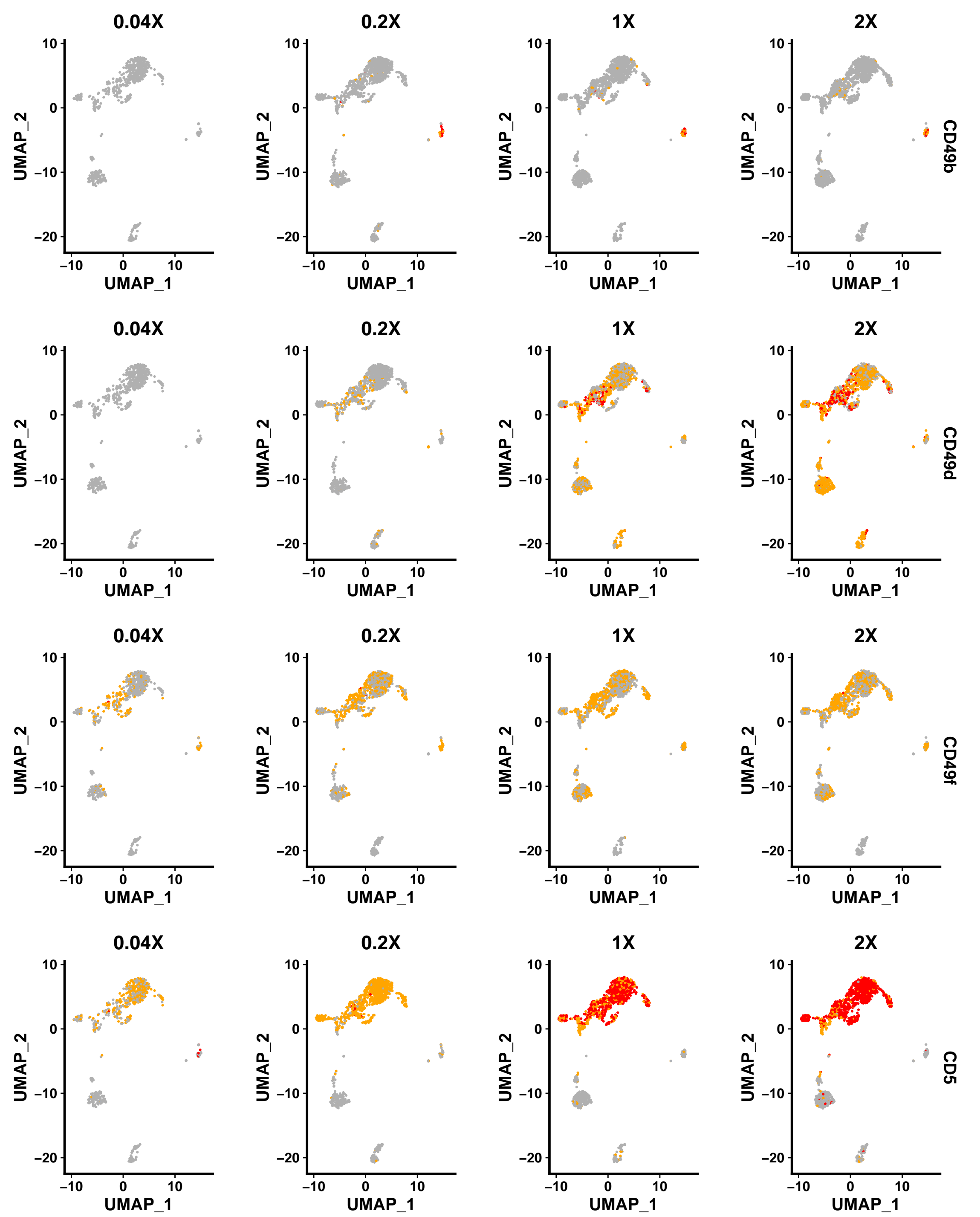

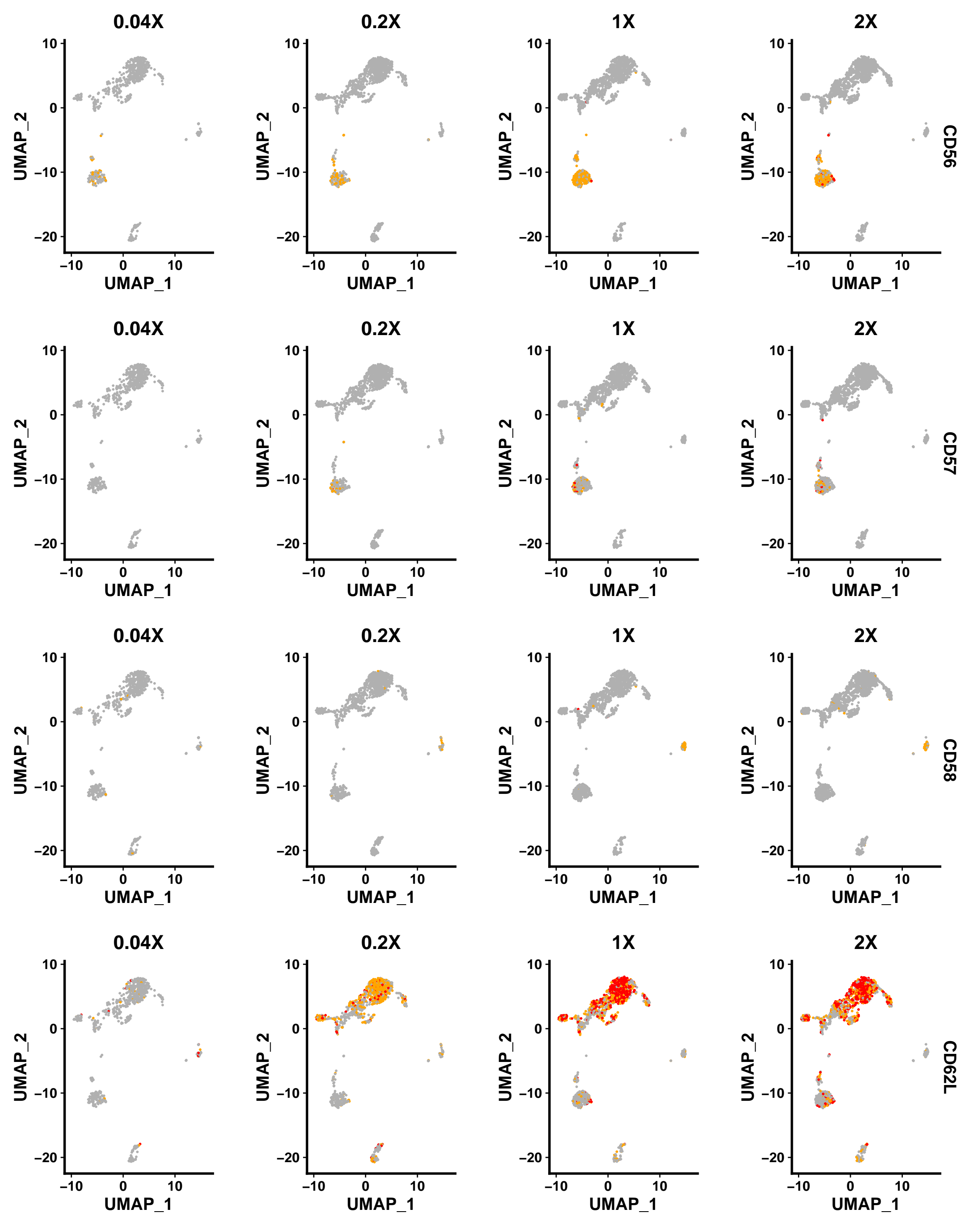

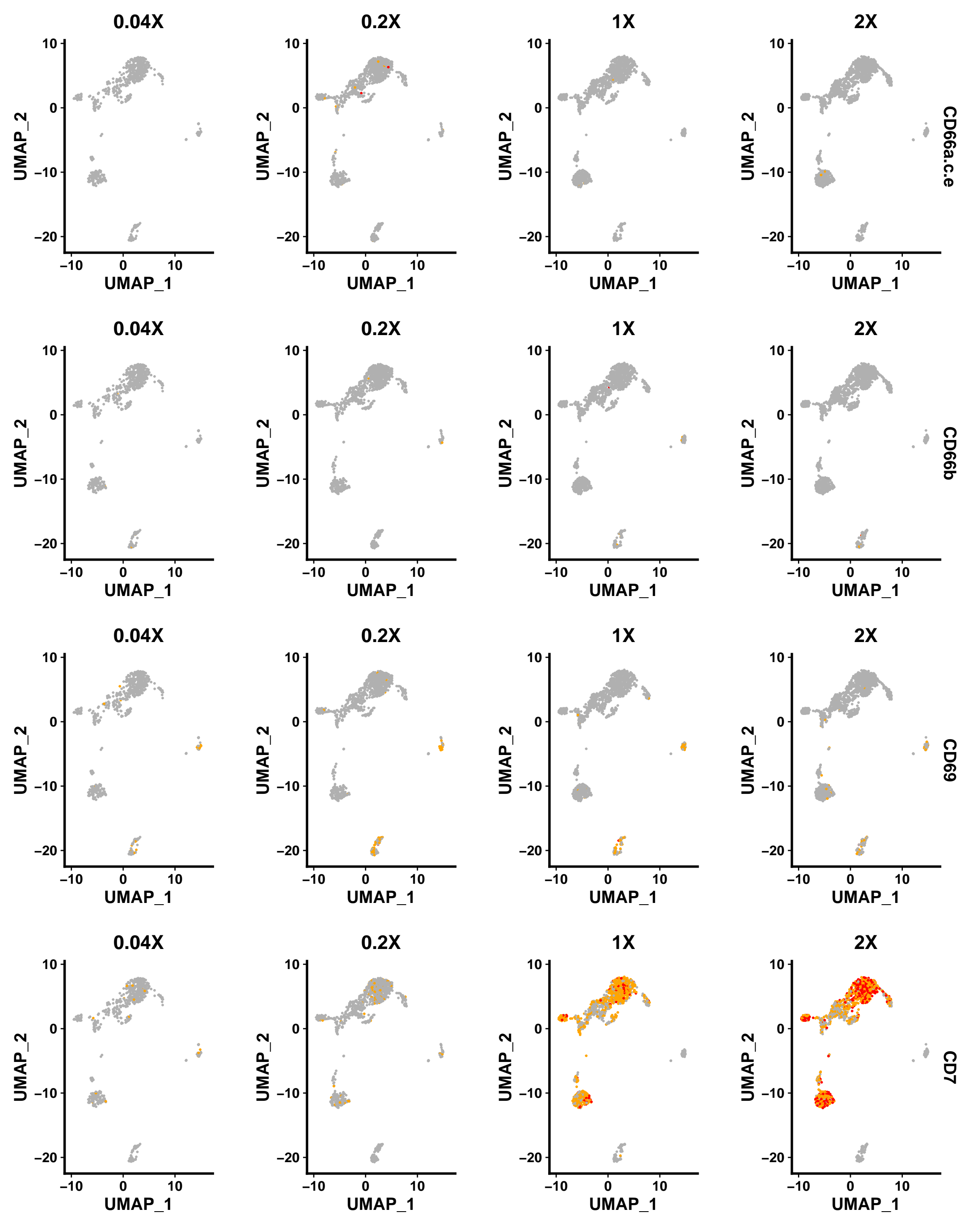

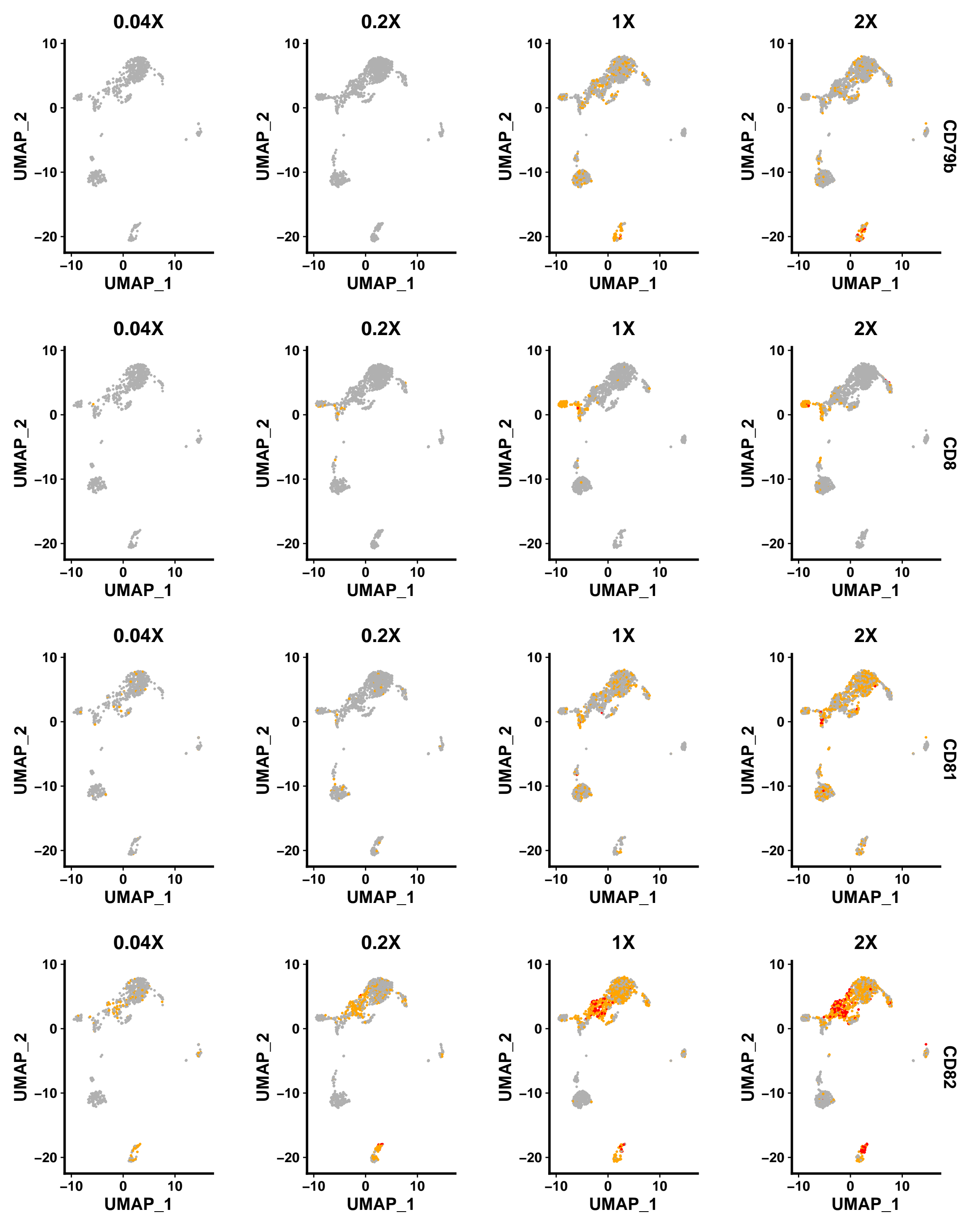

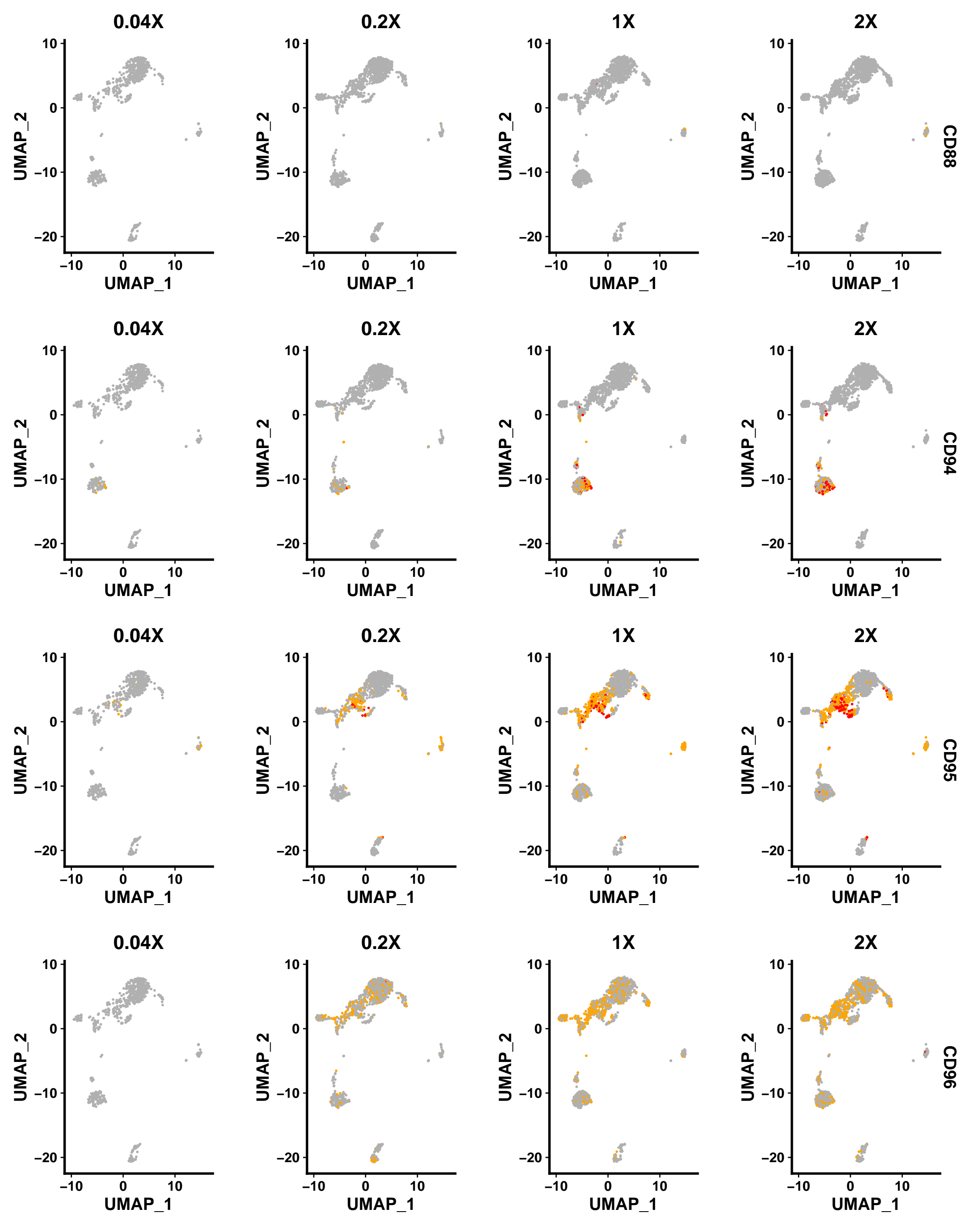

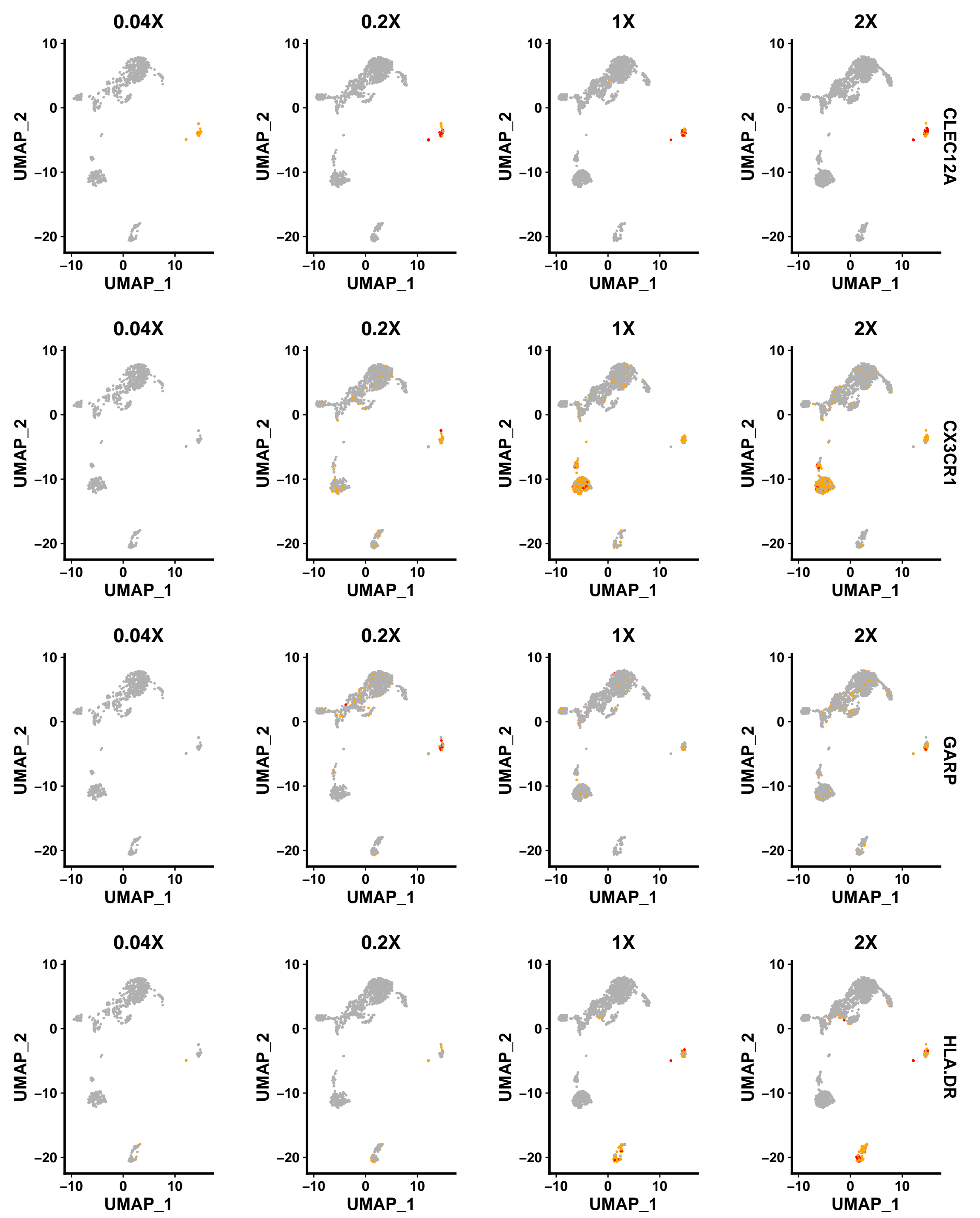

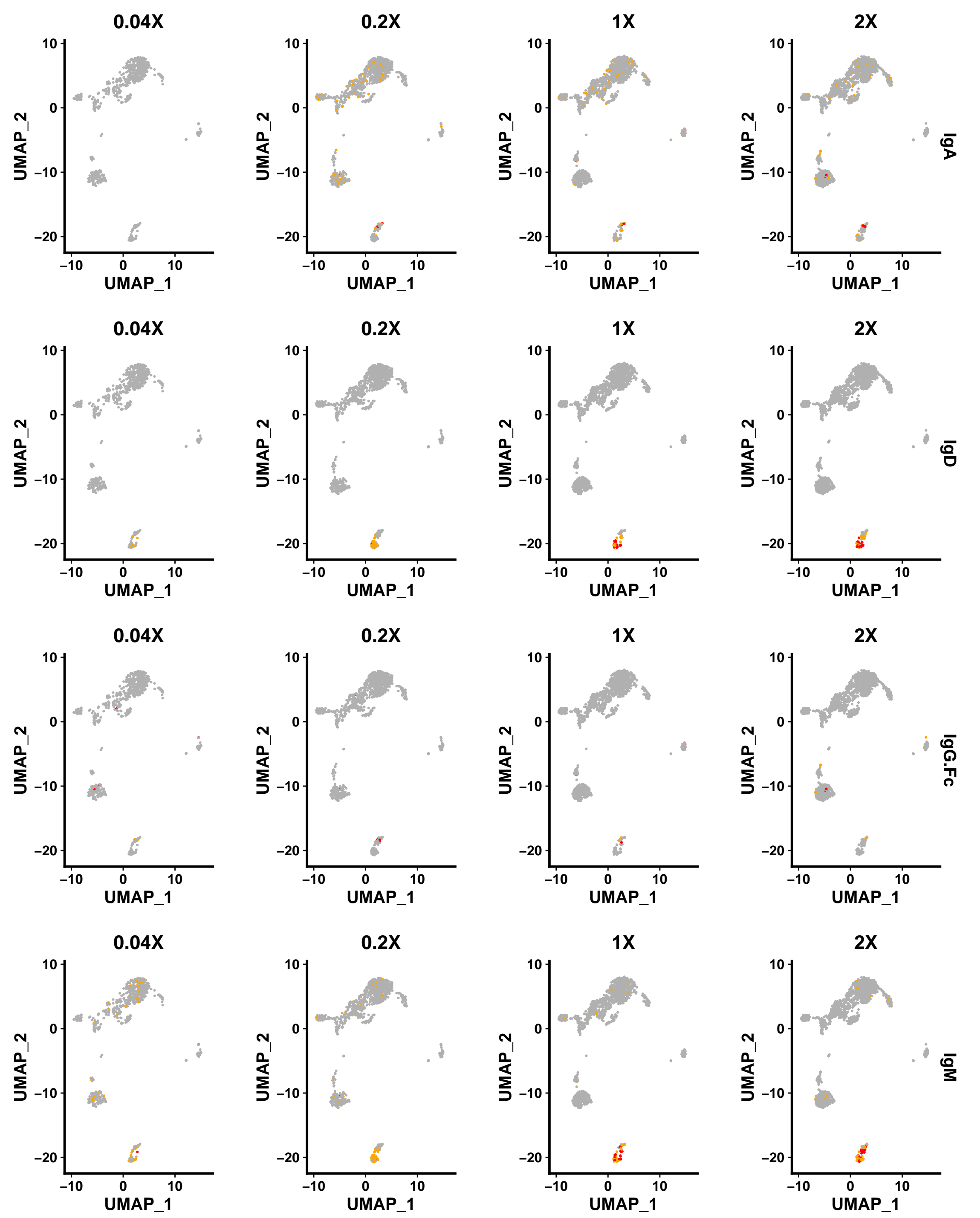

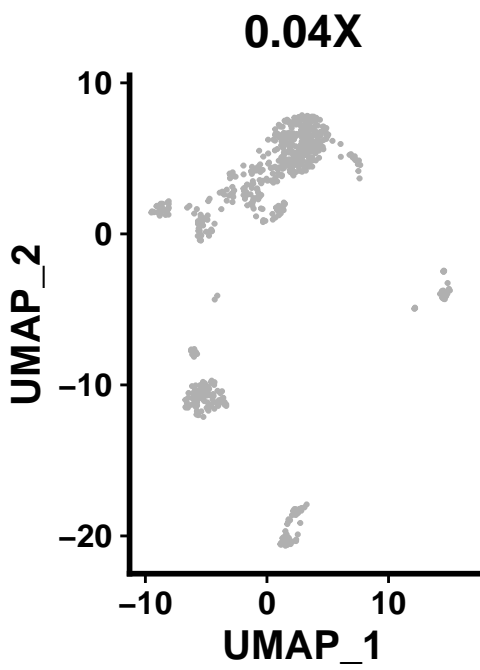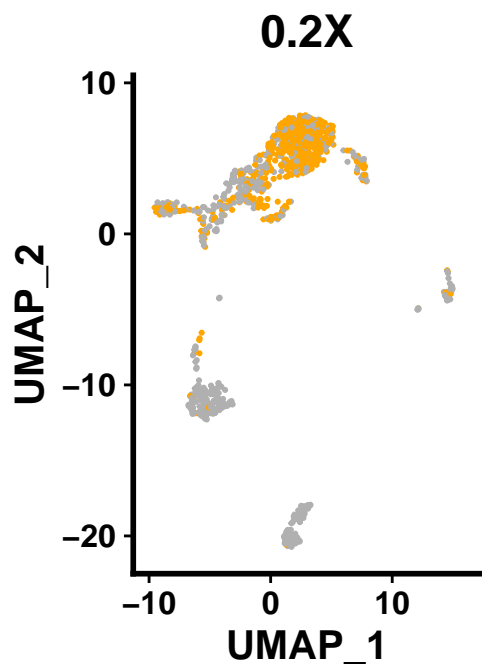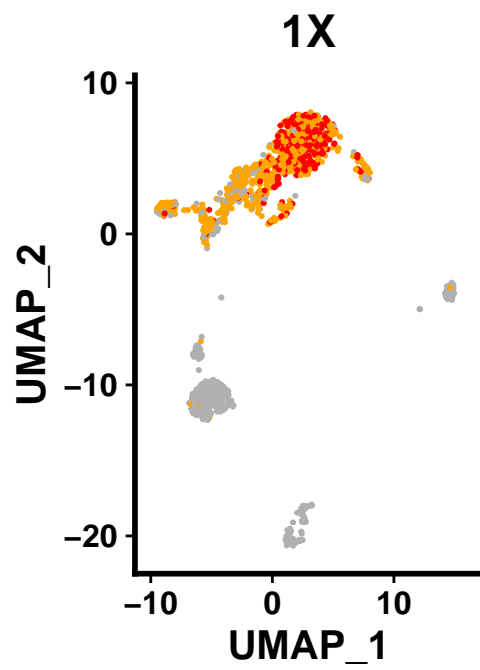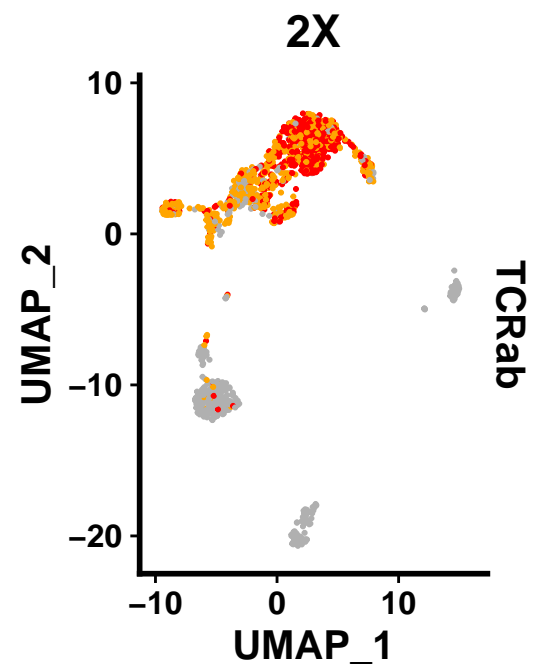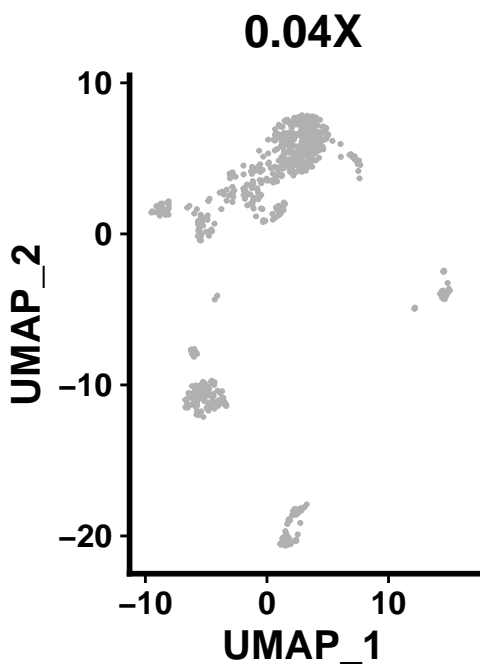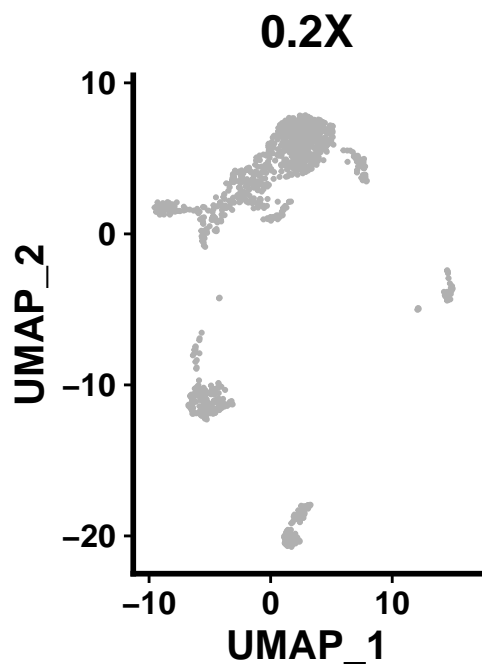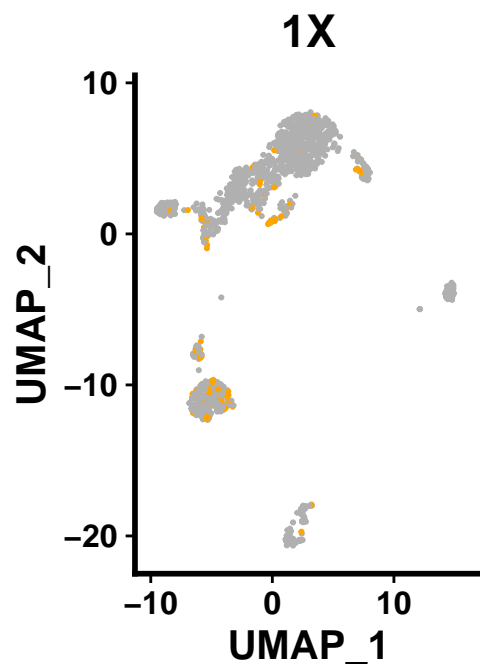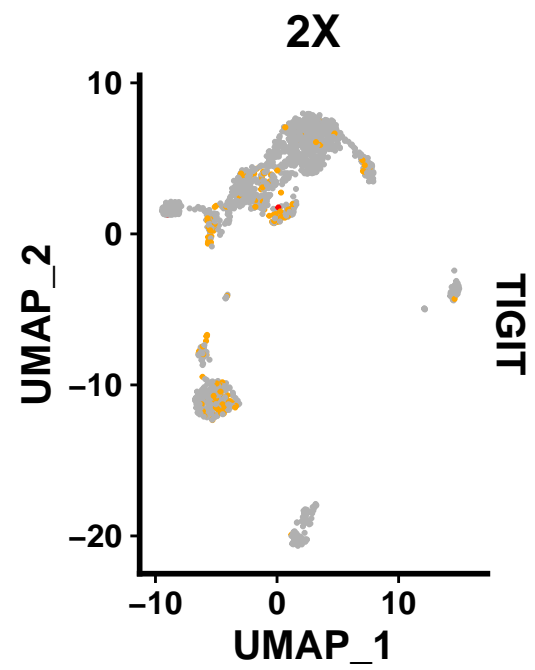

Supplement: Supplementary file 4 — Supplementary Information 4. [file 41598_2022_24371_MOESM4_ESM.pdf]
