# Supplementary material for: Titration of 124 antibodies using CITE-Seq on human PBMCs
Source: Sci Rep. 2022 Dec 2;12:20817. doi: 10.1038/s41598-022-24371-7 (PMC9718773; doi:10.1038/s41598-022-24371-7)
Supplement: Supplementary file 9 — Supplementary Information 9. [file 41598_2022_24371_MOESM9_ESM.pdf]

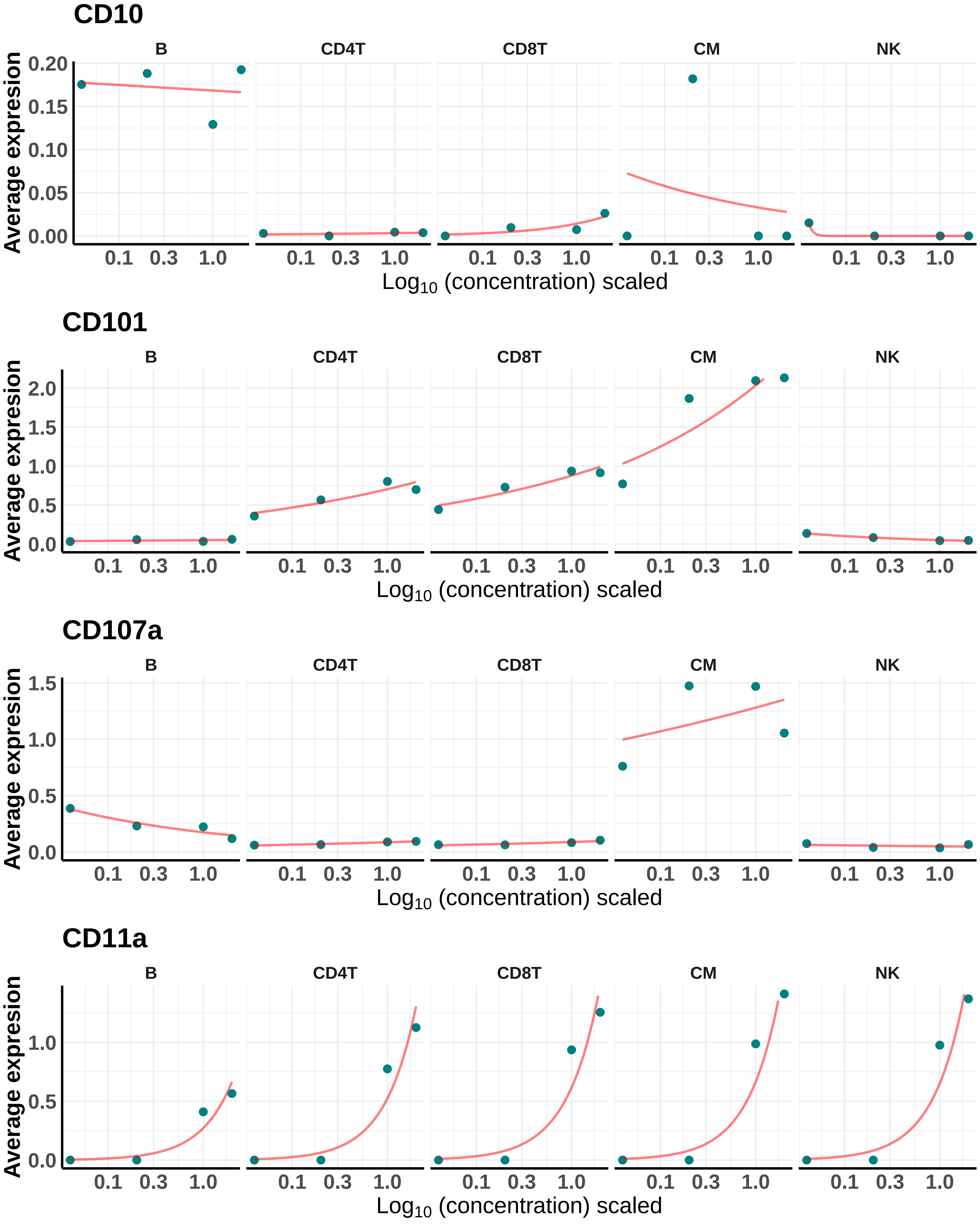
CD10
B
CD4T
CD8T
CM
NK
Average expresion
0.20
0.15
0.10
0.05
0.00
0.1
0.3
1.0
Log10 (concentration) scaled
CD101
2.0
1.5
1.0
0.5
0.0
CD107a
CD11a

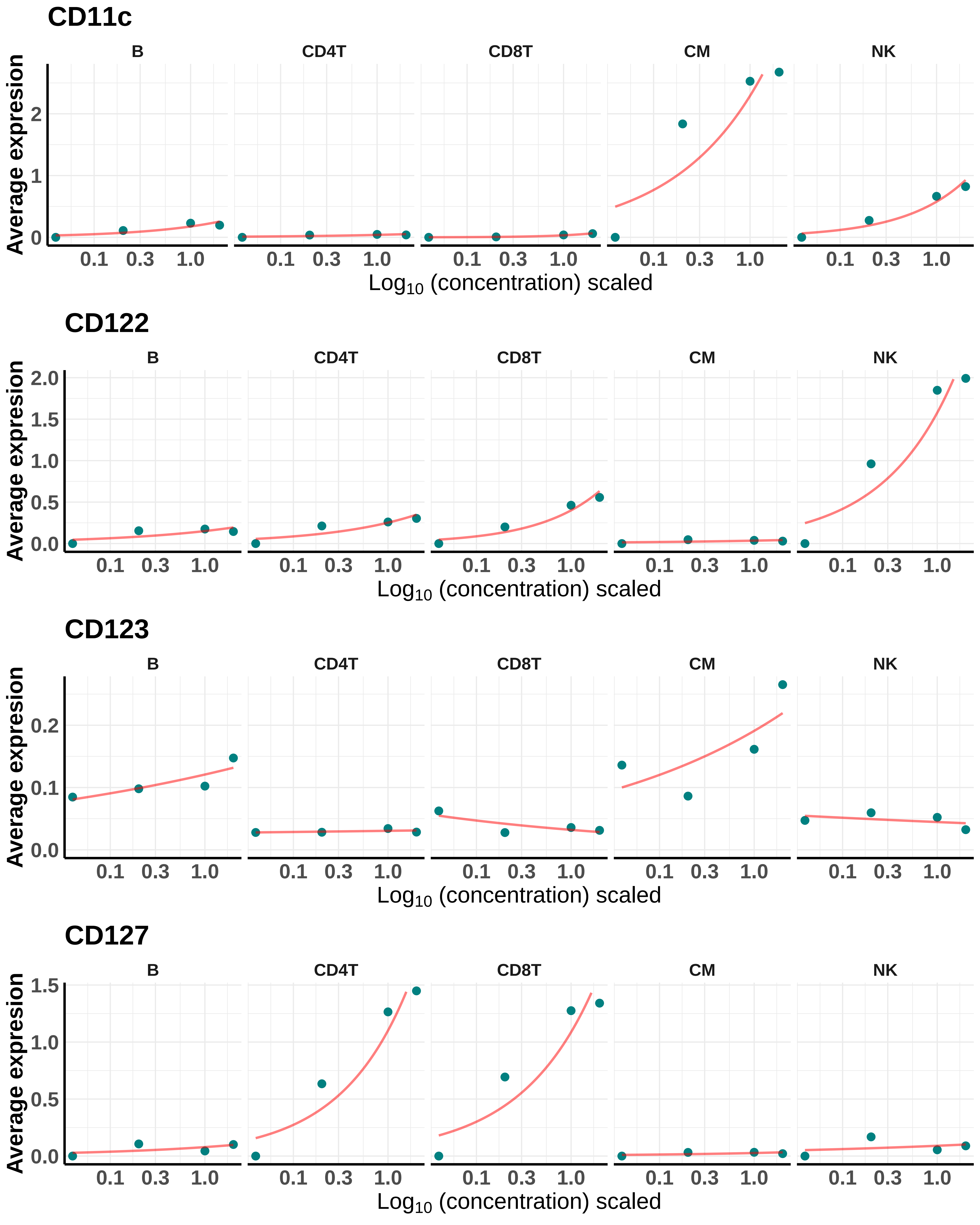

CD11c
CD122
CD123
CD127
B
CD4T
CD8T
CM
NK
Average expresion
Log10 (concentration) scaled
0.1
0.3
1.0

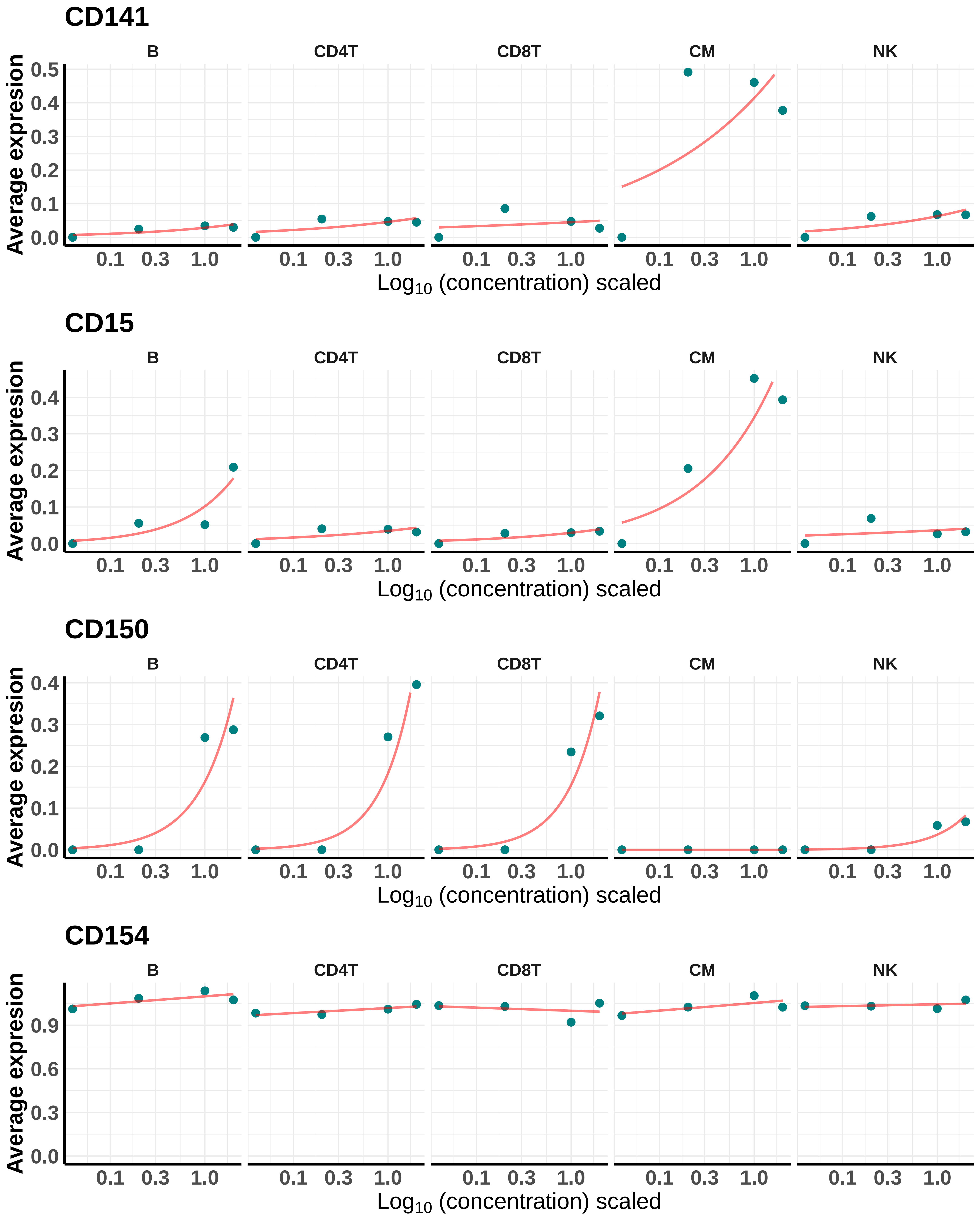
CD141
B
CD4T
CD8T
CM
NK
Average expresion
0.0
0.1
0.2
0.3
0.4
0.5
0.1 0.3 1.0
Log10 (concentration) scaled
CD15
B
CD4T
CD8T
CM
NK
Average expresion
0.0
0.1
0.2
0.3
0.4
0.1 0.3 1.0
Log10 (concentration) scaled
CD150
B
CD4T
CD8T
CM
NK
Average expresion
0.0
0.1
0.2
0.3
0.4
0.1 0.3 1.0
Log10 (concentration) scaled
CD154
B
CD4T
CD8T
CM
NK
Average expresion
0.0
0.3
0.6
0.9
0.1 0.3 1.0
Log10 (concentration) scaled

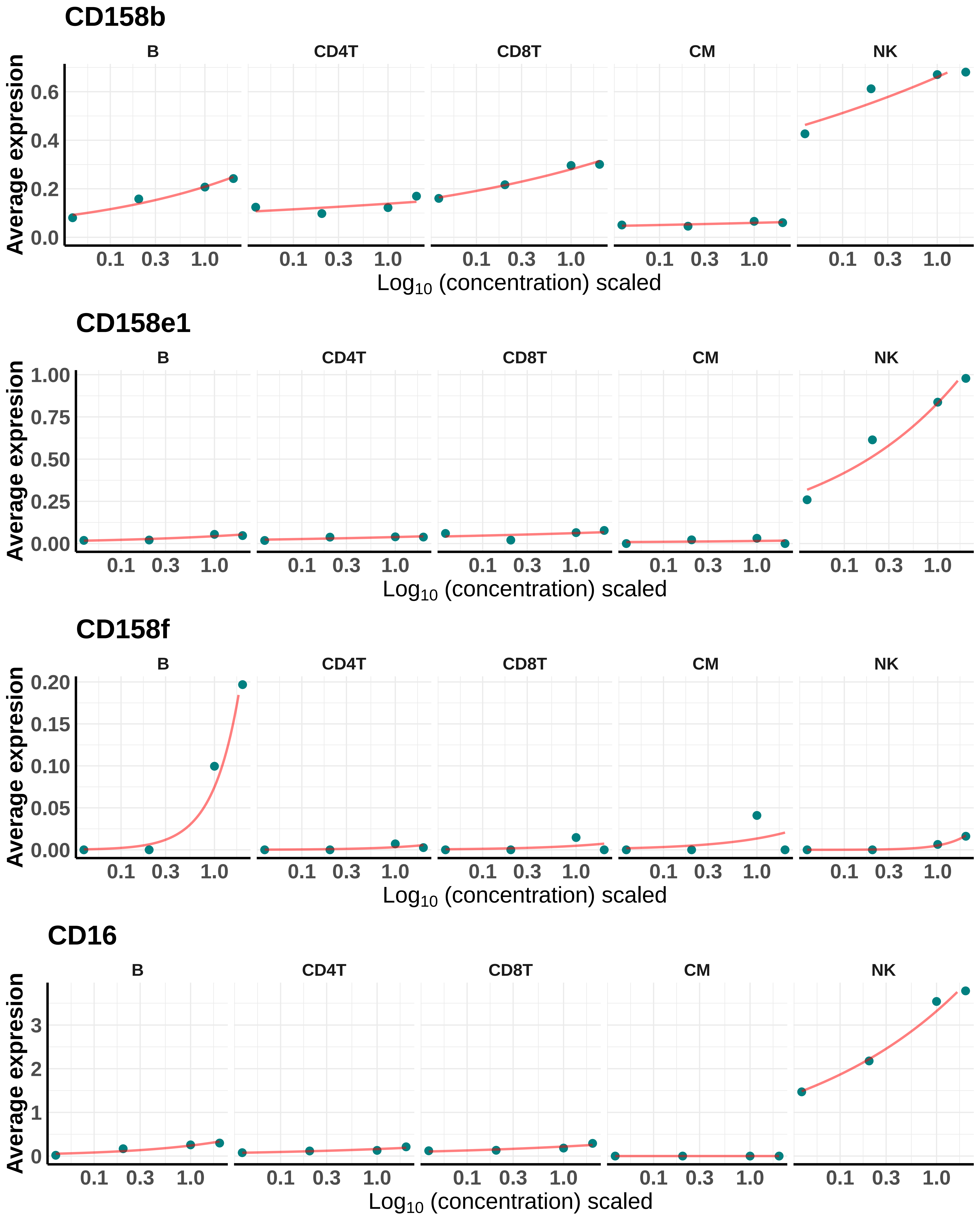
CD158b
B
CD4T
CD8T
CM
NK
Average expresion
0.0
0.2
0.4
0.6
0.1
0.3
1.0
Log10 (concentration) scaled
CD158e1
B
CD4T
CD8T
CM
NK
Average expresion
0.00
0.25
0.50
0.75
1.00
0.1
0.3
1.0
Log10 (concentration) scaled
CD158f
B
CD4T
CD8T
CM
NK
Average expresion
0.00
0.05
0.10
0.15
0.20
0.1
0.3
1.0
Log10 (concentration) scaled
CD16
B
CD4T
CD8T
CM
NK
Average expresion
0
1
2
3
0.1
0.3
1.0
Log10 (concentration) scaled

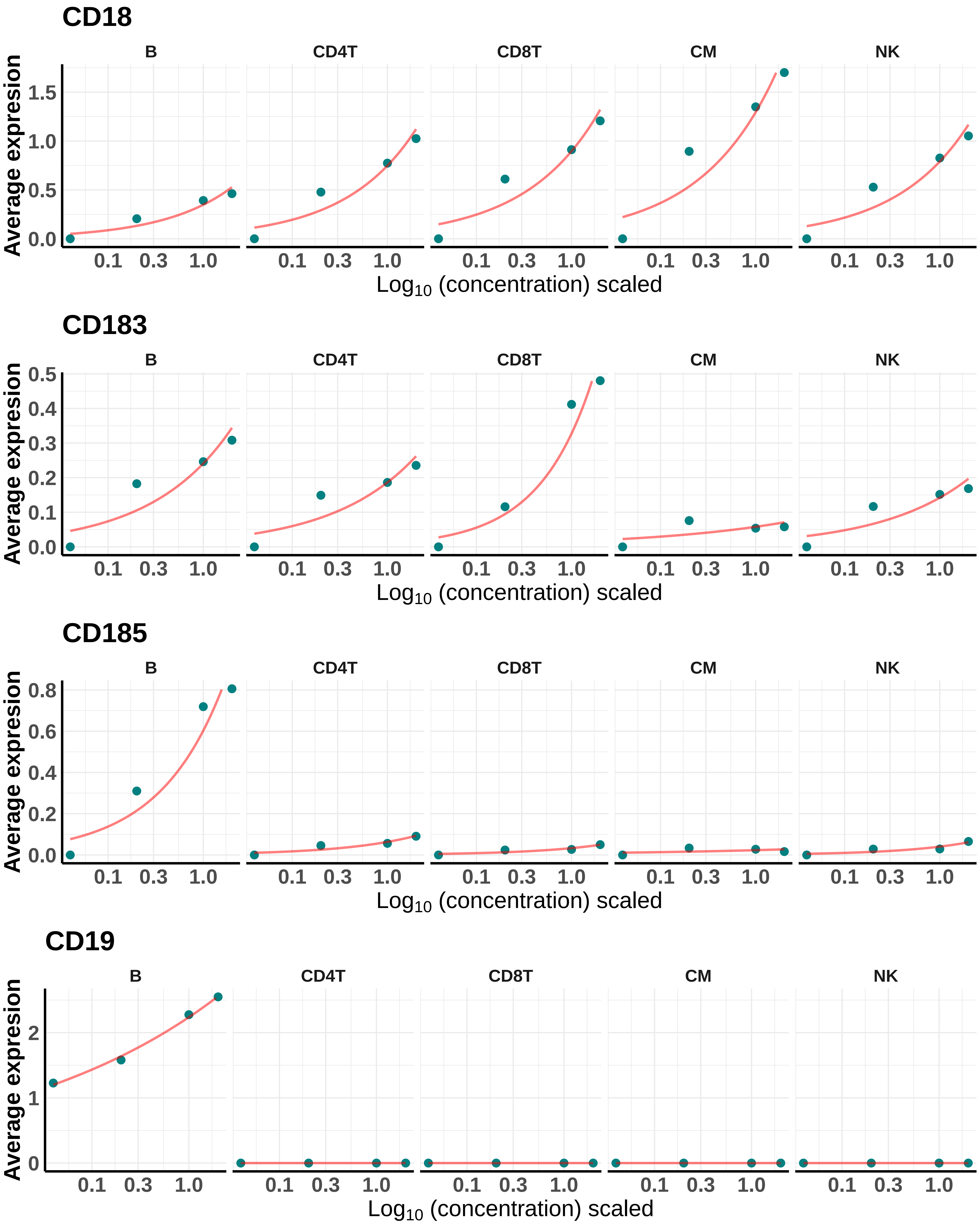

CD18
B
CD4T
CD8T
CM
NK
Average expresion
1.5
1.0
0.5
0.0
0.1
0.3
1.0
Log10 (concentration) scaled
CD183
B
CD4T
CD8T
CM
NK
Average expresion
0.5
0.4
0.3
0.2
0.1
0.0
0.1
0.3
1.0
Log10 (concentration) scaled
CD185
B
CD4T
CD8T
CM
NK
Average expresion
0.8
0.6
0.4
0.2
0.0
0.1
0.3
1.0
Log10 (concentration) scaled
CD19
B
CD4T
CD8T
CM
NK
Average expresion
2
1
0
0.1
0.3
1.0
Log10 (concentration) scaled

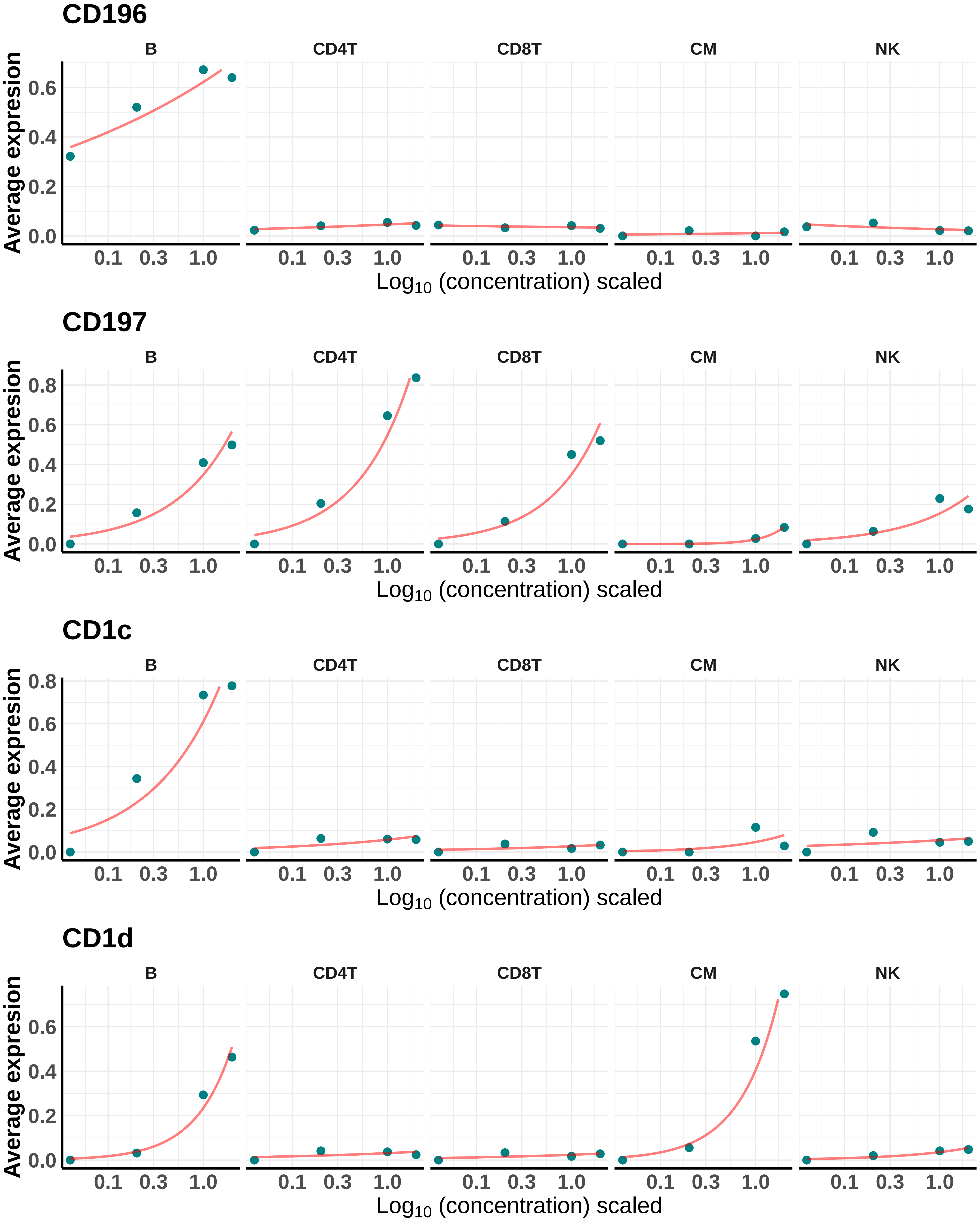
CD196
CD197
CD1c
CD1d
B
CD4T
CD8T
CM
NK
Average expresion
Log10 (concentration) scaled
0.0
0.2
0.4
0.6
0.8
0.1
0.3
1.0

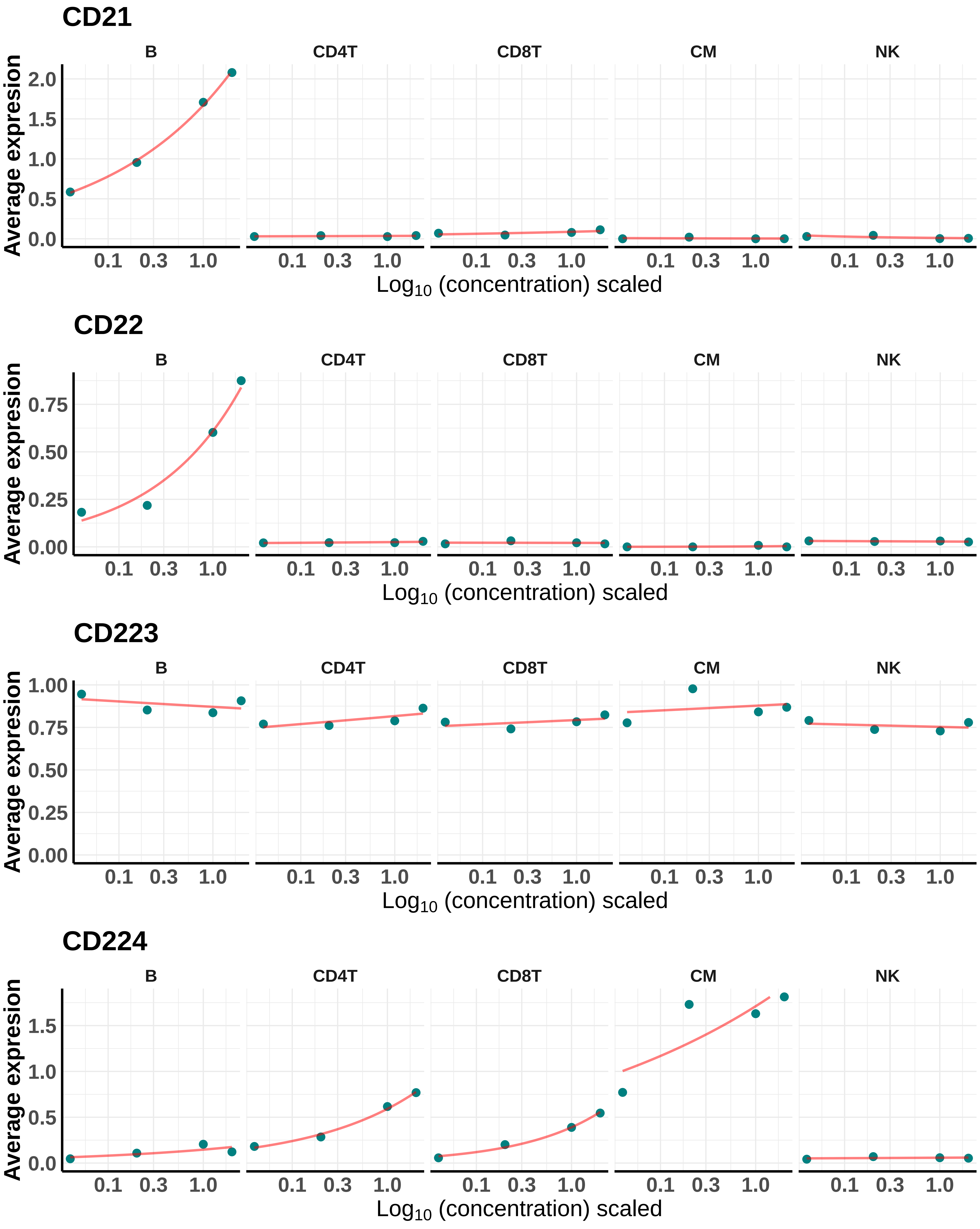
CD21
CD22
CD223
CD224
B
CD4T
CD8T
CM
NK
Average expresion
Log10 (concentration) scaled
0.1
0.3
1.0
0.0
0.5
1.0
1.5
2.0
0.00
0.25
0.50
0.75
1.00

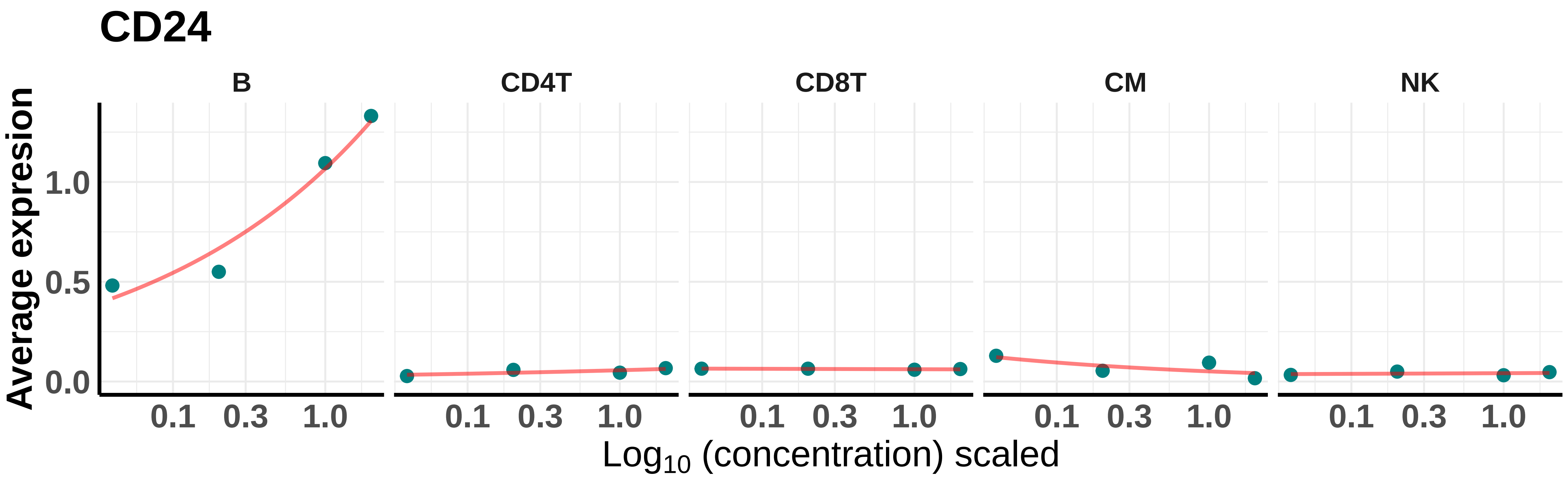
CD24
B
CD4T
CD8T
CM
NK
Average expresion
0.0
0.5
1.0
0.1
0.3
1.0
Log_{10} (concentration) scaled
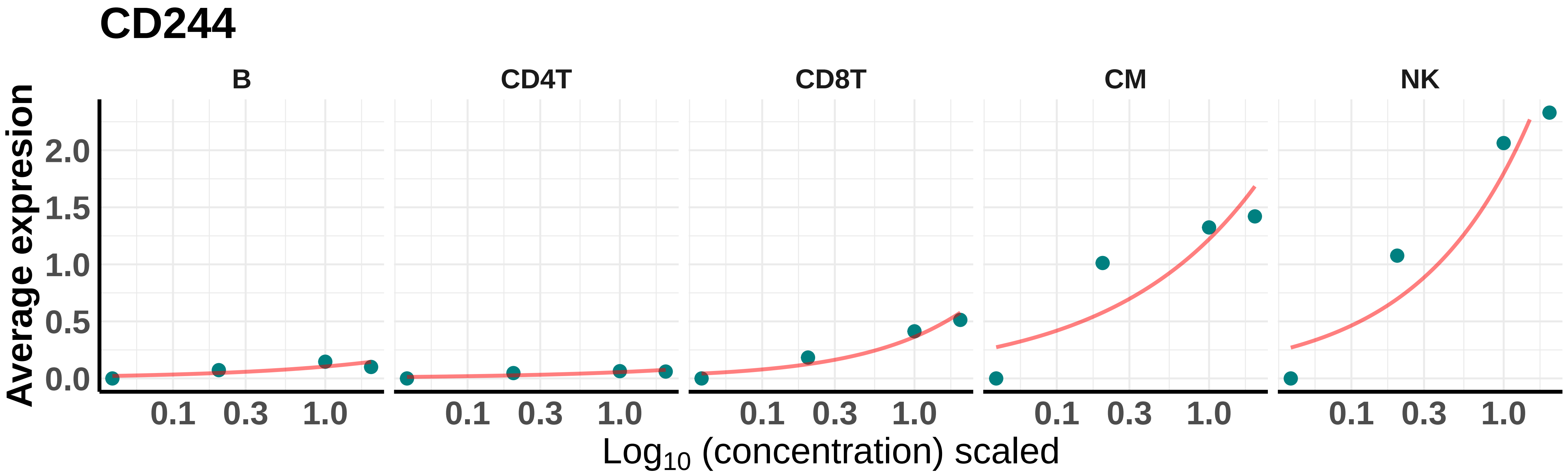
CD244
B
CD4T
CD8T
CM
NK
Average expresion
0.0
0.5
1.0
1.5
2.0
0.1
0.3
1.0
Log_{10} (concentration) scaled
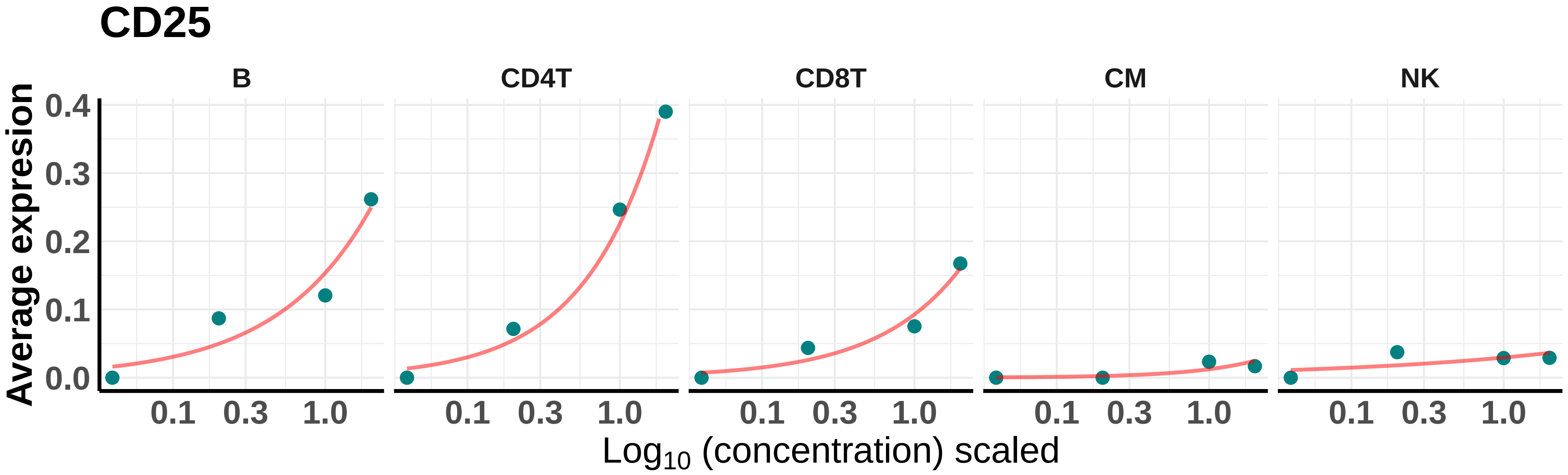
CD25
B
CD4T
CD8T
CM
NK
Average expresion
0.0
0.1
0.2
0.3
0.4
0.1
0.3
1.0
Log_{10} (concentration) scaled
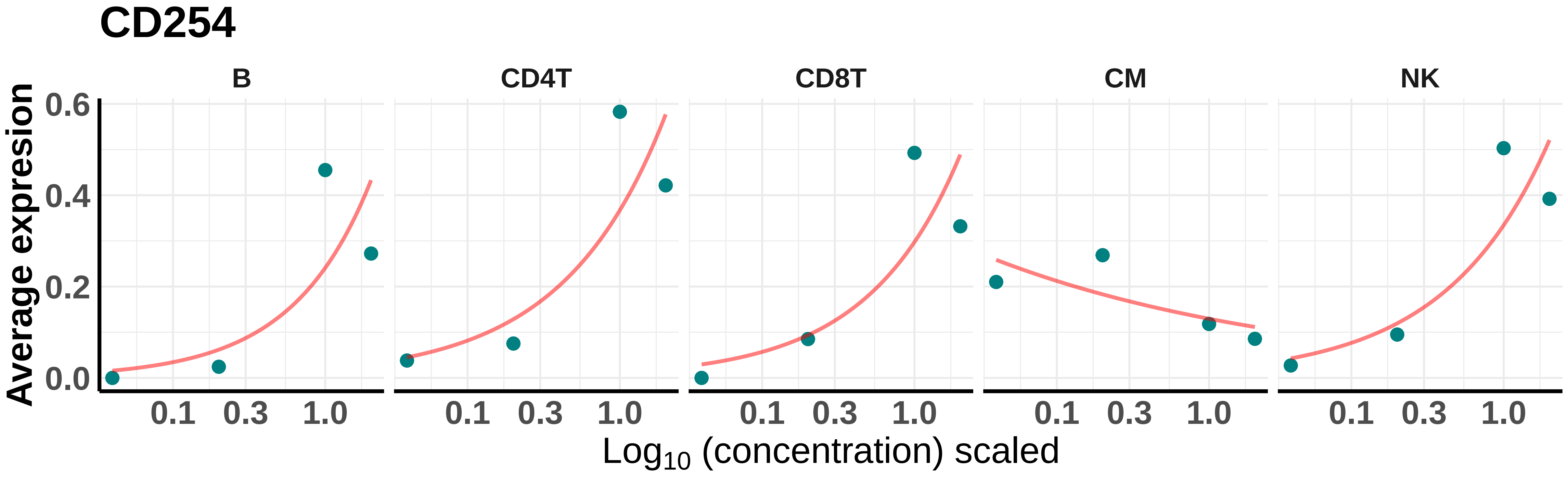
CD254
B
CD4T
CD8T
CM
NK
Average expresion
0.0
0.2
0.4
0.6
0.1
0.3
1.0
Log_{10} (concentration) scaled

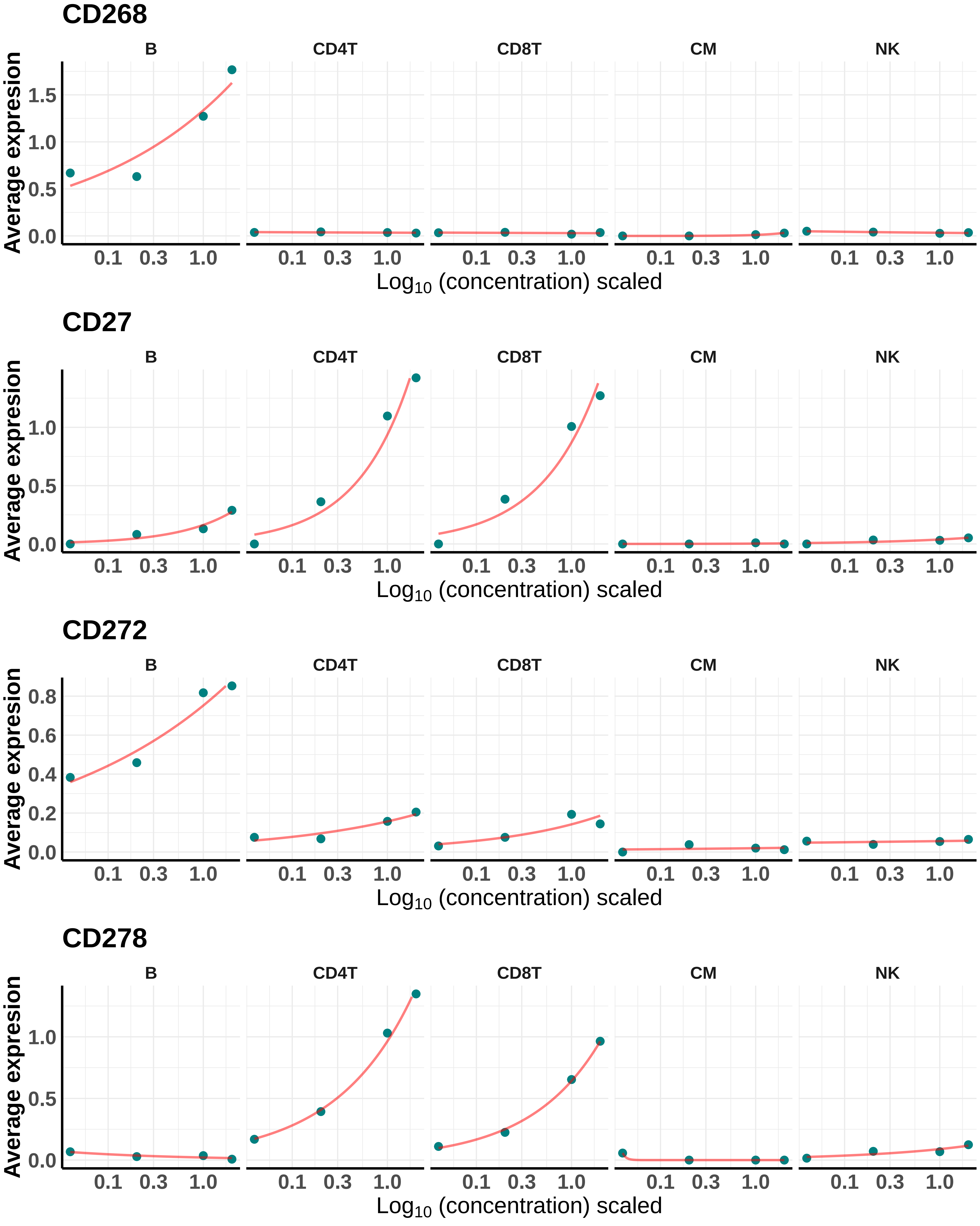

CD268
B
CD4T
CD8T
CM
NK
Average expresion
1.5
1.0
0.5
0.0
0.1
0.3
1.0
Log10 (concentration) scaled
CD27
B
CD4T
CD8T
CM
NK
Average expresion
1.0
0.5
0.0
0.1
0.3
1.0
Log10 (concentration) scaled
CD272
B
CD4T
CD8T
CM
NK
Average expresion
0.8
0.6
0.4
0.2
0.0
0.1
0.3
1.0
Log10 (concentration) scaled
CD278
B
CD4T
CD8T
CM
NK
Average expresion
1.0
0.5
0.0
0.1
0.3
1.0
Log10 (concentration) scaled

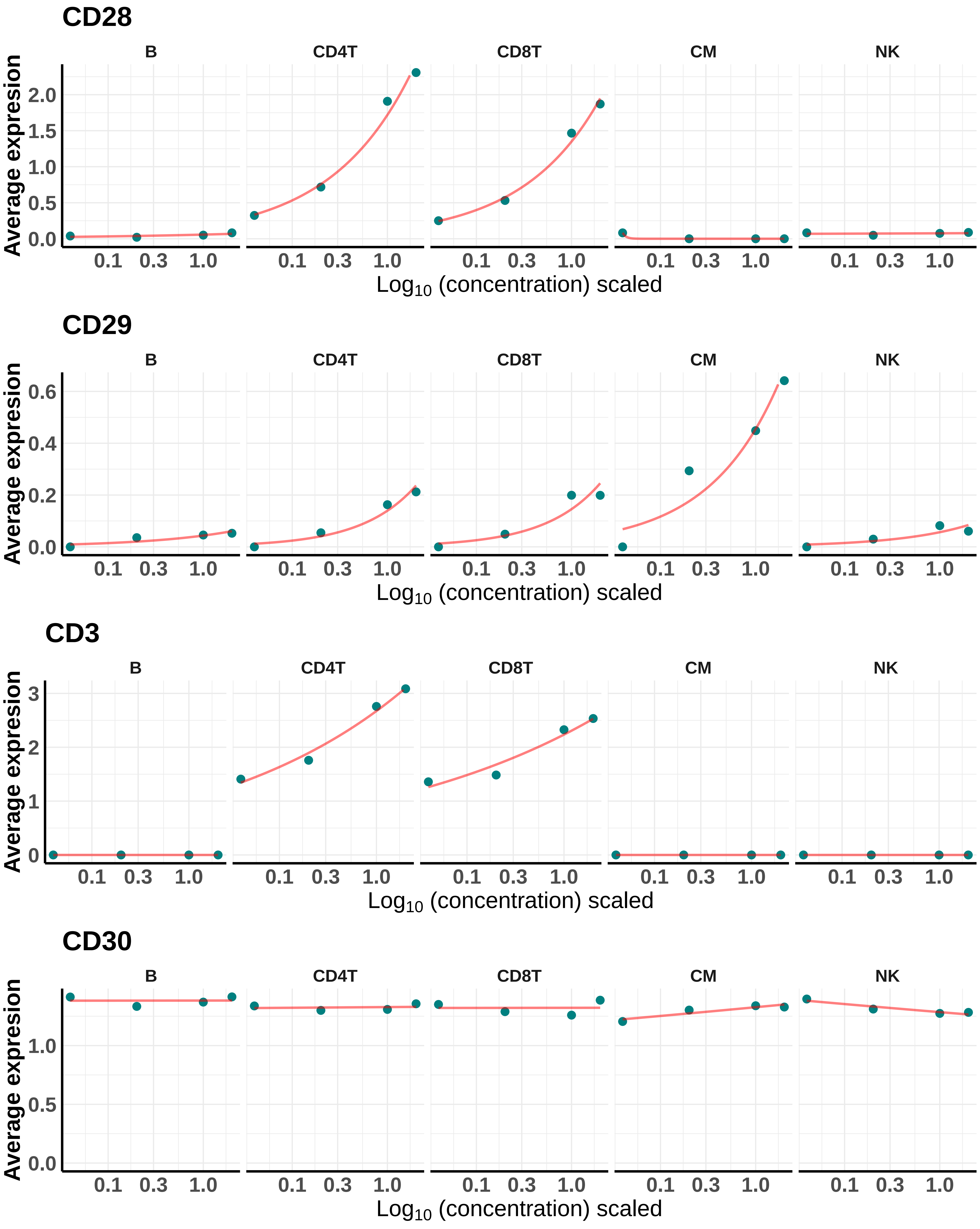
CD28
CD29
CD3
CD30
B
CD4T
CD8T
CM
NK
Average expresion
Log10 (concentration) scaled
0.1
0.3
1.0
0.0
0.5
1.0
1.5
2.0
0.2
0.4
0.6
0
1
2
3

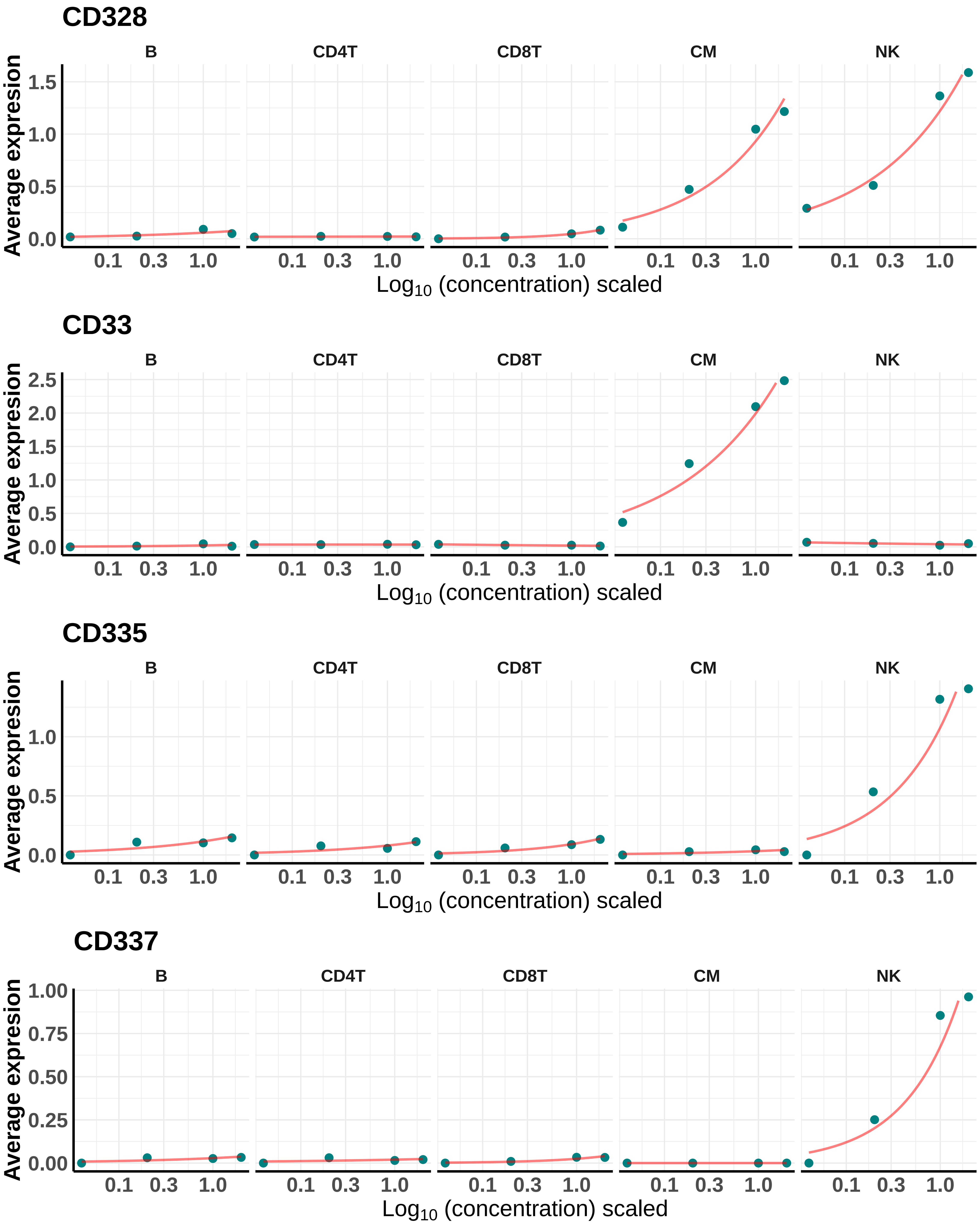
CD328
B
CD4T
CD8T
CM
NK
Average expresion
0.0
0.5
1.0
1.5
0.1
0.3
1.0
Log10 (concentration) scaled
CD33
B
CD4T
CD8T
CM
NK
Average expresion
0.0
0.5
1.0
1.5
2.0
2.5
0.1
0.3
1.0
Log10 (concentration) scaled
CD335
B
CD4T
CD8T
CM
NK
Average expresion
0.0
0.5
1.0
0.1
0.3
1.0
Log10 (concentration) scaled
CD337
B
CD4T
CD8T
CM
NK
Average expresion
0.00
0.25
0.50
0.75
1.00
0.1
0.3
1.0
Log10 (concentration) scaled

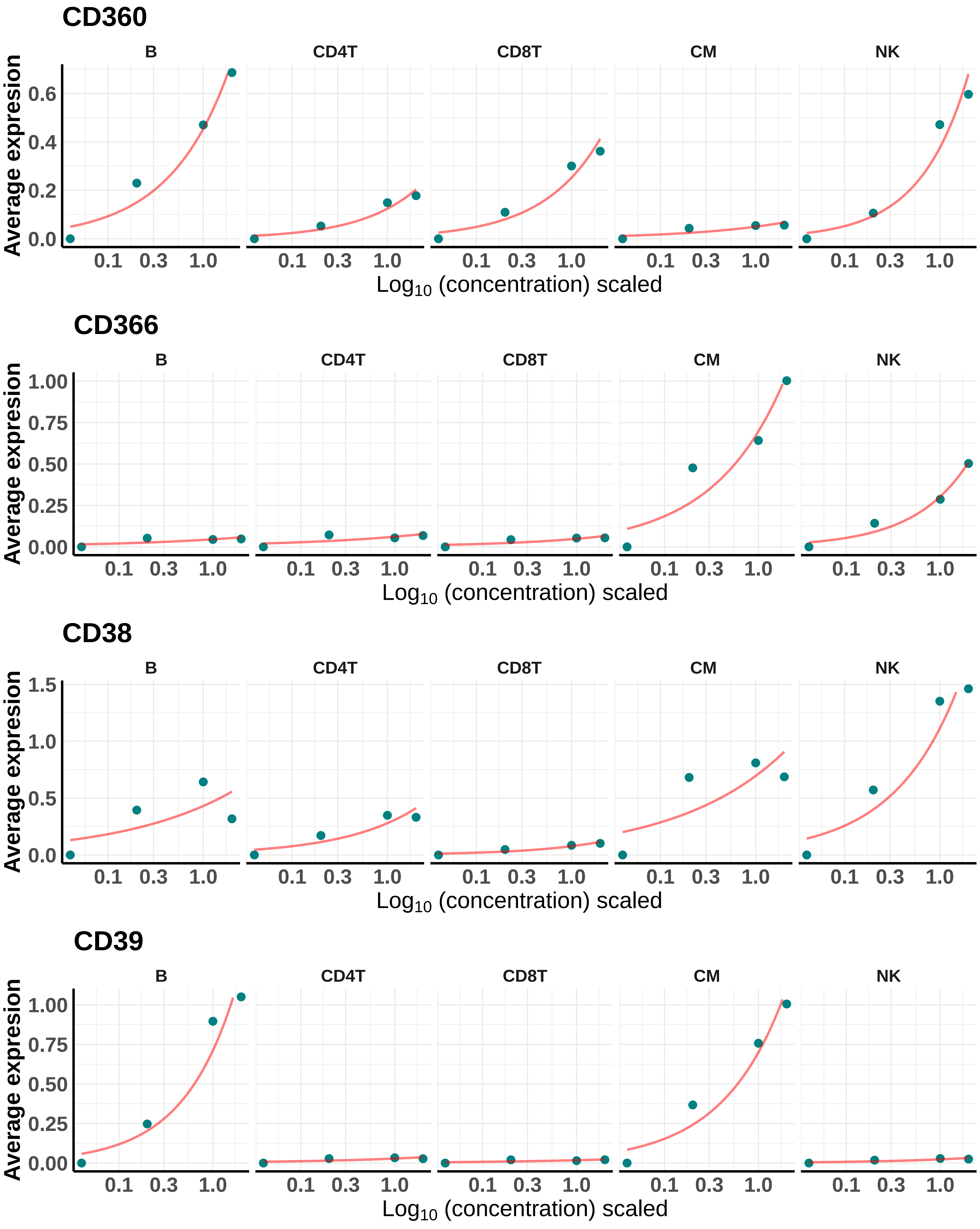
CD360
B
CD4T
CD8T
CM
NK
Average expresion
0.0
0.2
0.4
0.6
0.1
0.3
1.0
Log10 (concentration) scaled
CD366
B
CD4T
CD8T
CM
NK
Average expresion
0.00
0.25
0.50
0.75
1.00
0.1
0.3
1.0
Log10 (concentration) scaled
CD38
B
CD4T
CD8T
CM
NK
Average expresion
0.0
0.5
1.0
1.5
0.1
0.3
1.0
Log10 (concentration) scaled
CD39
B
CD4T
CD8T
CM
NK
Average expresion
0.00
0.25
0.50
0.75
1.00
0.1
0.3
1.0
Log10 (concentration) scaled

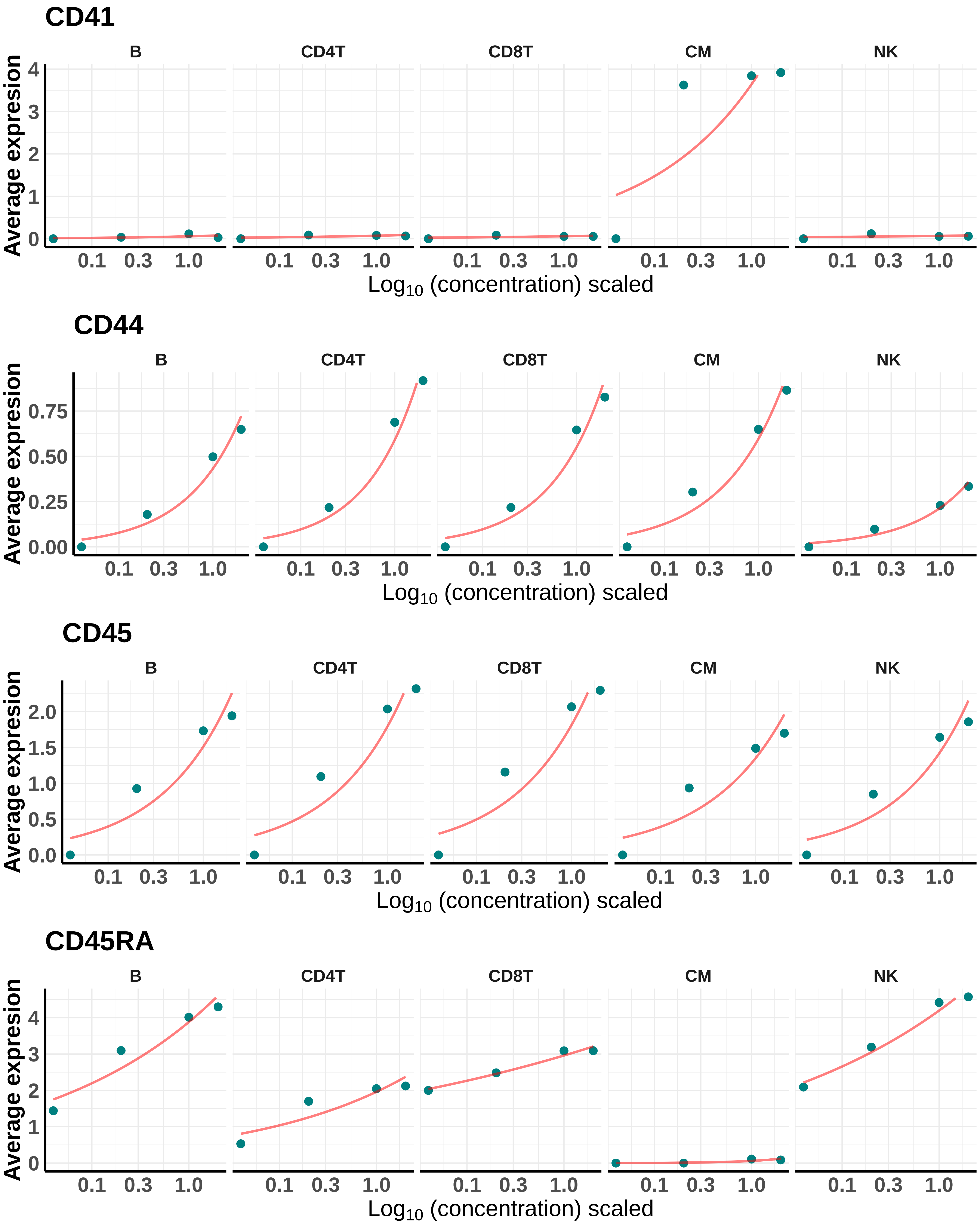
CD41
CD44
CD45
CD45RA
B
CD4T
CD8T
CM
NK
Average expresion
Log10 (concentration) scaled
0.1
0.3
1.0

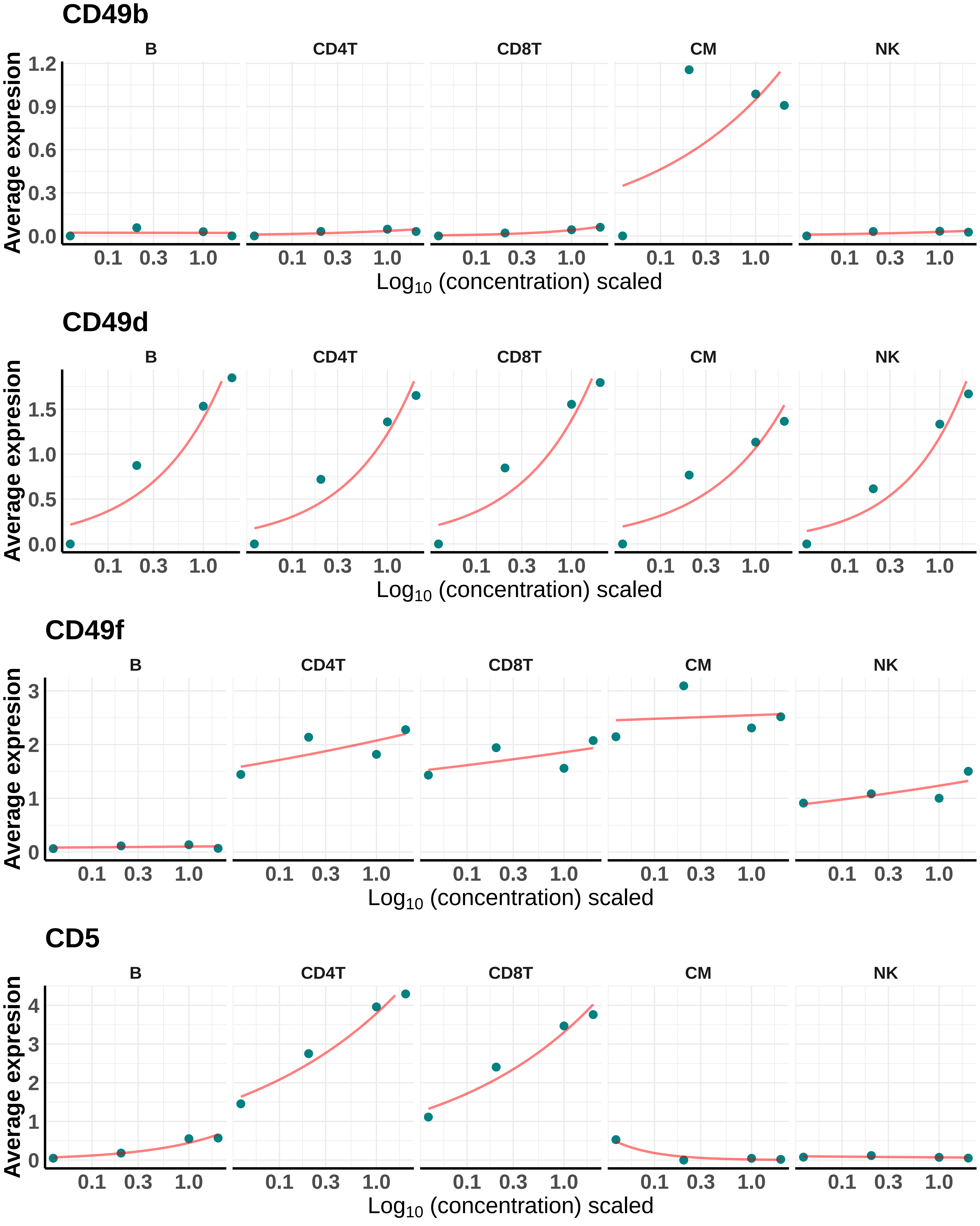
CD49b
CD49d
CD49f
CD5
B
CD4T
CD8T
CM
NK
Average expresion
Log10 (concentration) scaled
0.1
0.3
1.0

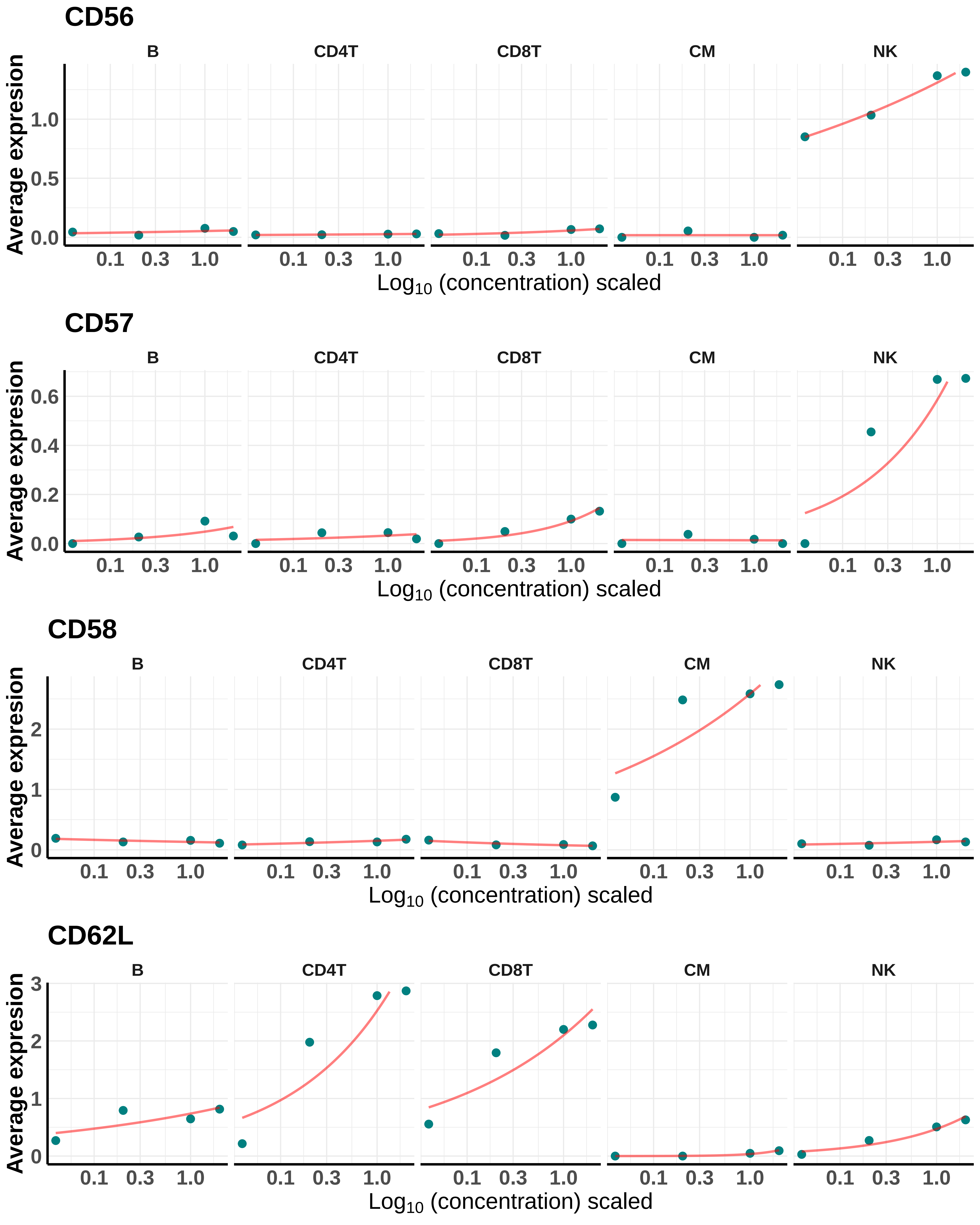
CD56
CD57
CD58
CD62L
B
CD4T
CD8T
CM
NK
Average expresion
Log10 (concentration) scaled
0.1
0.3
1.0

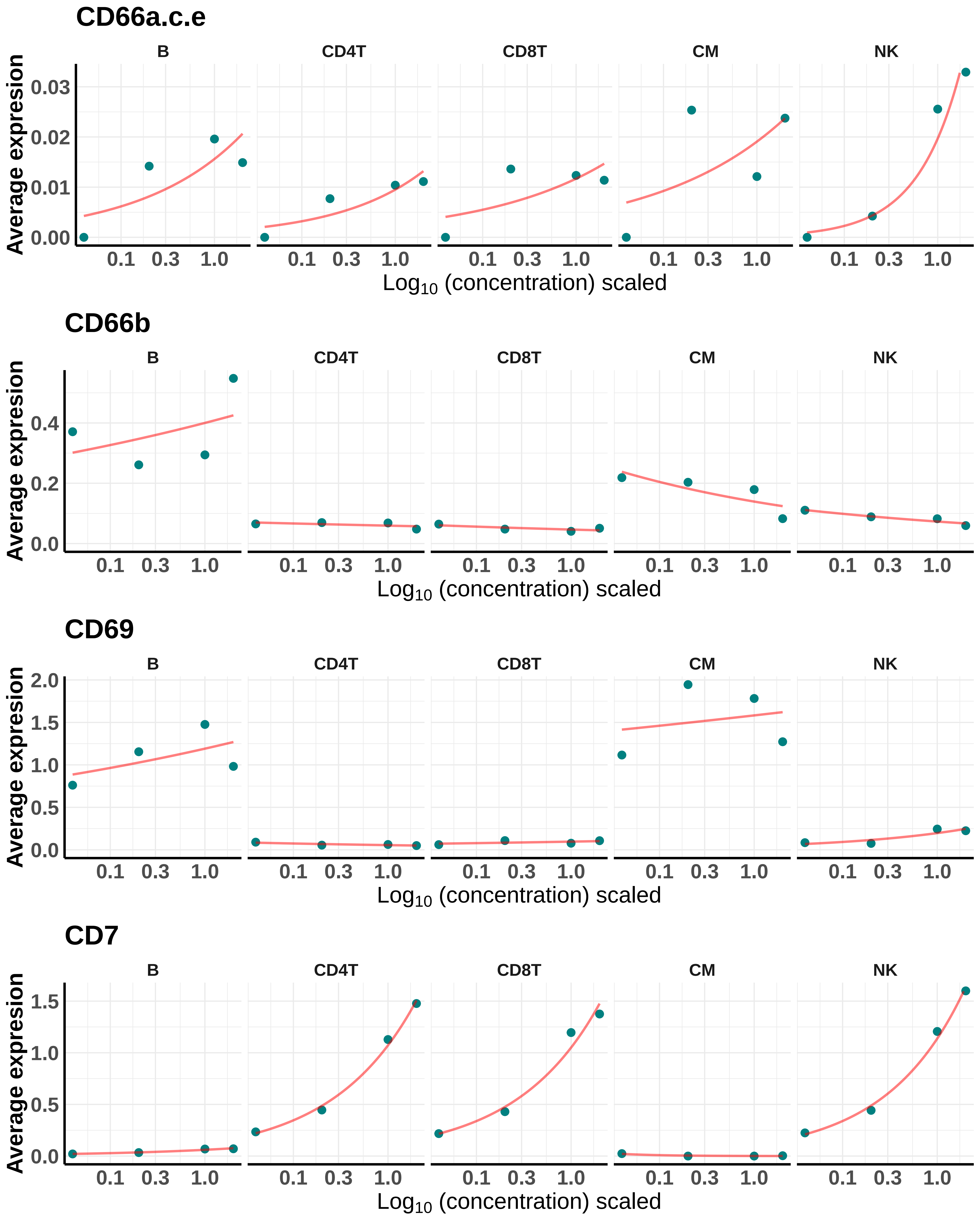
CD66a.c.e
B
CD4T
CD8T
CM
NK
Average expresion
0.00
0.01
0.02
0.03
0.1
0.3
1.0
Log10 (concentration) scaled
CD66b
B
CD4T
CD8T
CM
NK
Average expresion
0.0
0.2
0.4
0.1
0.3
1.0
Log10 (concentration) scaled
CD69
B
CD4T
CD8T
CM
NK
Average expresion
0.0
0.5
1.0
1.5
2.0
0.1
0.3
1.0
Log10 (concentration) scaled
CD7
B
CD4T
CD8T
CM
NK
Average expresion
0.0
0.5
1.0
1.5
0.1
0.3
1.0
Log10 (concentration) scaled

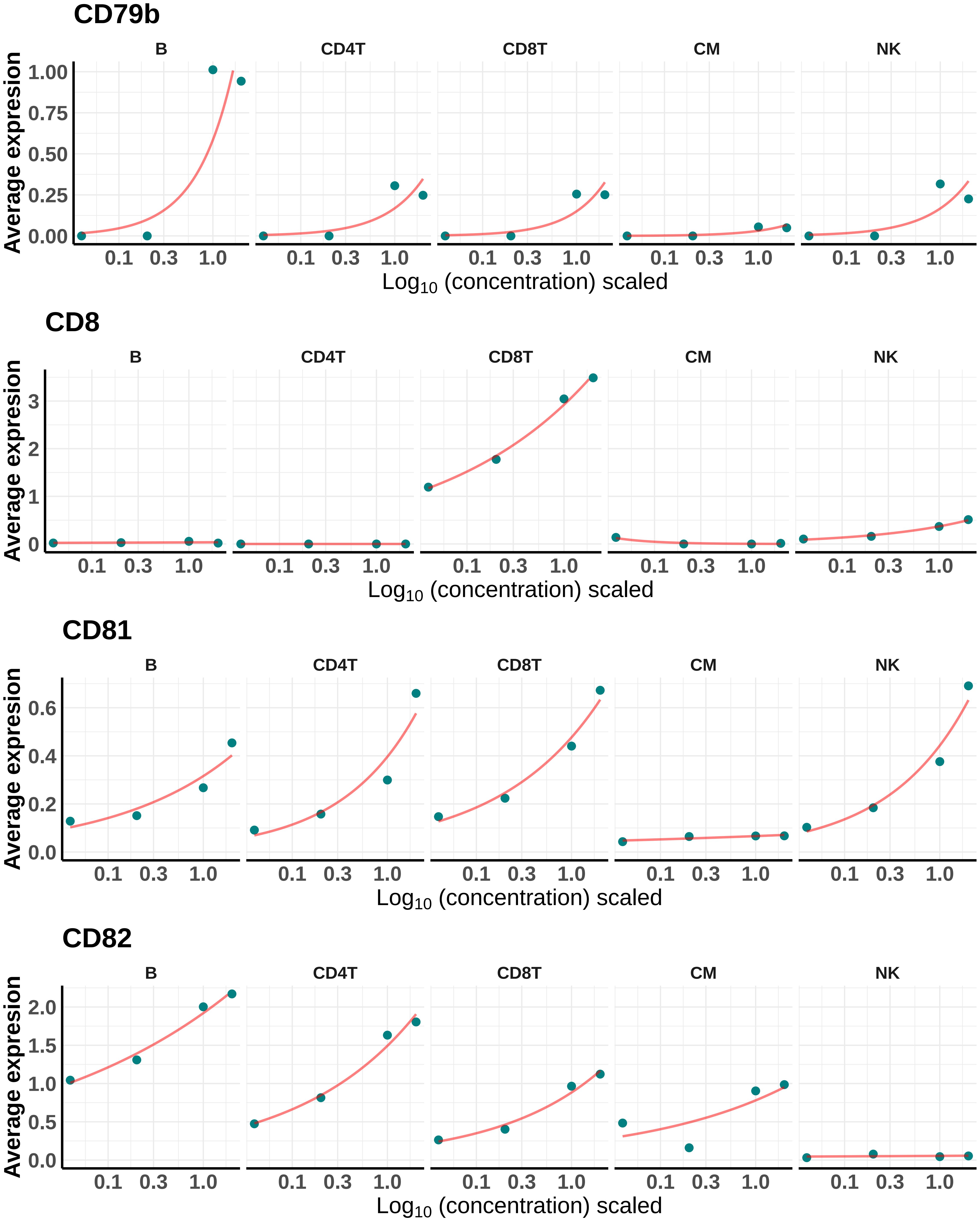
CD79b
B
CD4T
CD8T
CM
NK
Average expresion
1.00
0.75
0.50
0.25
0.00
0.1
0.3
1.0
Log10 (concentration) scaled
CD8
B
CD4T
CD8T
CM
NK
Average expresion
3
2
1
0
0.1
0.3
1.0
Log10 (concentration) scaled
CD81
B
CD4T
CD8T
CM
NK
Average expresion
0.6
0.4
0.2
0.0
0.1
0.3
1.0
Log10 (concentration) scaled
CD82
B
CD4T
CD8T
CM
NK
Average expresion
2.0
1.5
1.0
0.5
0.0
0.1
0.3
1.0
Log10 (concentration) scaled

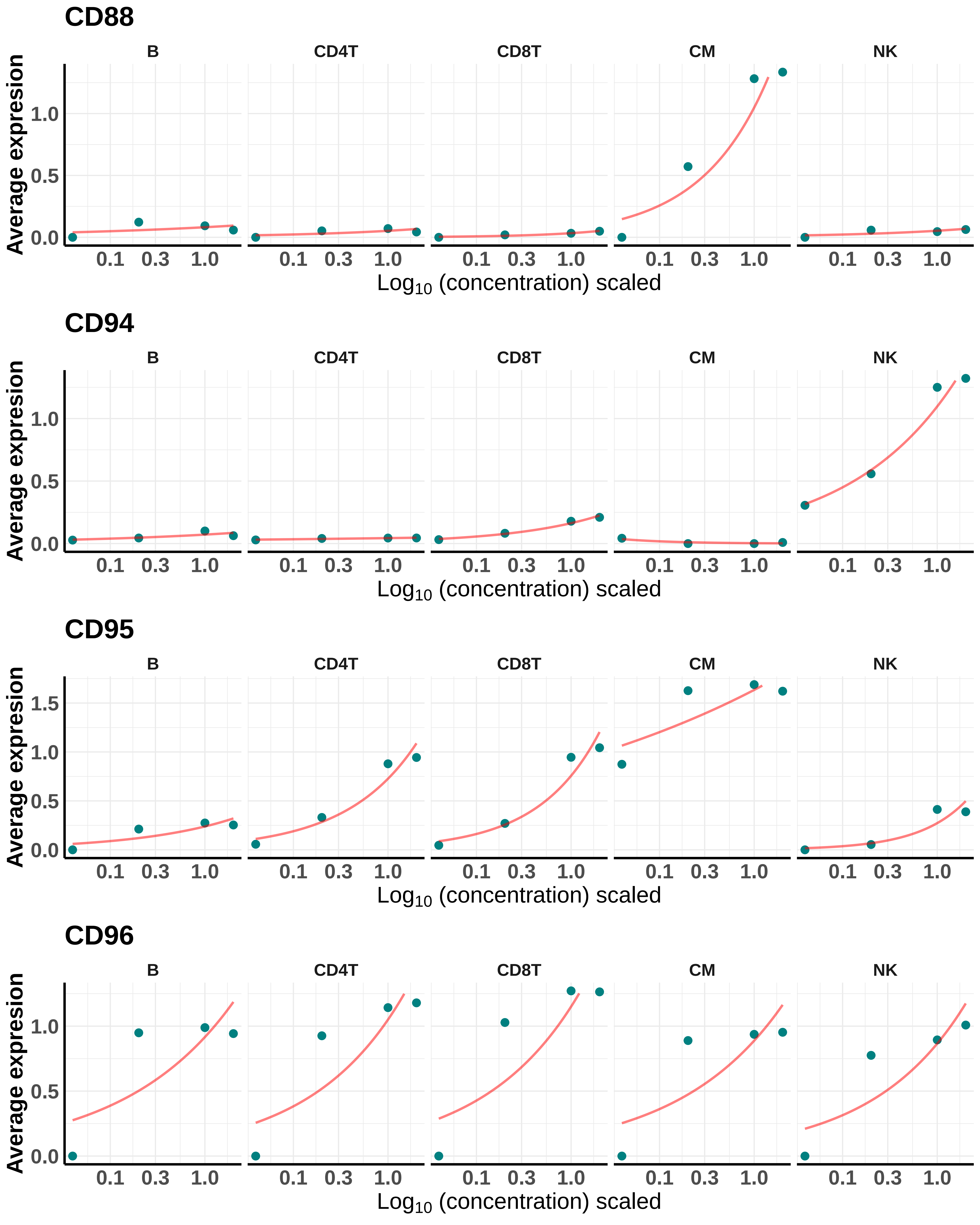
CD88
B
CD4T
CD8T
CM
NK
Average expresion
0.0
0.5
1.0
0.1
0.3
1.0
Log10 (concentration) scaled
CD94
B
CD4T
CD8T
CM
NK
Average expresion
0.0
0.5
1.0
0.1
0.3
1.0
Log10 (concentration) scaled
CD95
B
CD4T
CD8T
CM
NK
Average expresion
0.0
0.5
1.0
1.5
0.1
0.3
1.0
Log10 (concentration) scaled
CD96
B
CD4T
CD8T
CM
NK
Average expresion
0.0
0.5
1.0
0.1
0.3
1.0
Log10 (concentration) scaled

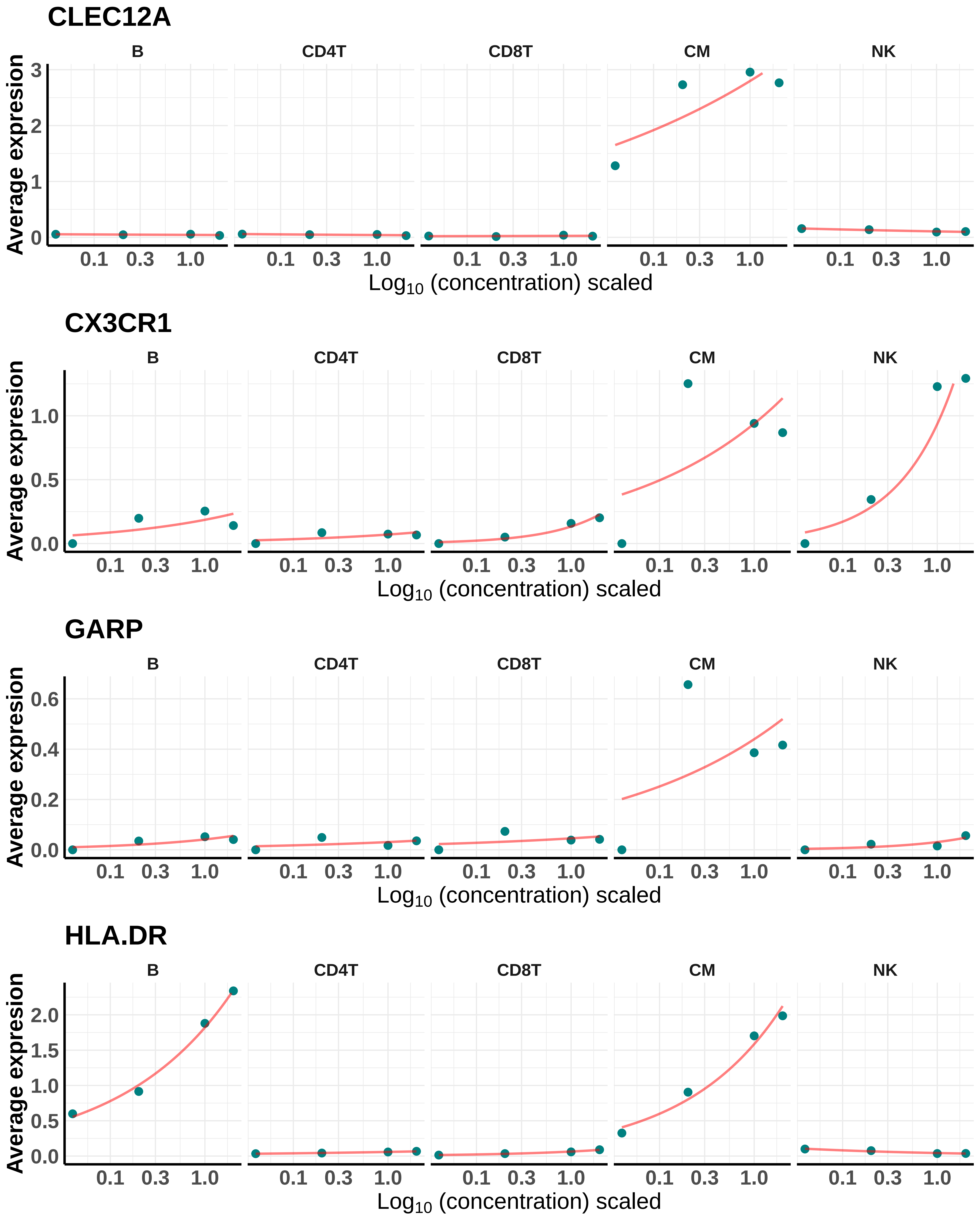
CLEC12A
B
CD4T
CD8T
CM
NK
Average expresion
0
1
2
3
0.1
0.3
1.0
Log10 (concentration) scaled
CX3CR1
0.0
0.5
1.0
GARP
0.2
0.4
0.6
HLA.DR
1.5
2.0

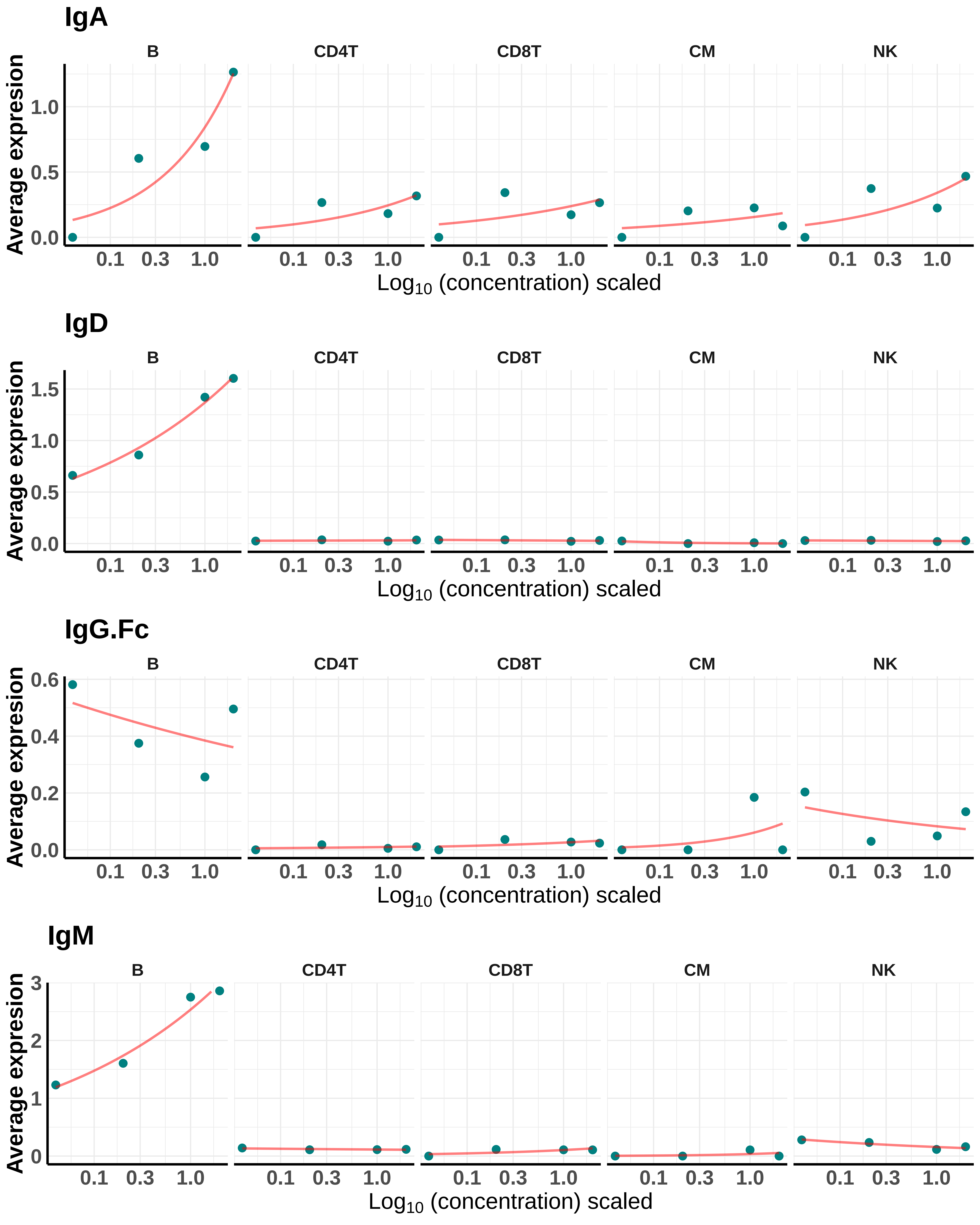
IgA
IgD
IgG.Fc
IgM
B
CD4T
CD8T
CM
NK
Average expresion
Log10 (concentration) scaled
0.1
0.3
1.0
0.0
0.5
1.0
1.5
0.2
0.4
0.6
0
1
2
3

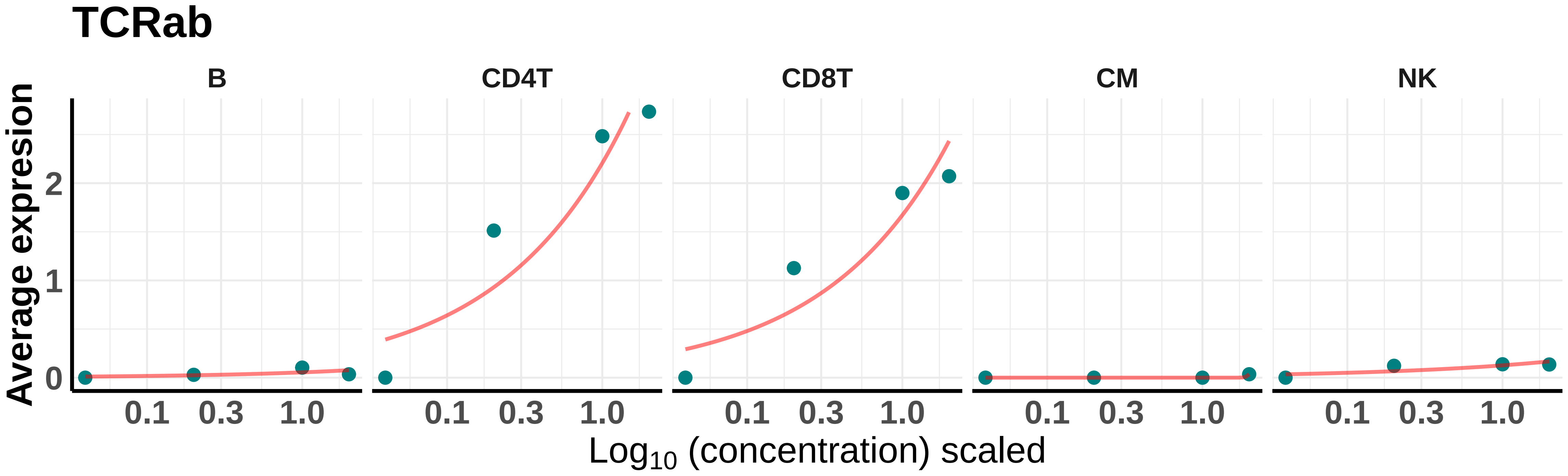
TCRab
B
CD4T
CD8T
CM
NK
Average expresion
0
1
2
0.1
0.3
1.0
Log10 (concentration) scaled

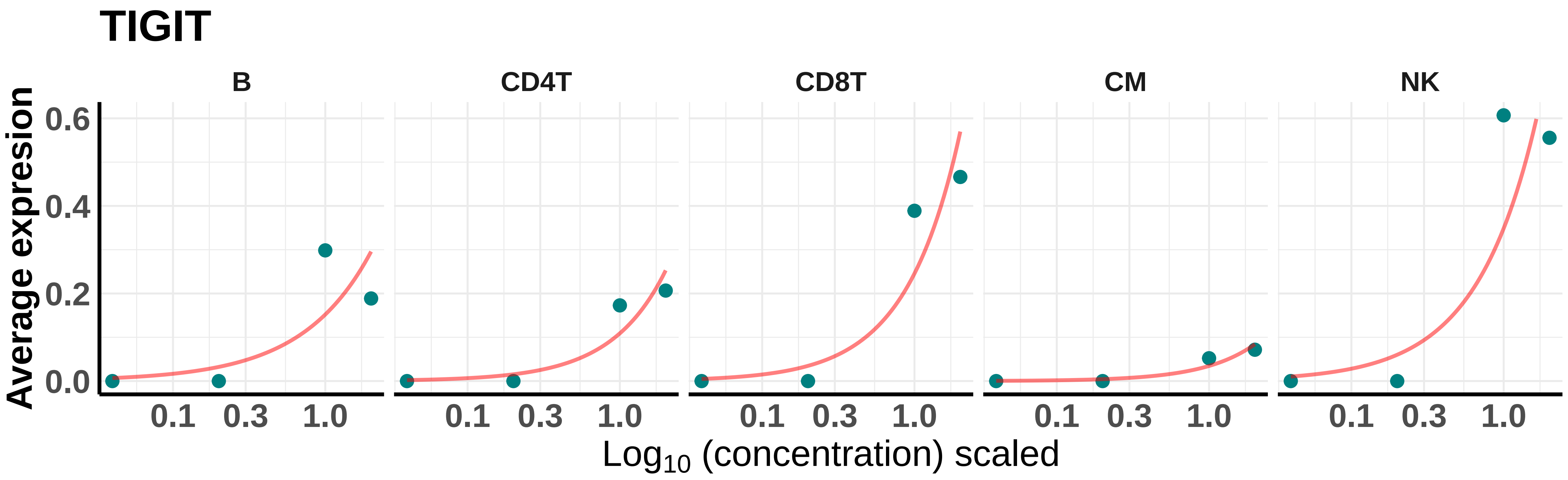
TIGIT
B
CD4T
CD8T
CM
NK
Average expresion
0.0
0.2
0.4
0.6
0.1
0.3
1.0
Log10 (concentration) scaled
